# Risk factors of asthma in the Asian population: a systematic review and meta-analysis

**DOI:** 10.1186/s40101-021-00273-x

**Published:** 2021-12-09

**Authors:** Yang Yie Sio, Fook Tim Chew

**Affiliations:** Allergy and Molecular Immunology Laboratory, Lee Hiok Kwee Functional Genomics Laboratories, Department of Biological Sciences, National University of Singapore, Block S2, Level 5, 14 Science Drive 4, off Lower Kent Ridge Road, 117543 Singapore, Singapore

**Keywords:** Asthma, Review, Risk, Meta-analysis

## Abstract

**Background and objective:**

An increasing trend of asthma prevalence was observed in Asia; however, contributions of environmental and host-related risk factors to the development of this disease remain uncertain. This study aimed to perform a systematic review and meta-analysis for asthma-associated risk factors reported in Asia.

**Methods:**

We systematically searched three public databases (Web of Science, PubMed, and Scopus) in Feb 2021. We only included articles that reported environmental and host-related risk factors associated with asthma in the Asian population. Random-effect meta-analyses were conducted for frequently reported asthma-associated risk factors to provide an overall risk estimate of asthma development.

**Results:**

Of 4030 records obtained from public databases, 289 articles were selected for review. The most frequently reported asthma-associated risk factor was the family history of allergy-related conditions. The random-effect asthma risk estimates (pooled odds ratio, *OR*) were 4.66 (95% confidence interval (*CI*): 3.73–5.82) for the family history of asthma, 3.50 (95% *CI*: 2.62–4.67) for the family history of atopy, 3.57 (95% *CI*: 3.03–4.22) for the family history of any allergic diseases, 1.96 (95% *CI*: 1.47–2.61) for the family history of allergic rhinitis, and 2.75 (95% *CI*: 1.12–6.76) for the family history of atopic dermatitis. For housing-related factors, including the presence of mold, mold spots, mold odor, cockroach, water damage, and incense burning, the random-effect pooled *OR* ranged from 1.43 to 1.73. Other risk factors with significant pooled *OR* for asthma development included male gender (1.30, 95% *CI*: 1.23–1.38), cigarette smoke exposure (1.44, 95% *CI*: 1.30–1.60), cigarette smoking (1.66, 95% *CI*: 1.44–1.90), body mass index (*BMI*)–related parameters (pooled *OR* ranged from 1.06 to 2.02), various types of air pollution (NO_2_, PM10, and O_3_; pooled *OR* ranged from 1.03 to 1.22), and pre- and perinatal factors (low birth weight, preterm birth, and cesarean section; pooled *OR* ranged from 1.14 to 1.32).

**Conclusions:**

The family history of asthma was the most frequently reported risk factor for asthma development in Asia with the highest risk estimate for asthma development. This suggests a major role of the genetic component in asthma pathogenesis. Further study on asthma genetics is required to improve the current understanding of asthma etiology.

**Supplementary Information:**

The online version contains supplementary material available at 10.1186/s40101-021-00273-x.

## Background

Asthma is one of the most common respiratory syndromes affecting more than 300 million individuals worldwide [1, 2]. Based on the findings from the International Study of Asthma and Allergies in Childhood (ISAAC) reported in 1998, the prevalence of asthma in the Asia-Pacific region was lower as compared with the western European and Oceania regions [3]. However, the ISAAC phase III (2007) has reported a reduction in the 12-month prevalence of asthma-related symptoms in western European and Oceania regions, whereas the same prevalence was increased in the Asia-Pacific region. Given the increasing trend of asthma prevalence in the Asia-Pacific region, further understanding of the disease-associated risk factors specific to this region may provide opportunities to develop better prevention and prognostic and therapeutic approaches for asthma disease management.

To date, numerous studies have been conducted to investigate the asthma-associated risk factor. The family history of asthma was frequently identified in disease-affected individuals, suggesting the high heritability nature of asthma development [4, 5]. Environmental and host-related factors such as obesity [6], air pollutant exposures [7, 8], and tobacco smoke exposures [9] have also been found to significantly correlate with asthma susceptibility. Meta-analysis studies were performed to collectively analyze and summarize the overall risk effects of these asthma-associated risk factors [10–12]. However, risk factors summarized in these meta-analyses, including the overall effect sizes estimated, may not be entirely generalizable to the Asian population due to global differences in cultural, lifestyle, socioeconomic, and ethnic backgrounds. Here, we provide an up-to-date review of studies that reported asthma-associated risk factors in the Asian population. The meta-analysis will be performed to evaluate the overall risk estimate for asthma and to provide a better understanding of asthma manifestation in Asia.

## Methods

### Search strategy

The current systematic review study was conducted following the Preferred Reporting Items for Systematic Reviews and Meta-Analyses (PRISMA) guidelines [13, 14]. The PRISMA checklist was included in Table S1. We searched Web of Science, PubMed, and Scopus databases in February 2021, to retrieve all publications related to asthma-associated risk factors. Search terms were listed in Table S2, which included “asthma”, “epidemiology”, “risk”, and the names of 51 Asian countries, dependencies, or other territories.

### Selection criteria

After the process of deduplication and exclusion of irrelevant articles based on titles and abstracts, we retrieved the full text of the remaining articles and screened against the inclusion and exclusion criteria. We included studies that fulfilled both of the criteria: (1) aimed to identify asthma-associated risk factors or asthma comorbidities and (2) have provided an estimation of the effect size of studied risk factors, such as the odds ratio (OR) with corresponding 95% confidence intervals (CIs). Also, we excluded studies that (1) only investigated non-human subjects, (2) only investigated risk factors associated with asthma severity, (3) only examined subjects from non-Asian countries, (4) have unclear study design, and (5) were review or meta-analysis studies. The quality of included studies was further assessed using JBI Critical Appraisal Tool Checklist containing eight criteria [15]. At each of the reviewing stages, the screening of papers and extraction of data was performed by the first author (Sio YY) independently, followed by further discussion with advice from the corresponding author (Chew FT).

### Data retrieval

The following data were extracted from selected articles: names of authors, year of publication, country or region of study, sample size and basic characteristics of the study cohort, study design, disease definition, risk factors, and their corresponding effect sizes (odds ratio), confidence intervals, and *p* values of asthma association.

### Statistical analysis

To perform the random-effect meta-analysis, we extracted the *OR* and 95% *CI* reported from each study of interest. These study findings were combined using the random-effect model with the pooled *OR* and 95% *CI* also computed. We used a chi-square-based test to examine any heterogeneity presented in the pooled risk estimate, with the inconsistency index (*I*^2^) also computed. The funnel plot was drawn based on the standard errors of the reported effect estimates of the risk factors, followed by visual inspection to examine any publication bias. The STATA version 13.0 statistical software was used for all statistical analyses reported in the current study.

## Results

### Study characteristics

**Fig. 1:**
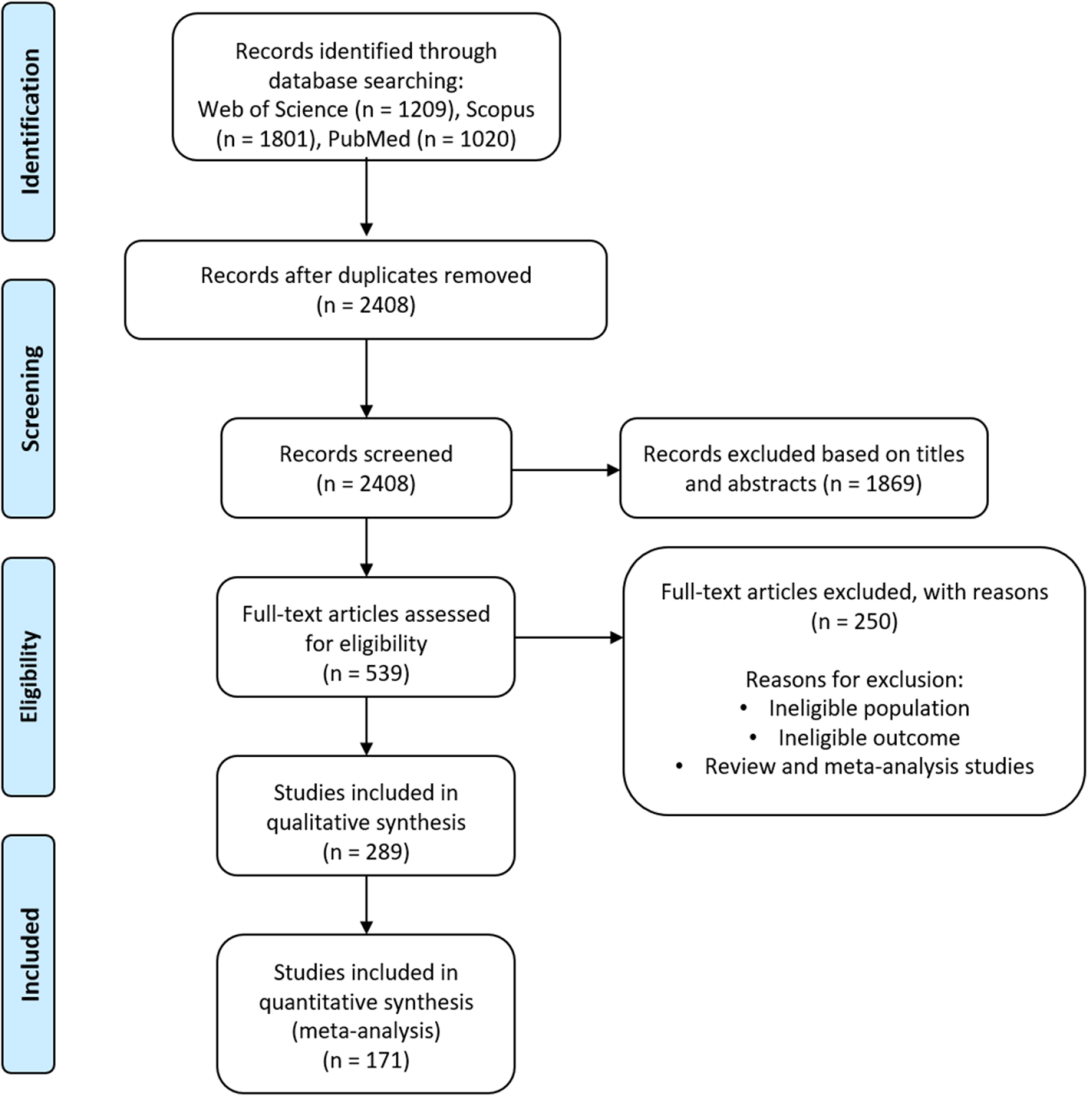
Preferred Reporting Items for Systematic Reviews and Meta-Analyses (PRISMA) flow chart illustrating the study selection procedure for systematic review and meta-analysis on risk factors of asthma in the Asian population

Figure 1 (PRISMA diagram) illustrates the overall search and review process of the current study. The initial literature search using Web of Science, Scopus, and PubMed databases has shortlisted 4030 articles that are potentially relevant to the scope of the current review. After removal of duplicates and screening of titles and abstracts of these search records, 539 articles were selected for full-text review. Finally, 289 papers were included in the systematic review process, and their study characteristics and reported asthma-associated risk factors were summarized in Table S3. Of these, 23 were cohort-based or longitudinal studies, 35 were case–control studies, and 231 were cross-sectional studies (Table S3). For the region of study, 73 out of these 289 reported studies were performed in mainland China, whereas Taiwan and India each contributed 38 and 28 publications, respectively (Table S4). The remaining studies were conducted in 25 other countries or regions in Asia, as summarized in Table S4. Other characteristics of these reviewed studies were mostly heterogeneous, including the definitions of asthma and risk factors, study size, study population, and statistical analysis approach (Table S3).

### Results overview

We identified 31 major categories of asthma-associated risk factors that were reported in at least 3 studies (Table S5). Of these, 15 major categories of asthma risk factors were reported in at least 20 studies, which include family medical history, housing (condition, environment, size, type, etc.), age, gender, cigarette smoke exposure, cigarette smoking, body mass index (*BMI*)–related factors, pet exposure, educational level, urbanization, air pollution, breastfeeding, dietary habits, cooking fume exposure, and socioeconomic status (Table S5). Further, we also identified 9 common asthma comorbidities that were reported in at least 3 studies. These include atopy (26 studies) [16–41], allergic rhinitis (AR, 21 studies) [19, 26, 35, 42–59], respiratory infections (20 studies) [27, 40, 44, 49, 57, 59–73], eczema/atopic dermatitis (AD, 18 studies) [40, 44, 45, 47, 53–59, 62, 70, 72, 74–79], gastroesophageal reflux disease (5 studies) [19, 44, 47, 67, 80], chronic rhinosinusitis (5 studies) [19, 57, 76, 81, 82], food allergy (4 studies) [26, 62, 76, 82], otitis (3 studies) [67, 76, 82], and bronchitis (3 studies) [44, 57, 83] (Table S6).

Results from the random-effect meta-analyses for risk factors including family medical history, housing-related factors, gender, cigarette smoke exposure, cigarette smoking, body mass index (*BMI*), air pollution, and pre- and perinatal factors are shown in Figures S1, S2, S3, S4, S5, S6, S7, S8, S9, S10, S11, S12, S13, S14, S15, S16, S17, S18, S19, S20, S21, S22, S23, S24, S25, S26, S27, S28, S29, S30, S31, S32, S33 and summarized in Fig. 2. These results were also discussed further in the subsequent sections. Besides, meta-analysis was not performed for other risk factors that were also frequently reported, given most studies were heterogeneous on their assessment and analytical approaches for these risk factors.

**Fig. 2:**
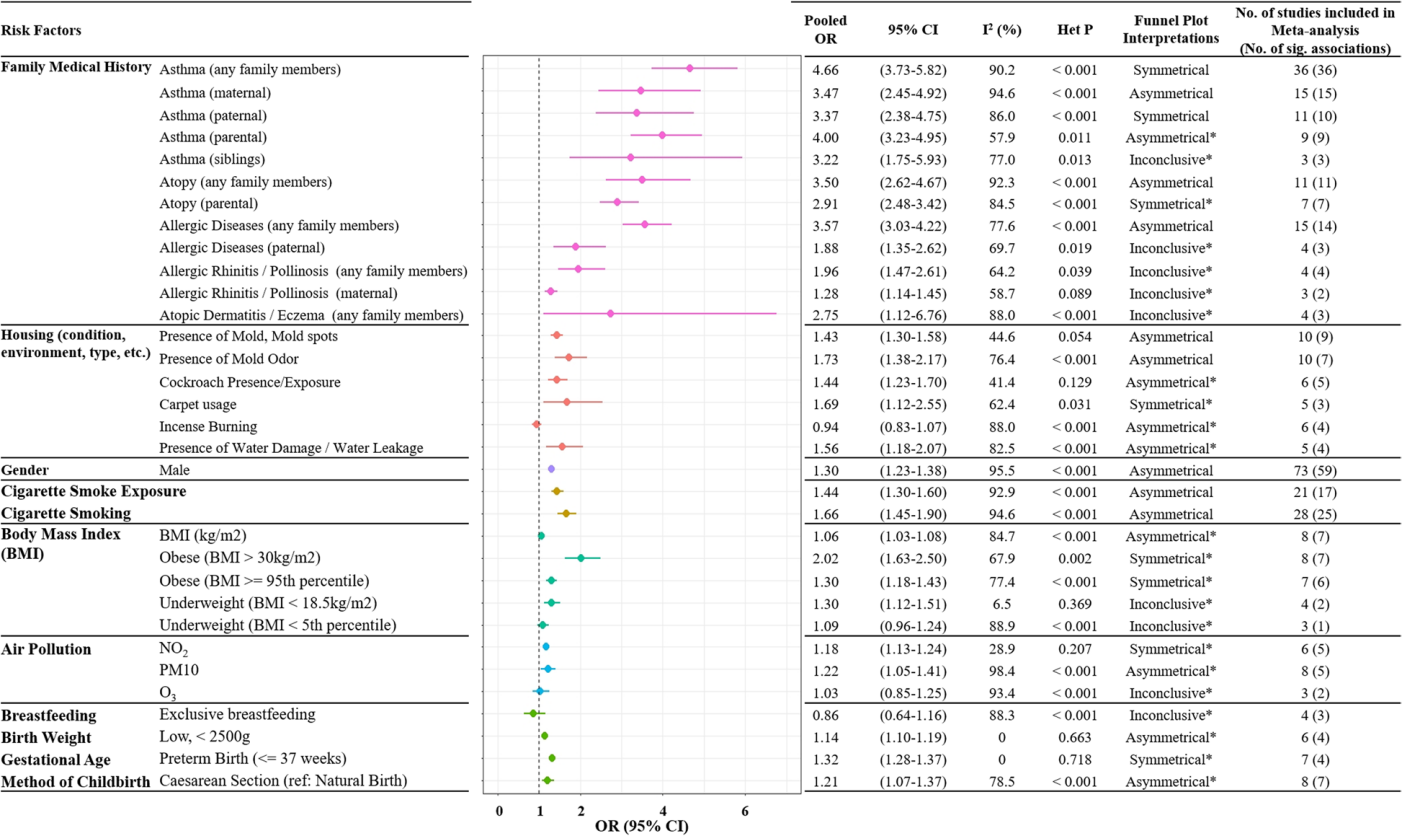
Meta-analyses of risk factors associated with asthma in Asia. The pooled odds ratios (ORs) for each asthma-associated risk factor were computed using the random-effect meta-analysis, with 95% confidence intervals (95% CIs) also included. Results from the heterogeneity test, including the *I*^2^ value and the heterogeneity *p* value (*Het P*) were also included in the figure. Publication biases were assessed based on the symmetry of funnel plots for each meta-analysis. The asterisk (*) indicates an inconclusive interpretation of the funnel plot because of the small number of studies included in the meta-analysis (*n* < 10)

### Family medical history

Overall, 91 studies in Asia investigated the associations between the family history of various allergy-related diseases and the risk of asthma development [20, 22, 23, 25–27, 31, 32, 34–36, 42, 44–46, 51, 52, 57, 59, 62, 65–68, 70–73, 76–79, 82–140]. Among these, the family medical history of asthma (any family members) was most frequently studied and significantly associated with an increased risk of asthma (36 studies) [20, 25–27, 32, 34, 35, 44–46, 51, 57, 59, 76, 77, 79, 82, 83, 86, 95, 101, 102, 104–106, 108–110, 112, 116, 123, 128, 129, 133, 137, 140, 141]. In the random-effect meta-analysis performed for the family history of asthma (any family members) based on these 36 studies, the combined risk estimate for asthma development was increased significantly (pooled *OR* = 4.66, 95% *CI*: 3.73–5.82, *I*^2^ = 90.2%, heterogeneity *p* value < 0.001; Fig. 2 and Fig. S1). Further, we also performed meta-analyses for the family medical history of asthma in specific family groups separately, including paternal asthma (11 studies) [26, 27, 62, 68, 92, 113, 114, 117, 119, 120, 138], maternal asthma (15 studies) [26, 27, 62, 68, 73, 84, 89, 92, 113, 117, 119, 120, 123, 134, 138], parental asthma (9 studies) [26, 66, 72, 78, 88, 93, 118, 121, 122], and sibling’s asthma (4 studies) [26, 65, 123, 138]. The combined risk estimates for asthma were also significantly increased in these four meta-analyses (pooled *OR* ranged between 3.22 and 4; Fig. 2 and Figs. S2, S3, S4, S5). Significant heterogeneities were observed in all random-effect meta-analyses performed for the family medical history of asthma (Fig. 2 and Figs. S1, S2, S3, S4, S5), indicating that these included findings had different study outcomes across each other.

The family history of atopy was frequently associated with an increased risk of asthma in the Asian population (10 studies, Table S5) [36, 71, 94, 96–98, 103, 107, 124, 139]. Using these findings, in the random-effect meta-analysis for the family history of atopy, the combined risk estimate for asthma was significantly increased (pooled *OR* = 3.50, 95% *CI*: 2.62–4.67, *I*^2^ = 92.3%, heterogeneity *p* value < 0.001; Fig. 2 and Fig. S6). Seven studies have further evaluated the risk of asthma development in subjects with parental atopy [23, 85, 111, 119, 127, 135, 136]. In the random-effect meta-analysis using these findings, the combined risk estimate for asthma was also significantly increased (pooled *OR* = 2.91, 95% *CI*: 2.48–3.42, *I*^2^ = 84.5, heterogeneity *p* value < 0.001; Fig. 2 and Fig. S7).

The family medical history of other allergic diseases, such as AR or AD, was also reported as an asthma risk factor in Asia. Four studies have significantly associated the family history of AR with an increase in asthma risk [44, 46, 57, 109]. The pooled *OR*, calculated from the random-effect meta-analysis, also showed an overall increase in asthma risk (1.96, 95% *CI*: 1.47–2.61, *I*^2^ = 64.2%, heterogeneity *p* value = 0.039; Fig. 2 and Fig. S8). Additionally, in the random-effect meta-analysis for the family history of maternal AR (3 studies) [62, 84, 138], the combined risk estimate for asthma was significantly increased (pooled *OR* = 1.28, 95% *CI*: 1.14–1.45, *I*^2^ = 58.7%, heterogeneity *p* value = 0.089; Fig. 2 and Fig. S9). Four studies have investigated the association between the family history of AD and asthma risk [32, 44, 46, 57]. Of these, three studies have shown a significant association between this risk factor and an increased asthma risk [32, 46, 57]. In the random-effect meta-analysis using findings from these four studies, the pooled *OR* was 2.75 (95% *CI*: 1.12–6.76, *I*^2^ = 88%, heterogeneity *p* value < 0.001; Fig. 2 and Fig. S10).

Fifteen studies have collectively analyzed the family history of any allergic disease as a risk factor for asthma [31, 52, 70, 77, 86, 91, 100, 113, 115, 117, 126, 130–132, 140]. Of these, we removed one study [132] from the subsequent meta-analysis due to a different risk factor definition as compared with the other studies. In the random-effect meta-analysis for the family history of any allergic disease (14 studies) [31, 52, 70, 77, 86, 91, 100, 113, 115, 117, 126, 130, 131, 140], the combined odds ratio showed a significant increase in asthma risk (combined OR = 3.57, 95% *CI*: 3.03–4.22, *I*^2^ = 77.6%, heterogeneity *p* value < 0.001; Fig. 2 and Fig. S11). Four studies have investigated the risk of asthma development in subjects with paternal allergic diseases [87, 90, 113, 117]. In the random-effect meta-analysis using these findings, the asthma risk estimate was also increased significantly (combined *OR* = 1.88, 95% *CI*: 1.35–2.62, *I*^2^ = 69.7%, heterogeneity *p* value = 0.019; Fig. 2 and Fig. S12).

### Housing-related factors

A total of 76 studies have investigated housing-related factors and their association with asthma [16–18, 25, 26, 38, 52, 53, 58–60, 67, 72, 73, 79, 82, 85, 88, 91, 94, 95, 97, 98, 105, 111, 118–120, 125, 127, 129, 130, 135, 136, 138, 142–182]. In these studies, frequently investigated housing-related risk factors of asthma included household dampness (18 studies) [17, 67, 72, 82, 85, 88, 125, 130, 142, 143, 146, 147, 150, 157, 166, 171, 174, 180], traffic pollution (14 studies) [16, 18, 53, 58, 127, 129, 130, 135, 148, 149, 172, 173, 176, 181], the presence of mold or mold spots (11 studies) [111, 119, 120, 129, 136, 142, 143, 150, 151, 160, 174], the presence of mold odor (10 studies) [85, 142, 143, 146, 147, 150, 151, 160, 174, 183], cockroach exposures (9 studies) [98, 111, 120, 130, 136, 160, 163, 165, 172], housing type (7 studies) [26, 135, 155, 162, 167, 178, 179], size of housing (5 studies) [26, 105, 127, 142, 155], and carpet usage (5 studies) [125, 130, 135, 149, 160]. Household dampness was associated with an increased asthma risk in 16 studies significantly (Table S5) [17, 67, 72, 82, 85, 88, 130, 142, 143, 146, 147, 150, 157, 166, 171, 180]; however, two other studies reported a mixed or insignificant result for this risk factor [125, 174]. Further, in these reviewed studies, the dampness of the housing environment was assessed by measuring the presence of damp stains [143, 150, 157]; dampness of clothes [142, 143], bed [142, 143], floor [17], or wall [67]; or general household dampness [82, 88, 142, 166, 171]. Meta-analysis was not performed for this risk factor due to the heterogeneity in assessment approaches of household dampness. Nevertheless, meta-analysis was performed for the presence of water damage or leakage in the household environment (5 studies) [111, 136, 142, 154, 174], and the combined random-effect risk estimate for asthma was significantly increased (pooled *OR* = 1.56, 95% *CI*: 1.18–2.07, *I*^2^ = 82.5%, heterogeneity *p* value < 0.001; Fig. 2 and Fig. S13).

The presence of mold, mold spots, or mold odor in the household environment was reported to be significantly associated with a greater risk of developing asthma in 12 studies [111, 119, 120, 129, 136, 142, 143, 146, 147, 150, 151, 160]. By contrast, four other studies have shown a mixed or insignificant association with asthma for this risk factor [85, 174, 183]. Further, 11 out of 12 studies that showed significant findings for this risk factor were all conducted in mainland China [142, 143, 146, 147, 150, 151] and Taiwan [111, 119, 120, 136, 160]. This may suggest an ethnic- or region-specific association of this risk factor with asthma. In the random-effect meta-analysis for the presence of mold or mold spot in the house based on 10 studies [111, 119, 120, 129, 136, 143, 150, 151, 160, 174], the combined asthma risk estimate was increased (pooled *OR* = 1.43, 95% *CI*: 1.30–1.58, *I*^2^ = 44.6%, heterogeneity *p* value = 0.054; Fig. 2 and Fig. S14). In the random-effect meta-analysis for the presence of mold odor based on 10 studies [85, 142, 143, 146, 147, 150, 151, 160, 174, 183], a similar trend of increasing combined asthma risk estimate was also observed (pooled *OR* = 1.73, 95% *CI*: 1.38–2.17, *I*^2^ = 76.4%, heterogeneity *p* value < 0.001; Fig. 2 and Fig. S15).

Six studies have examined the overall presence of cockroaches in the household [98, 111, 130, 160, 165, 172]; five of these studies [98, 111, 160, 165, 172] showed a significantly higher risk of developing asthma in the presence of this risk factor. In the random-effect meta-analysis for the presence of cockroaches in the household environment (6 studies) [98, 111, 130, 160, 165, 172], the combined risk estimate for asthma was increased (pooled *OR* = 1.44, 95% *CI*: 1.23–1.70, *I*^2^ = 41.4%, heterogeneity *p* value = 0.129; Fig. 2 and Fig. S16). Further, four studies have compared different frequencies of cockroach exposure in the household, and all have shown significant associations with asthma for increased exposure frequencies [120, 136, 160, 163].

The usage of carpet in the housing environment was significantly associated with an increased risk of asthma as reported in three studies [130, 149, 160], while two studies [125, 135] reported mixed or insignificant associations for this risk factor. In the random-effect meta-analysis for the usage of carpet in the household environment (5 studies) [125, 130, 135, 149, 160], the combined risk estimate for asthma was increased (pooled *OR* = 1.69, 95% *CI*: 1.12–2.55, *I*^2^ = 62.4%, heterogeneity *p* value = 0.031; Fig. 2 and Fig. S17). Incense burning was also frequently studied as a household risk factor contributing to asthma; three studies significantly associated incense burning with decreased asthma risk [38, 120, 170], while one study has associated this factor with increased asthma risk [172] separately. Further, two other studies have reported a mixed or insignificant association between incense burning and asthma [125, 182]. In the meta-analysis for incense burning (6 studies) [38, 120, 125, 170, 172, 182], the combined risk estimates for asthma is not significant (pooled *OR* = 0.94, 95% *CI*: 0.83–1.07, *I*^2^ = 88%, heterogeneity *p* value < 0.001; Fig. 2 and Fig. S18).

Lastly, meta-analysis was not performed for the presence of traffic pollution or traffic exposure near the housing environment, given multiple studies have used different assessment approaches for this risk factor. However, all ten studies that reported significant findings have consistently associated traffic pollution or exposure with an increased risk of asthma development [16, 18, 53, 58, 127, 129, 130, 148, 149, 172]. Also, meta-analysis was not performed for other frequently studied housing-related asthma risk factors, including the type and size of housing, due to the same reason of heterogeneity in assessment approaches.

### Gender

The association between gender and asthma was reported in 75 studies [16, 18, 19, 21, 22, 33, 35, 37, 38, 40, 42, 43, 47, 49, 56, 58, 62, 67, 70, 71, 73, 74, 79, 82–87, 92, 93, 95, 97, 99, 104, 105, 107, 110, 116, 118, 120, 122, 124–127, 130, 131, 133, 135–137, 139, 142, 159, 170, 179, 184–201]. Of these, 58 studies have observed male subjects having a higher asthma susceptibility as compared with that of the female subjects significantly [16, 18, 22, 37, 38, 40, 47, 56, 58, 62, 67, 70, 71, 73, 74, 79, 82–84, 86, 87, 92, 95, 97, 99, 104, 105, 107, 116, 118, 120, 122, 124–127, 130, 131, 135, 136, 139, 142, 159, 170, 179, 184–194, 197–200]. By contrast, 15 studies showed females having higher asthma risk than males significantly [19, 21, 33, 35, 42, 49, 85, 93, 110, 133, 137, 195, 196, 201]. Of these 75 studies, we removed 2 studies [125, 126] from the subsequent meta-analysis due to their missing information in risk factor definition and inconsistency in disease definition. In the random-effect meta-analysis for gender (73 studies), the combined risk estimate for the male developing asthma was increased (pooled *OR* = 1.30, 95% *CI*: 1.23–1.38, *I*^2^ = 95.5%, heterogeneity *p* value < 0.001; Fig. 2 and Fig. S19).

### Cigarette smoke exposure and cigarette smoking

The effect of passive cigarette smoke exposure on the risk of asthma was frequently studied in Asia (63 studies) [19, 27, 36, 38, 40, 43, 52–54, 58, 60, 62, 66, 67, 70, 73, 77–79, 87, 90, 92, 93, 100, 101, 104, 105, 107, 111, 114, 116, 119, 120, 124, 125, 135, 136, 139, 142, 145, 147, 148, 160, 162, 168, 170, 172, 179, 182, 183, 198–200, 202–210]. These studies have used different analytical methods to assess its influence on the susceptibility to asthma, including the number of cigarettes exposed per day [27, 54, 100, 120, 160, 208], the number of persons smoking in the house [53, 105, 114, 207], smoking in the presence of the subject [168], the duration of exposure [93, 160, 168, 207], the exposure during mother’s pregnancy [52, 60, 104, 111, 160, 168], the avoidance of cigarette smoke exposure [119], or the presence of father, mother, or any family member who is a smoker [67, 116, 124, 147, 148, 206]. We included findings from 21 studies in the random-effect meta-analysis for the overall associations of passive cigarette smoke exposure with asthma [19, 43, 62, 66, 77, 90, 92, 101, 107, 111, 127, 136, 142, 162, 170, 179, 183, 202–205]. In this meta-analysis, the combined risk estimate for asthma was significantly increased (pooled *OR* = 1.44, 95% *CI*: 1.30–1.60, *I*^2^ = 92.9%, heterogeneity *p* value < 0.001; Fig. 2 and Fig. S20).

A total of 36 studies have investigated the association between cigarette smoking and asthma [26, 35, 38, 42–44, 49, 51, 54, 56, 61, 90, 100, 101, 112, 125, 128, 139, 142, 162, 167, 170, 179, 185, 196, 198, 200, 203, 209–216]. These studies have used different approaches in the definition of active cigarette smoking, including ever actively smoking [162, 167, 216], former smokers [101, 128, 185, 214], or current smokers [26, 42–44, 49, 51, 54, 56, 61, 90, 100, 101, 112, 125, 128, 142, 170, 196, 203, 211–214]. We included findings from 21 studies in the random-effect meta-analysis for the overall associations of cigarette smoking with asthma [26, 38, 42–44, 49, 51, 54, 56, 61, 90, 100, 101, 112, 128, 139, 142, 170, 179, 196, 198, 200, 203, 211–215]. In this meta-analysis, the combined risk estimate for asthma was increased (pooled *OR* = 1.66, 95% *CI*: 1.45–1.90, *I*^2^ = 94.6%, heterogeneity *p* value < 0.001; Fig. 2 and Fig. S21).

Collectively, our analysis indicated that exposure to cigarette smoke, either via passive exposure or cigarette smoking, was both associated with an overall increase in asthma risk within the Asian population.

### Body mass index (BMI)

A total of 37 studies in Asia have investigated the association between *BMI* and asthma [19, 38, 42–45, 47, 49, 61, 125, 135, 153, 162, 167, 185, 186, 196, 200, 201, 204, 213, 217–232]. These studies have used different *BMI* cut-offs for defining overweight, obesity, and underweight status, while other studies have also analyzed *BMI* as a continuous variable (Table S5). In the random-effect meta-analysis for *BMI* as a continuous variable (8 studies) [38, 43, 49, 135, 153, 201, 204, 230], the overall risk estimate for asthma was increased (pooled *OR* = 1.06, 95% *CI*: 1.03–1.08, *I*^2^ = 84.7%, heterogeneity *p* value < 0.001; Fig. 2 and Fig. S22). This suggests an increase in *BMI* was associated with an increase in asthma risk. Eight studies have also investigated the association between obese (*BMI* > 30 kg/m^2^) and asthma development [162, 167, 185, 196, 213, 217, 222, 225]. In the random-effect meta-analysis using these findings, the asthma risk estimate was increased for this risk factor (pooled *OR* = 2.02, 95% *CI*: 1.63–2.50, *I*^2^ = 67.9%, heterogeneity *p* value = [77, 233] 0.002; Fig. 2 and Fig. S23). The association between obesity (*BMI* ≥ 95th percentile) and asthma was also frequently studied (7 studies) [45, 219, 221, 223, 226, 231, 232]. In the random-effect meta-analysis for this factor, the asthma risk estimate was increased (pooled *OR* = 1.30, 95% *CI*: 1.18–1.43, *I*^2^ = 77.4%, heterogeneity *p* value < 0.001; Fig. 2 and Fig. S24).

Four studies have investigated the association between underweight (*BMI* < 18.5 kg/m^2^) and asthma [42, 167, 185, 217]. In the random-effect meta-analysis for this factor, the overall asthma risk estimate was increased (pooled *OR* = 1.30, 95% *CI*: 1.12–1.51, *I*^2^ = 6.5%, heterogeneity *p* value = 0.369; Fig. 2 and Fig. S25). However, in the random-effect meta-analysis for 3 others studies that used *BMI* < 5th percentile as the definition of underweight [223, 231, 232], the overall risk estimate for asthma was not significantly changed (pooled *OR* = 1.09, 95% *CI*: 0.96–1.24, *I*^2^ = 88.9%, heterogeneity *p* value < 0.001; Fig. 2 and Fig. S26).

### Air pollution

There were 24 studies that investigated the associations between different types of air pollution and the risk of developing asthma. These air pollution-related parameters included the levels of NO_2_ (9 studies) [127, 135, 172, 182, 234–238], particulate matter less than 10 μm (PM10, 11 studies) [47, 54, 127, 135, 136, 172, 182, 187, 235, 236, 239], PM2.5 (3 studies) [42, 234, 240], O_3_ (4 studies) [54, 135, 136, 234], CO (3 studies) [54, 136, 234], nitrogen oxides (3 studies) [136, 241, 242], and SO_2_ (5 studies) [135, 136, 234, 236, 238]. In the random-effect meta-analysis for NO_2_ pollution (6 studies) [127, 135, 172, 182, 234, 235], the overall asthma risk estimate was increased (pooled *OR* = 1.18, 95% *CI*: 1.13–1.24, *I*^2^ = 88.9%, heterogeneity *p* value < 0.001; Fig. 2 and Fig. S27). In the random-effect meta-analysis for PM10 pollution (8 studies) [47, 54, 127, 135, 172, 182, 187, 235], the overall asthma risk estimate was increased (pooled *OR* = 1.22, 95% *CI*: 1.05–1.41, *I*^2^ = 98.4%, heterogeneity *p* value < 0.001; Fig. 2 and Fig. S28). In the random-effect meta-analysis for O_3_ pollution (3 studies) [54, 135, 234], the overall asthma risk estimate was not significantly changed (pooled *OR* = 1.03, 95% *CI*: 0.85–1.25, *I*^2^ = 93.4%, heterogeneity *p* value < 0.001; Fig. 2 and Fig. S29). Meta-analysis was not performed for other types of air pollution as most studies were heterogeneous on their assessment approaches of air pollution level.

### Pre- and perinatal factors

Multiple pre- and perinatal factors were also frequently studied as a risk factor contributing to asthma, including breastfeeding (26 studies) [27, 36, 40, 44, 64, 65, 69, 70, 77, 85, 86, 89, 111, 122, 126, 127, 131, 133, 135, 138, 153, 199, 243–246], birth weight (17 studies) [66, 69, 84, 85, 87, 126, 127, 130, 132, 133, 183, 199, 228, 247, 248], gestational age (11 studies) [36, 69, 77, 84, 85, 126, 133, 140, 233, 247, 248], and the method of childbirth (10 studies) [36, 47, 64, 84, 126, 131, 133, 249–251]. Given most studies were heterogeneous on their assessment approaches and analytical methods for these risk factors, meta-analyses were only performed for exclusive breastfeeding, low birth weight (< 2500 g), preterm birth (≤ 37 weeks), and childbirth by caesarean section (reference category: natural birth). In the random-effect meta-analysis for exclusive breastfeeding (4 studies) [64, 77, 122, 199], the overall asthma risk estimate was not significantly changed (pooled *OR* = 0.86, 95% *CI*: 0.64–1.16, *I*^2^ = 88.3%, heterogeneity *p* value < 0.001; Fig. 2 and Fig. S30). In the random-effect meta-analysis for low birth weight (< 2500 g, 6 studies) [69, 84, 87, 127, 199, 247], the overall asthma risk estimate was increased (pooled *OR* = 1.14, 95% *CI*: 1.10–1.19, *I*^2^ = 0%, heterogeneity *p* value = 0.663; Fig. 2 and Fig. S31). In the random-effect meta-analysis for preterm birth (≤ 37 weeks, 6 studies) [77, 84, 85, 140, 233, 247], the overall asthma risk estimate was increased (pooled *OR* = 1.32, 95% *CI*: 1.28–1.37, *I*^2^ = 0%, heterogeneity *p* value = 0.718; Fig. 2 and Fig. S32). In the random-effect meta-analysis for childbirth by caesarean section (8 studies) [36, 47, 84, 126, 131, 133, 249, 250], the overall asthma risk estimate was increased (pooled *OR* = 1.21, 95% *CI*: 1.07–1.37, *I*^2^ = 78.5%, heterogeneity *p* value < 0.001; Fig. 2 and Fig. S33).

### Publication bias

Publication bias was assessed using a funnel plot for each of the 33 meta-analyses performed in this current study (Fig. 2 and Figs. S1, S2, S3, S4, S5, S6, S7, S8, S9, S10, S11, S12, S13, S14, S15, S16, S17, S18, S19, S20, S21, S22, S23, S24, S25, S26, S27, S28, S29, S30, S31, S32, S33). Of these, 23 meta-analyses have insufficient studies (*n* < 10) to be comprehensively analyzed for publication bias. Of the remaining 10 meta-analyses, symmetrical funnel plots were observed for the analyses for the overall family history of asthma and paternal asthma (Fig. 2, Figs. S1, and S3). However, for the analyses for 8 other risk factors (maternal asthma, overall family history of atopy, overall family history of allergic diseases, household presence of mold, household presence of mold odor, male gender, cigarette smoke exposure, and cigarette smoking), these funnel plots were asymmetrical, suggesting publication biases (Fig. 2, Figs. S2, S6, S8, S13, S14, S19, S20, S21).

### Other factors not included in meta-analysis

Meta-analysis was not performed for multiple asthma risk factors that were frequently reported, including parental or participant’s educational level, pet exposure, urbanization, and dietary habits, given these factors were assessed differently among reviewed studies. Overall, seven studies reported that a higher educational level of the participant was significantly associated with a lower risk of asthma [42, 49, 162, 167, 196, 213, 216], while 11 studies have provided mixed or insignificant findings [26, 125, 178, 179, 197, 252] (Table S5) [42, 49, 162, 167, 196, 213, 216]. Besides, five studies reported that a higher parental educational level is significantly associated with a lower risk of asthma [31, 58, 86, 93, 100], while eight studies reported that a lower parental educational level is significantly associated with a lower risk of asthma [38, 47, 54, 104, 120, 126, 138, 170]. Five studies reported mixed or insignificant associations between parental educational level and asthma risk.

The associations between pet exposures and asthma risk were investigated in 33 studies [16, 18, 27, 36, 44, 58, 66, 67, 71, 77, 79, 90, 97, 101, 107, 113, 117, 119, 125, 130, 132, 135, 139, 160, 165, 169, 172, 178, 199, 204, 253–255]. Of these findings, an increased risk of asthma was significantly associated with the exposure to cats (7 studies) [27, 58, 90, 113, 117, 172, 253] or dogs (7 studies) [16, 44, 71, 90, 113, 117, 160]. A study had also associated the exposure to both cats and dogs with increasing asthma susceptibility [18]. Multiple studies have also associated asthma with exposure to a specific group of animals, such as farm animals [113, 117, 169], furred pets [101, 204, 254], or overall pet animals [44, 66, 67, 97, 107, 117, 119]. Stratification of the study cohort based on the duration or frequency of exposure [27, 160], number of pets owned [113, 165], or exposure to animals at a specific stage of life [27, 113, 165, 253] was also reported to be significantly associated with asthma development. Overall, in most studies, exposure to animals was shown to associate with an increased risk of asthma, whereas only five studies have shown the asthma-protective effect from pet or farm animal exposures [66, 119, 132, 169, 254].

Further, 28 studies have investigated and compared the effect of living in urban, suburban, or rural areas on the risk of developing asthma [31, 42, 43, 49, 54, 62, 78, 84, 87, 100, 105, 107, 121, 127, 129, 142, 157, 172, 179, 189–192, 197, 199, 213, 235, 256]. Increasing urbanization level was shown to be significantly associated with increased asthma risk in 17 studies [31, 54, 84, 100, 107, 121, 127, 129, 142, 157, 172, 190–192, 213, 235, 256], whereas an opposite trend of decreasing asthma risk due to this increase was shown in four studies [43, 49, 105, 197].

The associations between dietary habits and asthma were studied and reported in 23 articles [16, 18, 23, 31, 47, 65, 71, 77, 86, 91, 96, 153, 166, 191, 195, 196, 200, 256–261]. Of these, an increased risk of developing asthma was significantly associated with the consumption of meat (chicken, red meat, etc.) (6 studies) [71, 86, 153, 195, 196, 257] or junk foods (2 studies) [18, 257], whereas fruit consumption was correlated to reduced asthma risk (6 studies) [16, 96, 166, 196, 200, 257]. However, for the other types of food consumption, contradictory findings were often observed from the literature. For instance, dairy product consumption was associated with either an increase [23, 86] or a decrease [16, 31, 71, 195, 196] in asthma risk. Similarly, contradictory findings were also reported on the effects of consumption of seafood (including fish) [16, 256, 259, 260] and vegetables [16, 31, 196] on the risk of developing asthma.

The association between asthma and cooking fume exposure was investigated and reported in 21 studies [16, 26, 42, 101, 116, 118, 130, 155, 166–168, 172, 175, 178, 179, 181, 182, 212, 213, 215, 262]. Increasing risk of developing asthma was reported for various routes of exposure to cooking fumes, including the exposure to direct oil fumes [101], cooking without a chimney or a fan [168, 213], eating in the kitchen [116], and cooking in the house without a separate kitchen [167, 179, 212]. Five studies have further shown the usage of wood [16, 155], coal [101, 215, 262], gas [118, 130, 166], high-pollution fuels [167], fuel mix [179], and biomass/solid fuels [179, 212, 213] as cooking fuels for household cooking was associated with increased asthma risk, as compared with the usage of low-pollution fuels such as electricity. Seven studies reported a mixed or insignificant association between cooking fume exposure and asthma [26, 42, 172, 175, 178, 181, 182].

Lastly, the association between socioeconomic status and asthma was studied and reported in 21 articles [33, 40, 47, 62, 68, 70, 78, 88, 105, 115, 125, 133, 162, 178, 179, 197–199, 212, 232, 252]. This risk factor was assessed differently as income [33, 40, 47, 62, 68, 70, 78, 105, 115, 125, 133, 178, 198, 199], standard of living index [162, 179, 252], socioeconomic status [232], financial standing [88], wealth index [212], or wealth category [197] across multiple studies. Increasing socioeconomic status was shown to be significantly associated with increased asthma risk in four studies [33, 40, 47, 232], whereas an opposite trend of decreasing asthma risk due to this increase was shown in nine studies [68, 88, 105, 115, 133, 162, 179, 199, 212]. Also, eight studies reported a mixed or insignificant association between socioeconomic factor and asthma [62, 70, 78, 125, 178, 197, 198, 252].

## Discussion

The current systematic review and meta-analysis study aimed to summarize and estimate the overall risk estimates of frequently reported asthma risk factors in the Asian population. We included 289 studies that were published from the year 1993 to 2021. In these studies, 15 major categories of asthma risk factors were reported in at least 20 studies, including family medical history, housing (condition, environment, size, type, etc.), age, gender, cigarette smoke exposure, cigarette smoking, *BMI*-related factors, pet exposure, educational level, urbanization, air pollution, breastfeeding, dietary habits, cooking fume exposure, and socioeconomic status. For most of these risk factors, we conducted random-effect meta-analyses and demonstrated overall significant associations between these factors and asthma in the Asian population. To our knowledge, this is the most up-to-date systematic review and meta-analysis of asthma-associated risk factors in Asia. The current study identified major factors that are frequently and significantly associated with the manifestation of asthma in this region. Further, these asthma risk factors can be divided into modifiable and non-modifiable factors to be used as an effective target of asthma preventive medicine. Modifiable factors include housing (condition, environment, size, type, etc.), cigarette smoke exposure, cigarette smoking, *BMI*-related factors, pet exposure, educational level, urbanization, air pollution, breastfeeding, dietary habits, cooking fume exposure, and socioeconomic status. These factors can be targeted in primary asthma preventive measures that focus on the prevention of disease development. Non-modifiable asthma risk factors, including family medical history, age, and gender, can be used as a target in secondary and tertiary asthma preventive measures that focus on early disease detection and reduction of disease severity.

Overall, the family medical history of various allergy-related conditions was most frequently studied and reported to be significantly associated with the risk of asthma development. Of these family medical conditions, frequently reported was the family history of asthma, which was found to significantly associate with asthma development in 37 studies performed in Asia (Table S5) [20, 25–27, 32, 34, 35, 44–46, 51, 57, 59, 76, 77, 79, 82, 83, 86, 95, 101, 102, 104–106, 108–110, 112, 116, 123, 125, 128, 129, 133, 137, 140, 141]. Our findings are in concordance with the meta-analysis result performed previously using 6 independent studies, which showed an overall increase in asthma risk for preschool children with a family history of asthma (pooled *OR* = 2.20, 95% *CI*: 1.54–3.14) [12]. This suggests a high heritability of asthma and the genetic component may underlie the disease pathogenesis process. Multiple asthma candidate genes have been discovered to date, with the heritability of this disease estimated to range from 35 to 95% [263–266]. Nevertheless, the genetic pathway leading to asthma development is not well understood and should be explored further to improve the current understanding of asthma pathogenesis.

In this meta-analysis study, we also observed overall significant associations between asthma and multiple housing-related risk factors, including housing dampness, presence of water damage, carpet usage, and exposures to mold and cockroaches. Indoor dampness and the presence of mold in the household were shown to associate with increased asthma risk in a previously conducted meta-analysis study [267]. This indicates allergenic sensitizations towards fungal spores and conidia might associate with asthma development, which is in concordance with previous epidemiological and immunological evidence [268–270]. Similarly, the usage of carpet in the household environment might also increase an individual’s sensitization to house dust mite allergens, which was consistently shown to increase the risk of developing asthma in the tropical region of Asia [32, 271–273]. Lastly, sensitization to cockroaches was also frequently reported as an important risk factor for asthma (reviewed in [274]). Given these consistent associations reported in several studies, action should be taken to reduce the respective allergen load in the household environment to decrease the risk of asthma development.

This current meta-analysis focused on studies conducted in Asia. By comparing our results to meta-analyses that were focused on the general global outcomes, multiple region-specific risk factors were observed. For instance, an overall increase in asthma risk was associated with black carbon pollution in a previous meta-analysis [11], while this current meta-analysis did not observe any study reporting this association. The protective effect of exclusive breastfeeding against asthma was shown to be significant using meta-analysis [275]; however, this current study did not show a significant association (potentially due to the combined sample size). Besides, the overall asthma risk was increased for household water damage in our current meta-analysis; however, a previous meta-analysis [267] did not show a significant overall association for this risk factor, suggesting differential environmental factors may be more predominant in Asia. Exposure to cats was shown to significantly reduce the risk of asthma in a previous meta-analysis [276]. Although we did not perform a meta-analysis on this risk factor due to the heterogeneity across studies, those that evaluated this factor have shown an increased risk for asthma associated with exposure to cats. Collectively, these observations suggest findings from previous global meta-analyses may not entirely be generalizable to the Asian population, and slight variations may occur.

Our study has several limitations that should be addressed. First, in the meta-analyses for most of the risk factors, the number of studies included was too small (*n* < 10) for the comprehensive assessment of publication bias using the funnel plot [277]. Besides, we also detected a significant level of heterogeneity in most of the meta-analyses performed. This may be due to the differences in cultural, lifestyle, geographical, and ethnic background that may influence the associations between most factors and asthma. Additional study is therefore required to further validate these identified factors that were associated with asthma in Asia.

## Conclusion

In conclusion, the current review study has identified multiple environmental and host-related asthma risk factors in the Asian population. The risk factors identified in our meta-analysis can improve the current understanding of asthma etiology and develop better preventive, therapeutic, and prognostic approaches for asthma.

## Supplementary Information

**Additional file 1: Figures S1–S33.** Forest plots and funnel plots for random-effect meta-analysis of the different asthma risk factors.

**Additional file 2: Table S1.** PRISMA 2009 Checklist.

**Additional file 3: Table S2.** Keywords used to perform literature search in three publication databases (Web of Science, Scopus, and Pubmed) to retrieve articles reporting asthma-associated risk factors in Asia.

**Additional file 4: Table S3.** Study characteristics and reported asthma-associated risk factors of 289 studies included in the systematic review process.

**Additional file 5: Table S4.** Reported publications on asthma-associated risk factors from countries, dependencies, or other territories within Asia (1993-2021).

**Additional file 6: Table S5.** Summary of frequently reported asthma-associated risk factors in the Asian population (1993-2021).

**Additional file 7: Table S6.** Summary of frequently reported asthma comorbidities in the Asian population (1993-2021).

## Data Availability

All data used and included in this study are available from the corresponding author (Chew Fook Tim).

## Abbreviations

AR: Allergic rhinitis
AD: Atopic dermatitis
BMI: Body mass index
CI: Confidence interval
ISAAC: International Study of Asthma and Allergies in Childhood
OR: Odds ratio
PM10: Particulate matter less than 10 μm
PM2.5: Particulate matter less than 2.5 μm
PRISMA: Preferred Reporting Items for Systematic Reviews and Meta-Analyses
