# Supplementary material for: Risk factors of asthma in the Asian population: a systematic review and meta-analysis
Source: J Physiol Anthropol. 2021 Dec 9;40:22. doi: 10.1186/s40101-021-00273-x (PMC8662898; doi:10.1186/s40101-021-00273-x)
Supplement: Supplementary file 1 — Additional file 1: Figures S1–S33. Forest plots and funnel plots for random-effect meta-analysis of the different asthma risk factors. [file 40101_2021_273_MOESM1_ESM.docx]

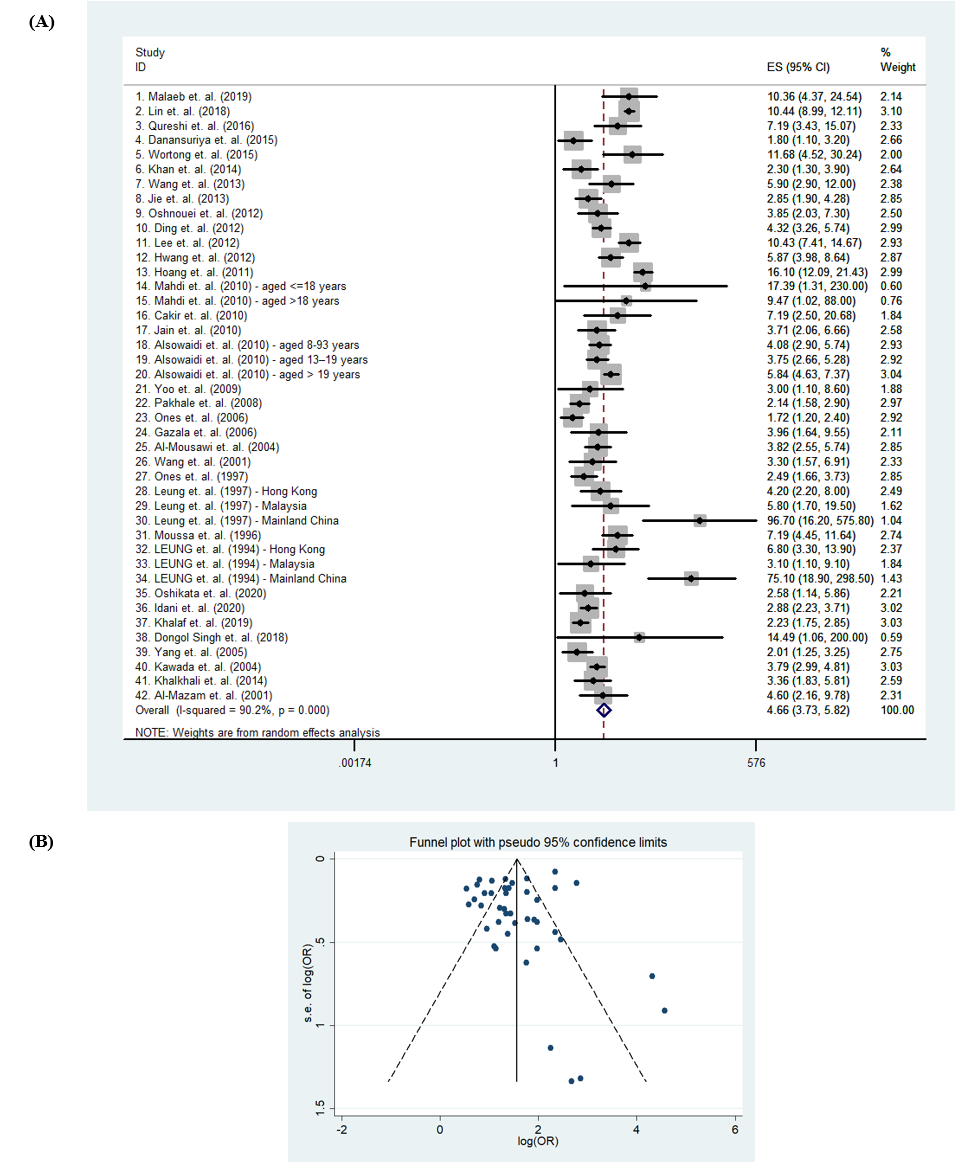


Figure S1. **(A)** Forest Plot and **(B)** Funnel Plot for Random-Effect Meta-Analysis for the Family Medical History of Asthma (any family members) as Asthma Risk Factor Reported in 36 Independent Studies. Effect size (ES) is represented by the odds ratio (OR) and 95% confidence interval (CI) reported in each study. Results from the heterogeneity test, including the I^2^ value and the heterogeneity p-value (Het P) were also included in the figure. The funnel plot was plotted using the log(OR) and standard error (s.e.) of the log(OR) values from each study.


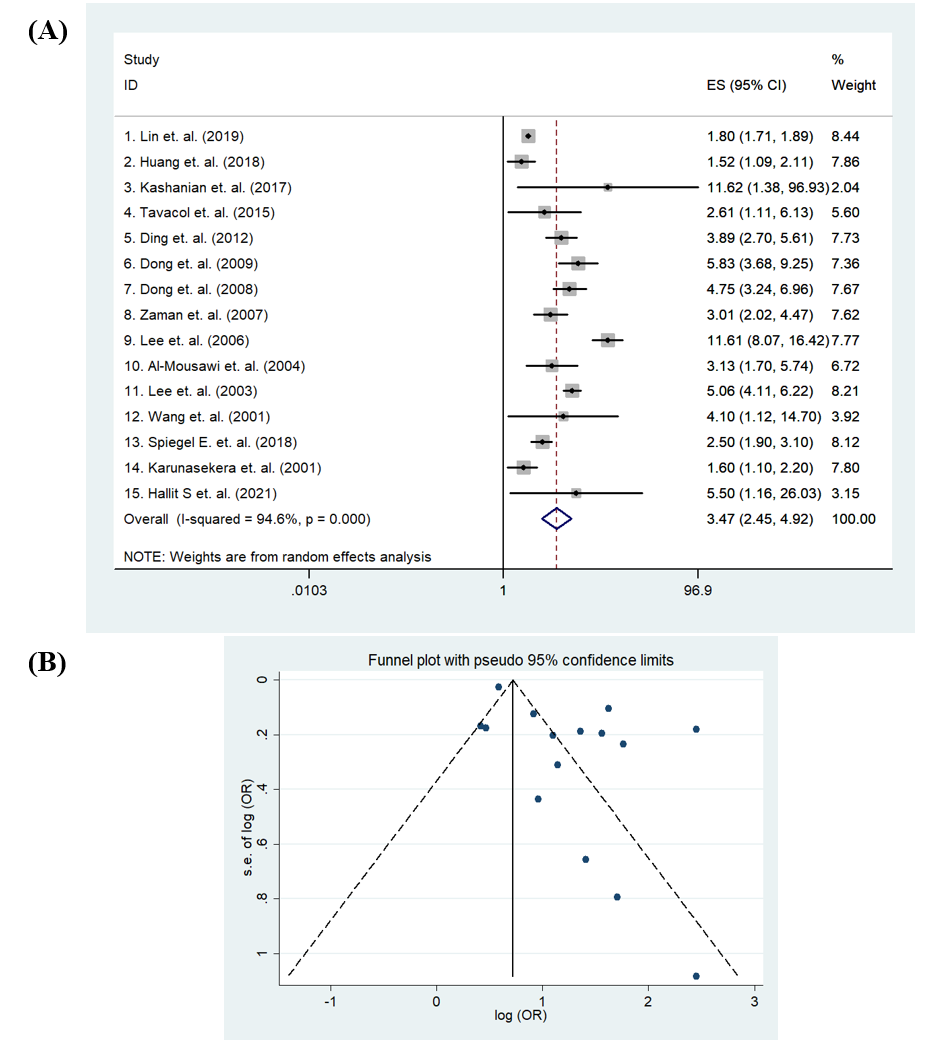


Figure S2. **(A)** Forest Plot and **(B)** Funnel Plot for Random-Effect Meta-Analysis for the Family Medical History of Maternal Asthma as Asthma Risk Factor Reported in 15 Independent Studies. Effect size (ES) is represented by the odds ratio (OR) and 95% confidence interval (CI) reported in each study. Results from the heterogeneity test, including the I^2^ value and the heterogeneity p-value (Het P) were also included in the figure. The funnel plot was plotted using the log(OR) and standard error (s.e.) of the log(OR) values from each study.


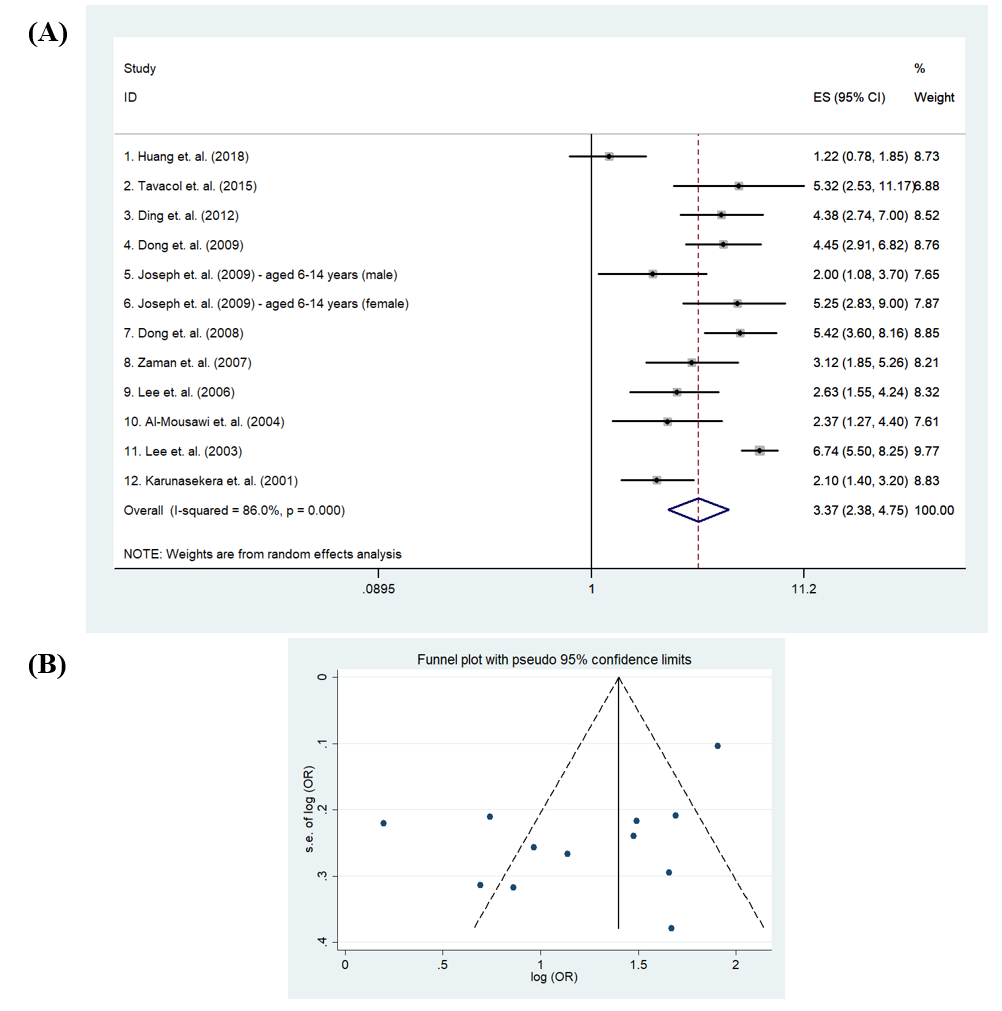


Figure S3. **(A)** Forest Plot and **(B)** Funnel Plot for Random-Effect Meta-Analysis for the Family Medical History of Paternal Asthma as Asthma Risk Factor Reported in 11 Independent Studies. Effect size (ES) is represented by the odds ratio (OR) and 95% confidence interval (CI) reported in each study. Results from the heterogeneity test, including the I^2^ value and the heterogeneity p-value (Het P) were also included in the figure. The funnel plot was plotted using the log(OR) and standard error (s.e.) of the log(OR) values from each study.


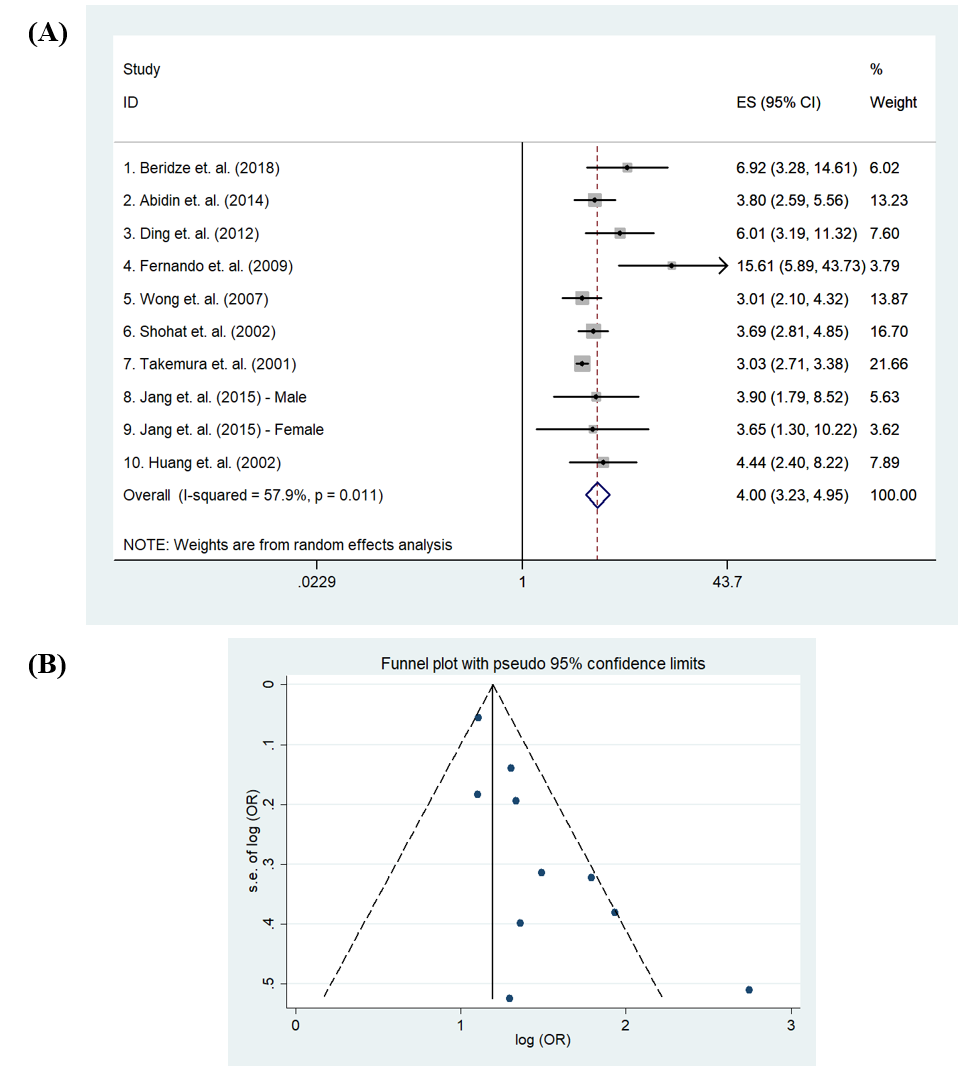


Figure S4. **(A)** Forest Plot and **(B)** Funnel Plot for Random-Effect Meta-Analysis for the Family Medical History of Parental Asthma as Asthma Risk Factor Reported in 9 Independent Studies. Effect size (ES) is represented by the odds ratio (OR) and 95% confidence interval (CI) reported in each study. Results from the heterogeneity test, including the I^2^ value and the heterogeneity p-value (Het P) were also included in the figure. The funnel plot was plotted using the log(OR) and standard error (s.e.) of the log(OR) values from each study.


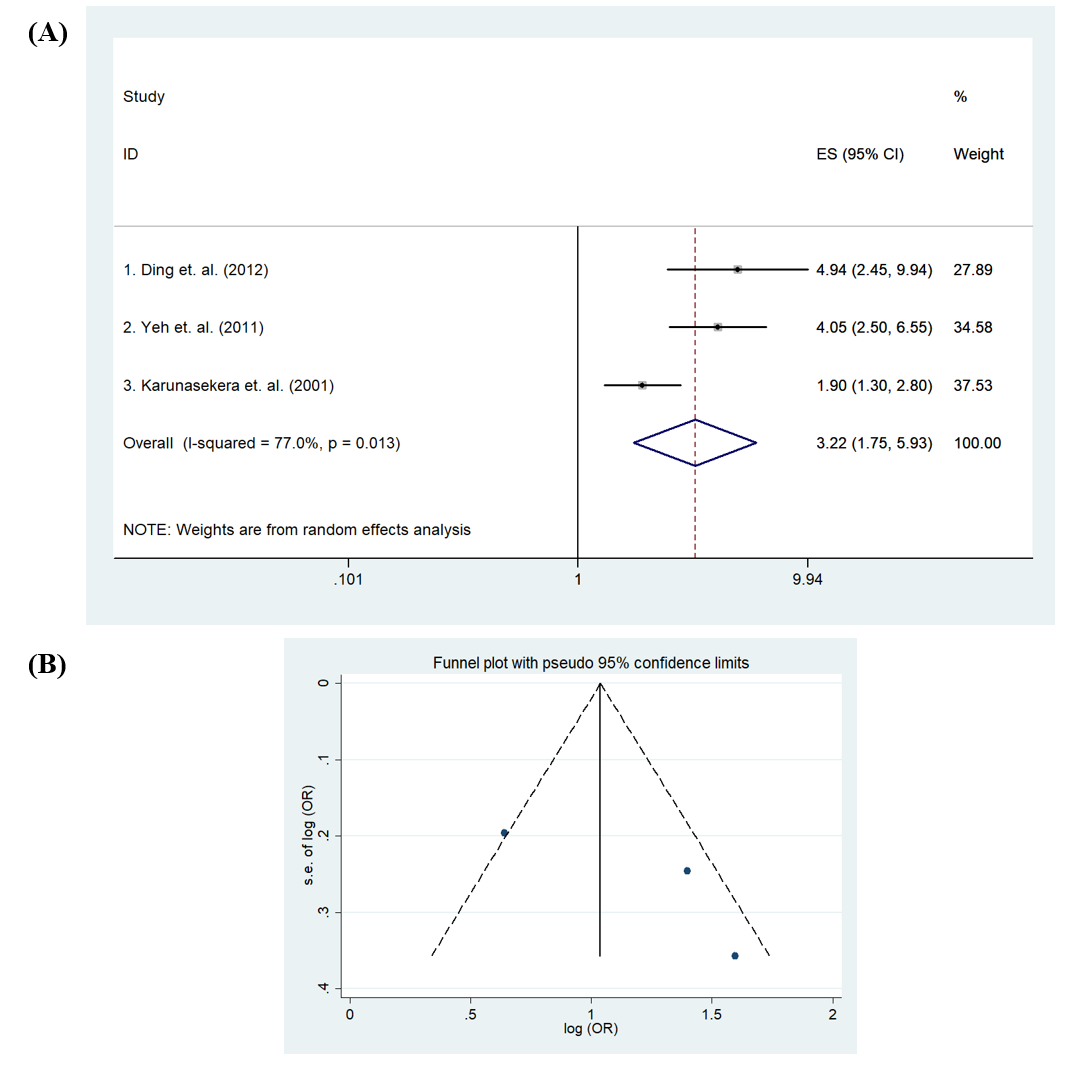


Figure S5. **(A)** Forest Plot and **(B)** Funnel Plot for Random-Effect Meta-Analysis for the Family Medical History of Sibling’s Asthma as Asthma Risk Factor Reported in 3 Independent Studies. Effect size (ES) is represented by the odds ratio (OR) and 95% confidence interval (CI) reported in each study. Results from the heterogeneity test, including the I^2^ value and the heterogeneity p-value (Het P) were also included in the figure. The funnel plot was plotted using the log(OR) and standard error (s.e.) of the log(OR) values from each study.


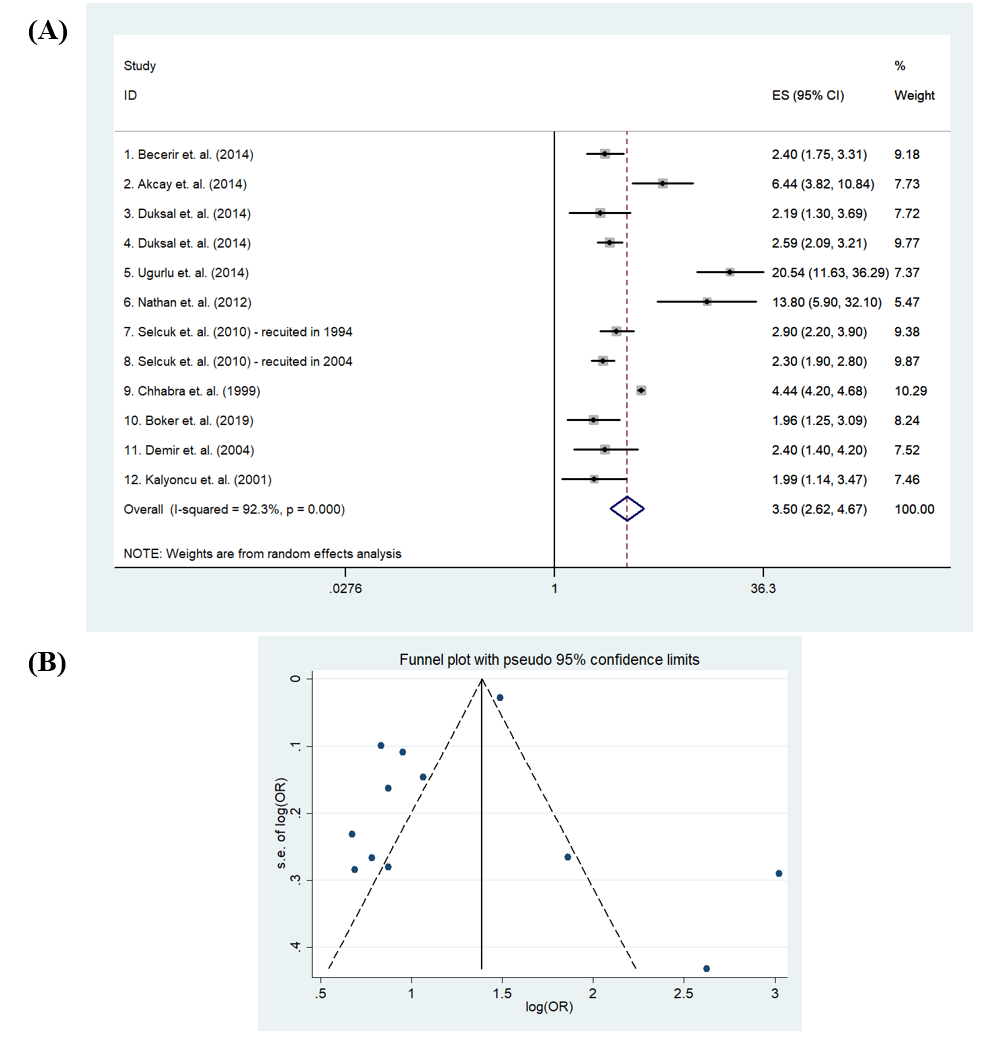


Figure S6. **(A)** Forest Plot and **(B)** Funnel Plot for Random-Effect Meta-Analysis for the Family Medical History of Atopy as Asthma Risk Factor Reported in 11 Independent Studies. Effect size (ES) is represented by the odds ratio (OR) and 95% confidence interval (CI) reported in each study. Results from the heterogeneity test, including the I^2^ value and the heterogeneity p-value (Het P) were also included in the figure. The funnel plot was plotted using the log(OR) and standard error (s.e.) of the log(OR) values from each study.


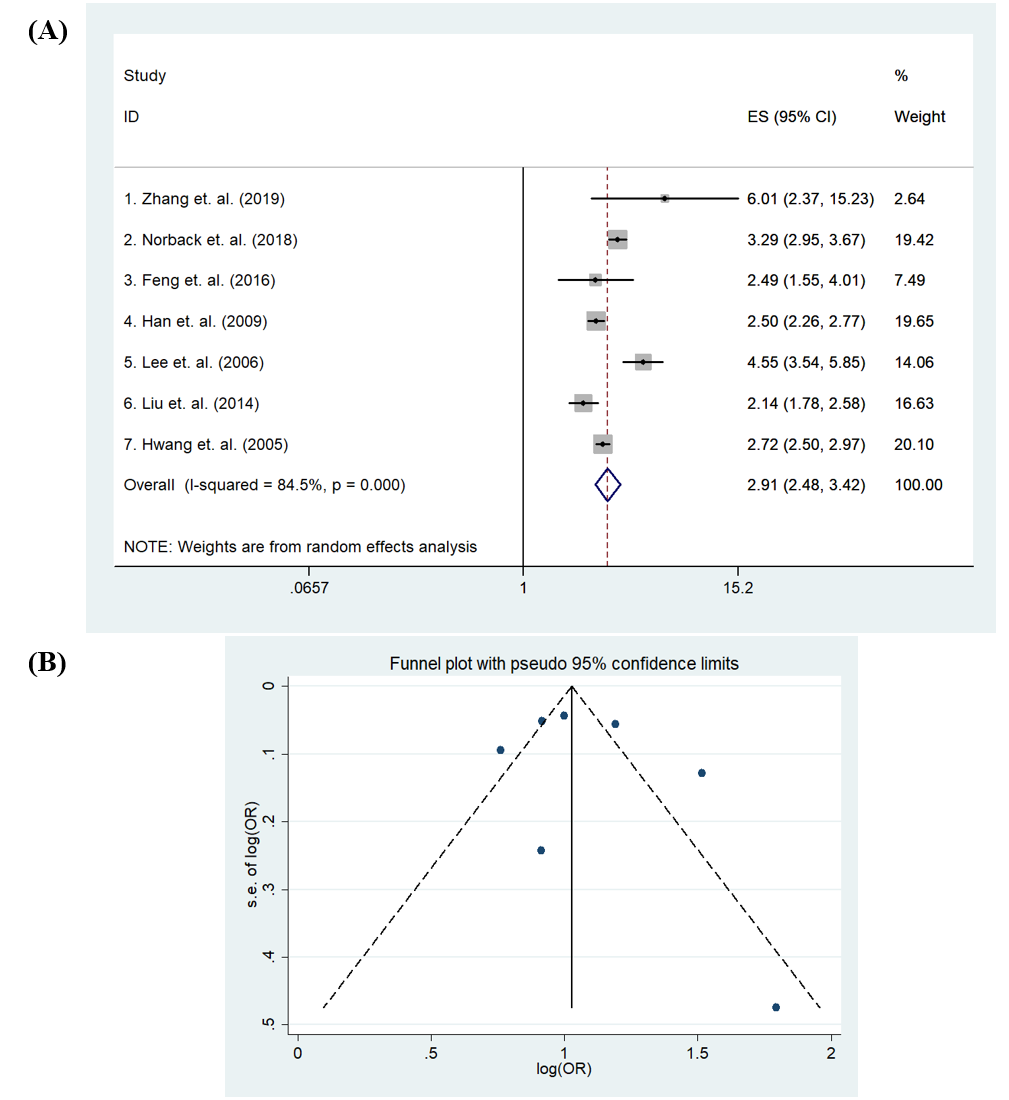


Figure S7. **(A)** Forest Plot and **(B)** Funnel Plot for Random-Effect Meta-Analysis for the Family Medical History of Parental Atopy as Asthma Risk Factor Reported in 7 Independent Studies. Effect size (ES) is represented by the odds ratio (OR) and 95% confidence interval (CI) reported in each study. Results from the heterogeneity test, including the I^2^ value and the heterogeneity p-value (Het P) were also included in the figure. The funnel plot was plotted using the log(OR) and standard error (s.e.) of the log(OR) values from each study.


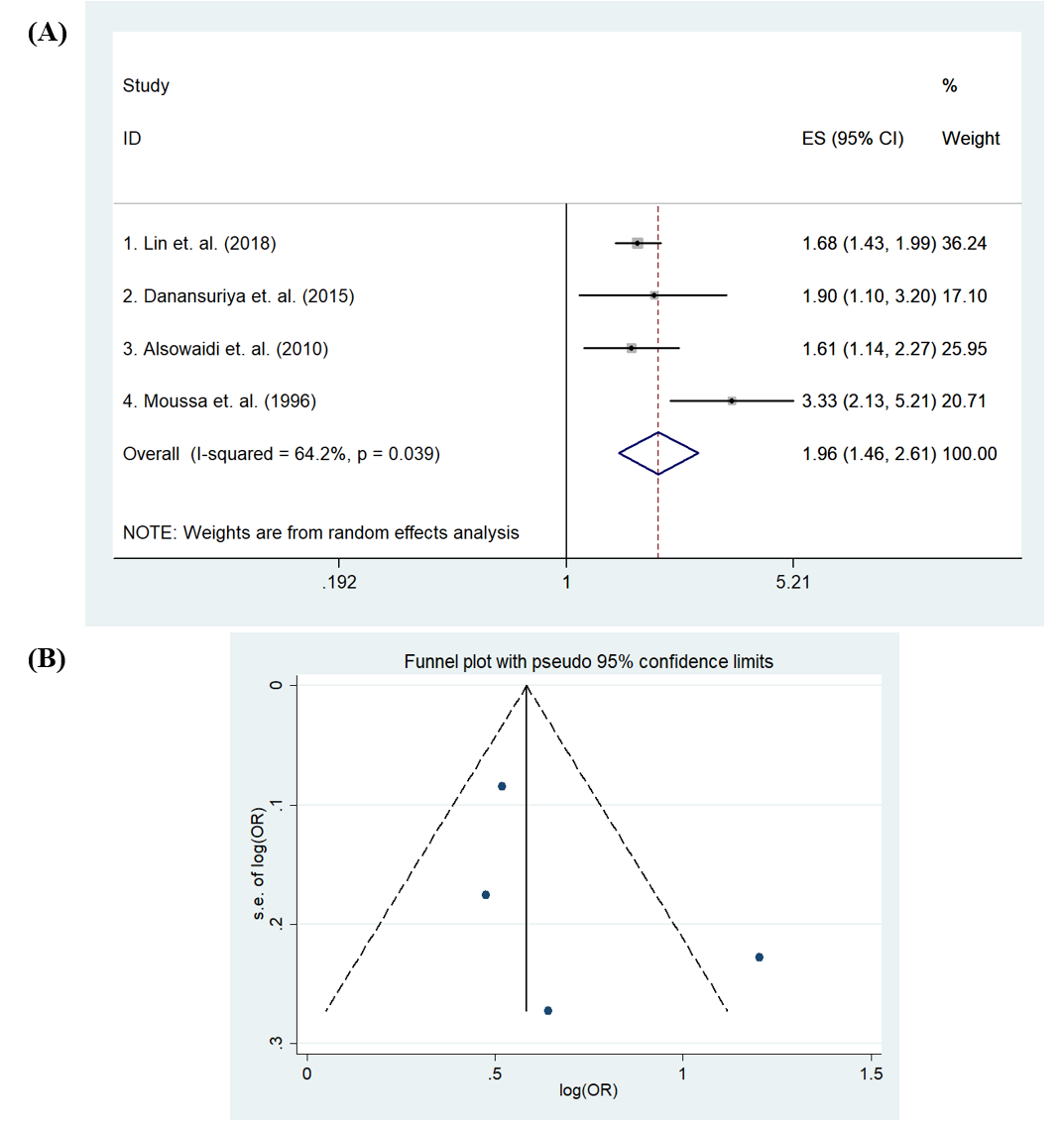


Figure S8. **(A)** Forest Plot and **(B)** Funnel Plot for Random-Effect Meta-Analysis for the Family Medical History of Allergic Rhinitis/Pollinosis as Asthma Risk Factor Reported in 4 Independent Studies. Effect size (ES) is represented by the odds ratio (OR) and 95% confidence interval (CI) reported in each study. Results from the heterogeneity test, including the I^2^ value and the heterogeneity p-value (Het P) were also included in the figure. The funnel plot was plotted using the log(OR) and standard error (s.e.) of the log(OR) values from each study.


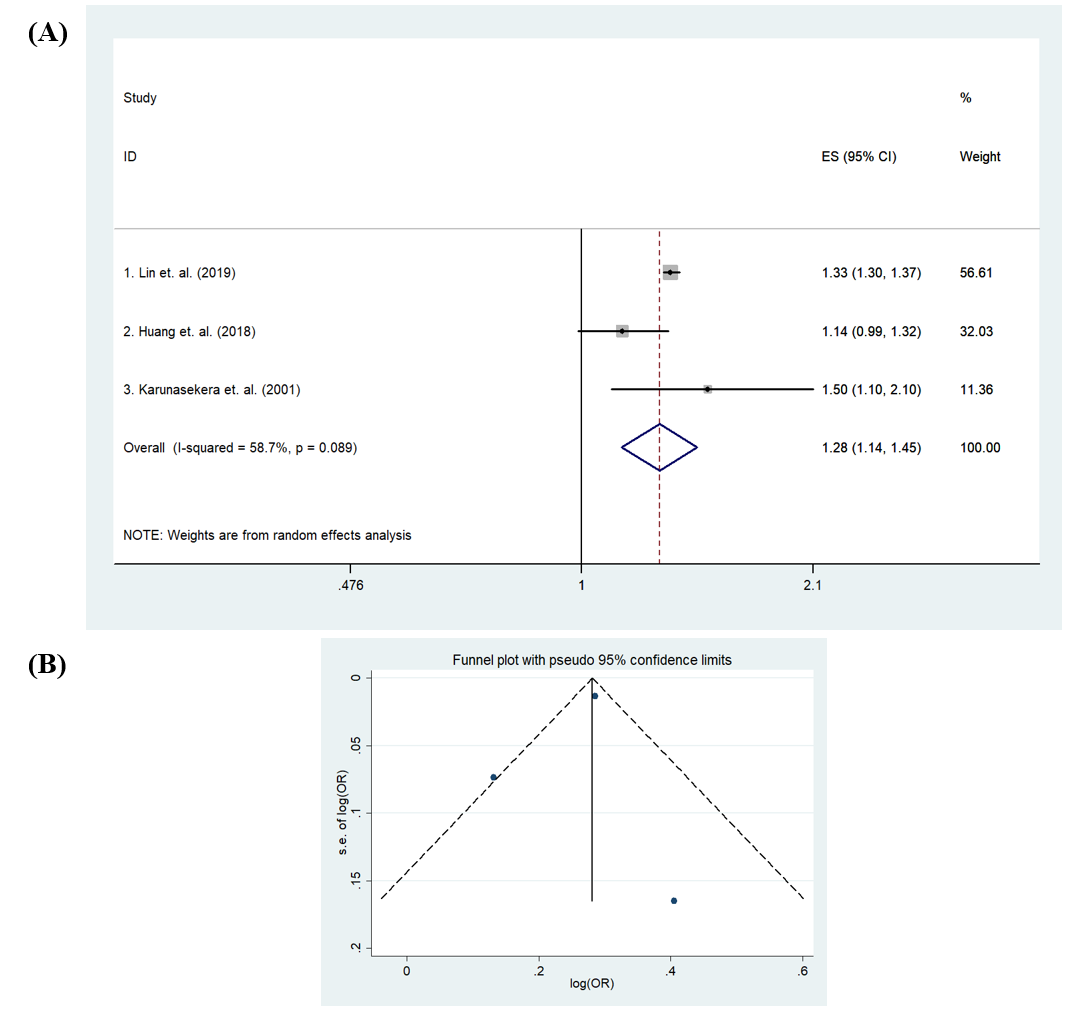


Figure S9. **(A)** Forest Plot and **(B)** Funnel Plot for Random-Effect Meta-Analysis for the Family Medical History of Maternal Allergic Rhinitis/Pollinosis as Asthma Risk Factor Reported in 3 Independent Studies. Effect size (ES) is represented by the odds ratio (OR) and 95% confidence interval (CI) reported in each study. Results from the heterogeneity test, including the I^2^ value and the heterogeneity p-value (Het P) were also included in the figure. The funnel plot was plotted using the log(OR) and standard error (s.e.) of the log(OR) values from each study.


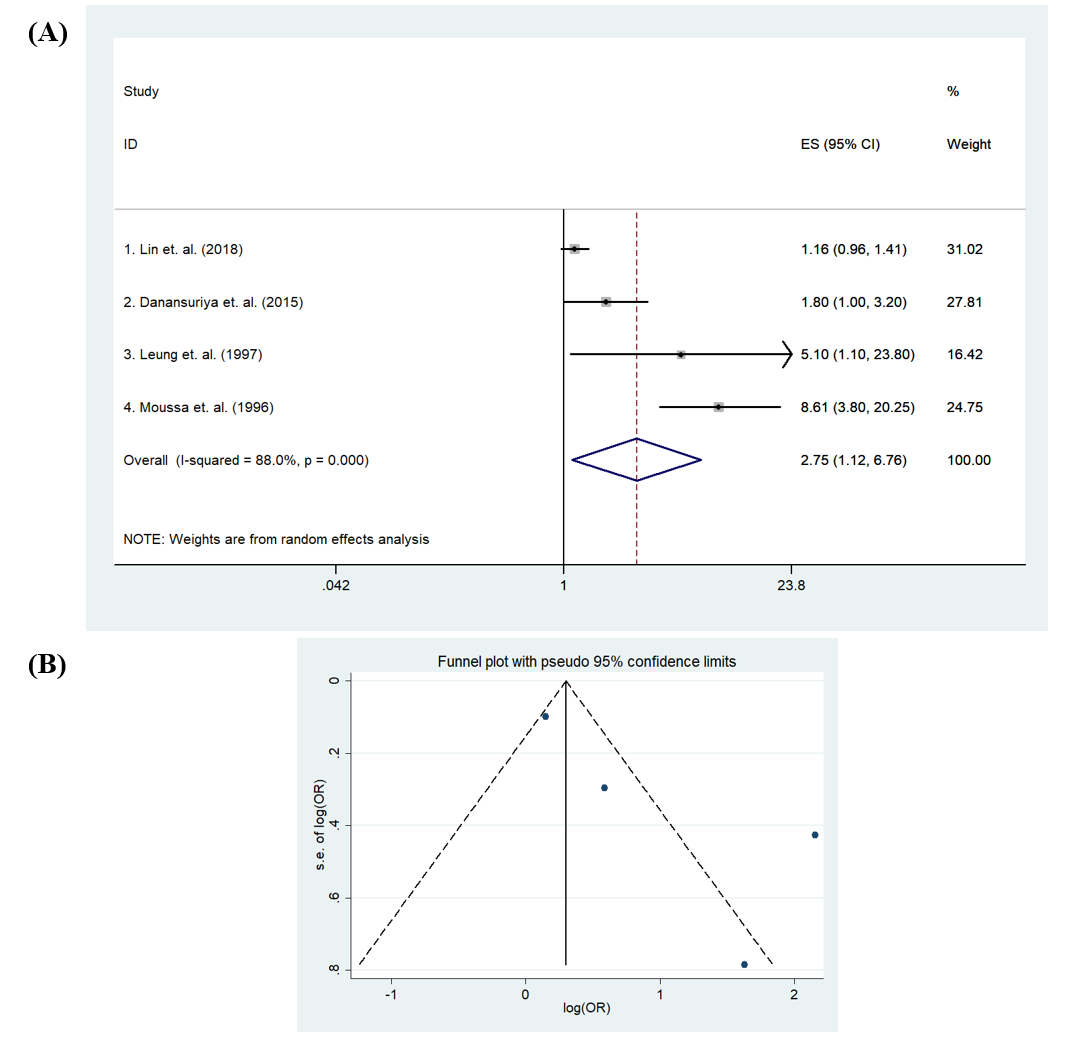


Figure S10. **(A)** Forest Plot and **(B)** Funnel Plot for Random-Effect Meta-Analysis for the Family Medical History of Atopic Dermatitis/Eczema as Asthma Risk Factor Reported in 4 Independent Studies. Effect size (ES) is represented by the odds ratio (OR) and 95% confidence interval (CI) reported in each study. Results from the heterogeneity test, including the I^2^ value and the heterogeneity p-value (Het P) were also included in the figure. The funnel plot was plotted using the log(OR) and standard error (s.e.) of the log(OR) values from each study.


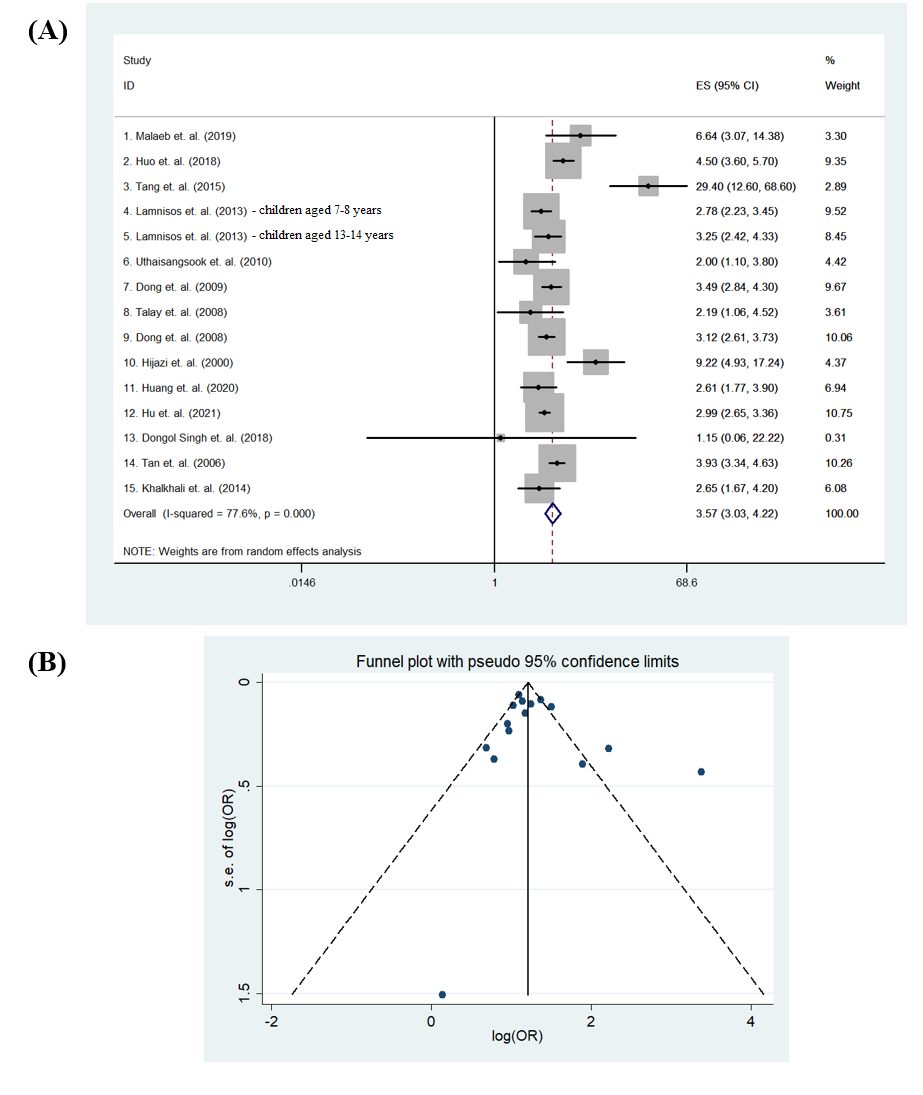


Figure S11. **(A)** Forest Plot and **(B)** Funnel Plot for Random-Effect Meta-Analysis for the Family Medical History of Allergic Diseases as Asthma Risk Factor Reported in 15 Independent Studies. Effect size (ES) is represented by the odds ratio (OR) and 95% confidence interval (CI) reported in each study. Results from the heterogeneity test, including the I^2^ value and the heterogeneity p-value (Het P) were also included in the figure. The funnel plot was plotted using the log(OR) and standard error (s.e.) of the log(OR) values from each study.


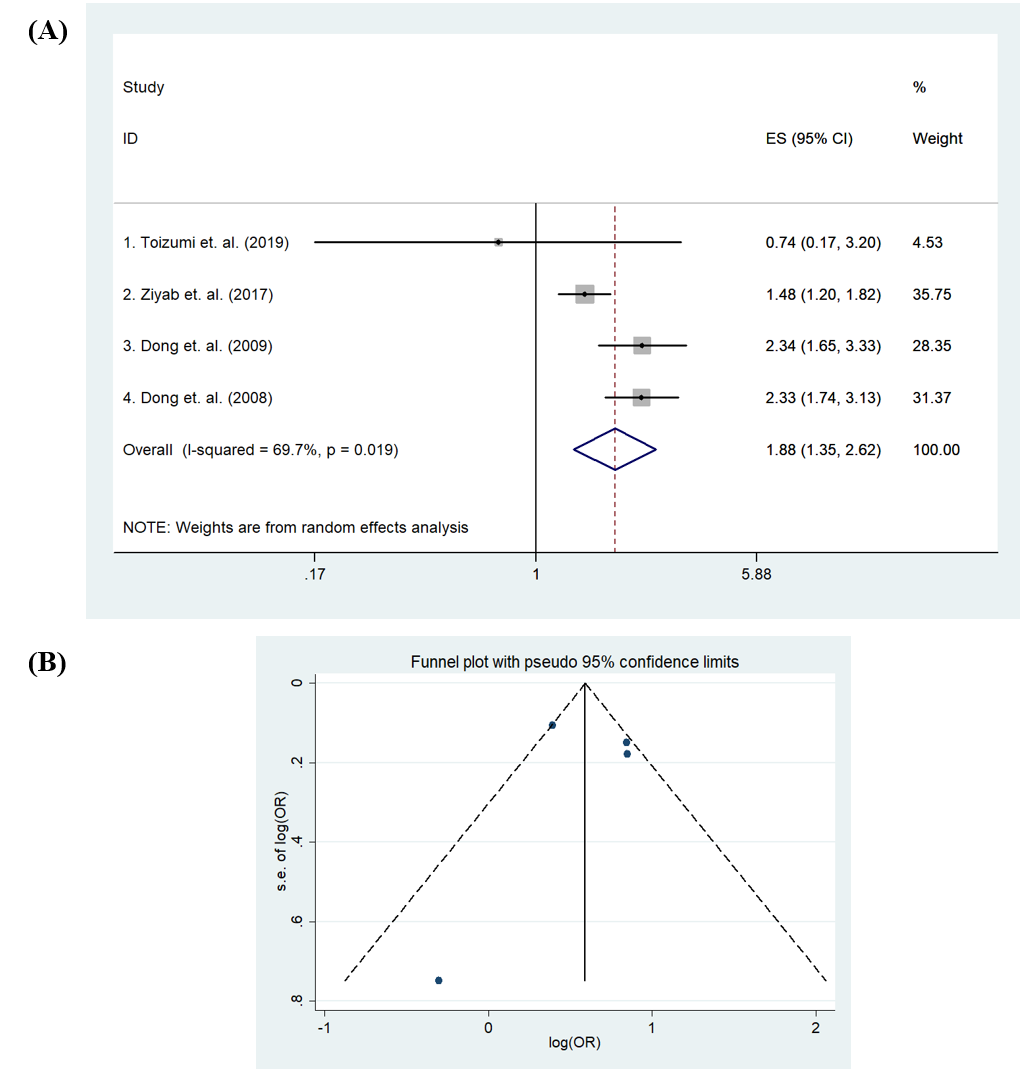


Figure S12. **(A)** Forest Plot and **(B)** Funnel Plot for Random-Effect Meta-Analysis for the Family Medical History of Paternal Allergic Diseases as Asthma Risk Factor Reported in 4 Independent Studies. Effect size (ES) is represented by the odds ratio (OR) and 95% confidence interval (CI) reported in each study. Results from the heterogeneity test, including the I^2^ value and the heterogeneity p-value (Het P) were also included in the figure. The funnel plot was plotted using the log(OR) and standard error (s.e.) of the log(OR) values from each study.


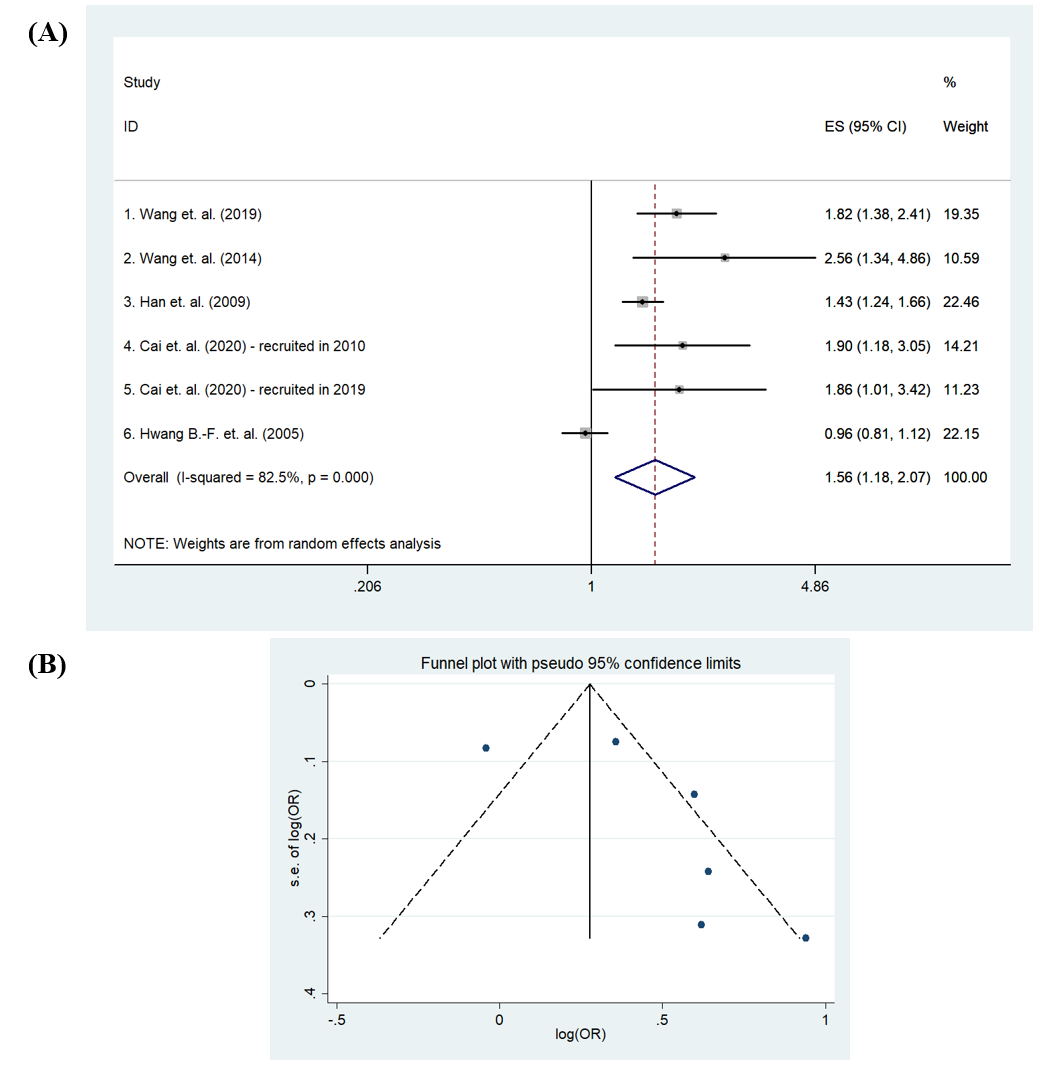


Figure S13. **(A)** Forest Plot and **(B)** Funnel Plot for Random-Effect Meta-Analysis for Presence of Water Damage/Water Leakage in Housing Environment as Asthma Risk Factor Reported in 5 Independent Studies. Effect size (ES) is represented by the odds ratio (OR) and 95% confidence interval (CI) reported in each study. Results from the heterogeneity test, including the I^2^ value and the heterogeneity p-value (Het P) were also included in the figure. The funnel plot was plotted using the log(OR) and standard error (s.e.) of the log(OR) values from each study.


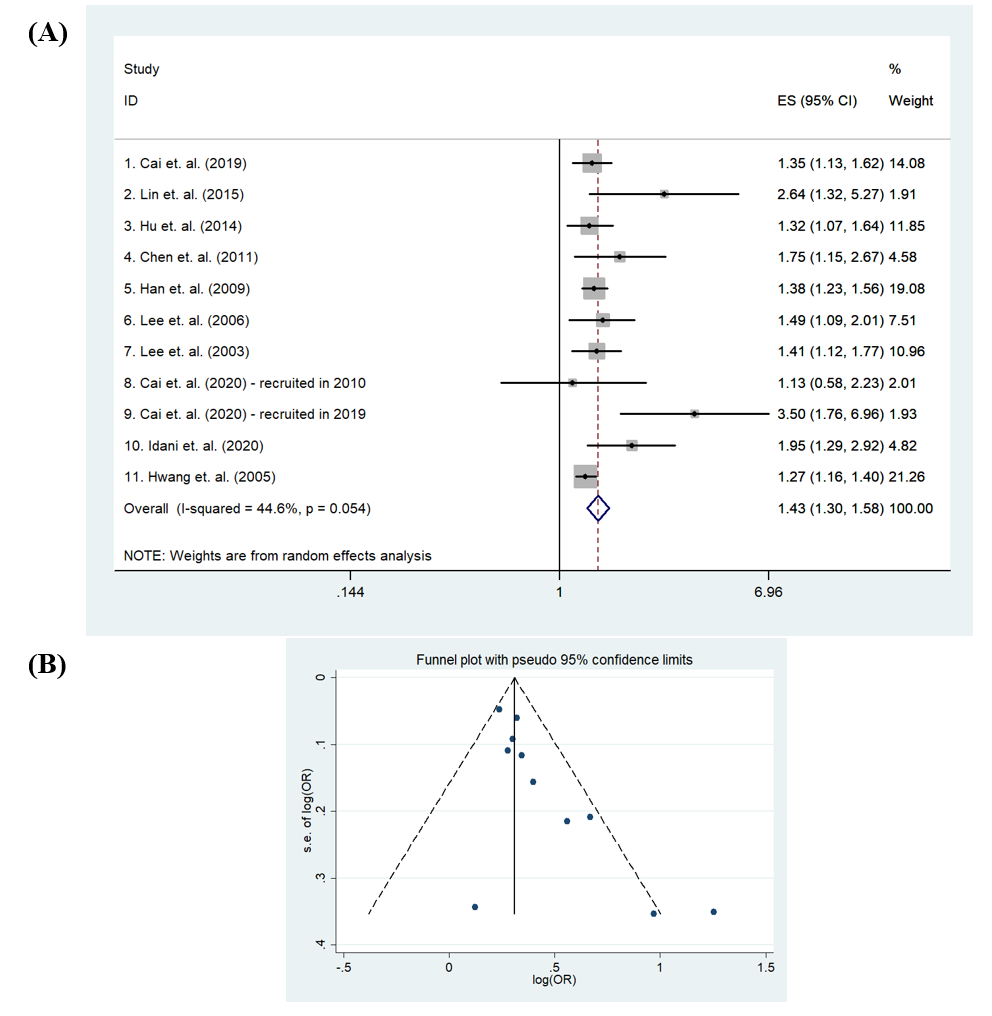


Figure S14. **(A)** Forest Plot and **(B)** Funnel Plot for Random-Effect Meta-Analysis for the Presence of Mold or Mold Spot in Housing Environment as Asthma Risk Factor Reported in 10 Independent Studies. Effect size (ES) is represented by the odds ratio (OR) and 95% confidence interval (CI) reported in each study. Results from the heterogeneity test, including the I^2^ value and the heterogeneity p-value (Het P) were also included in the figure. The funnel plot was plotted using the log(OR) and standard error (s.e.) of the log(OR) values from each study.


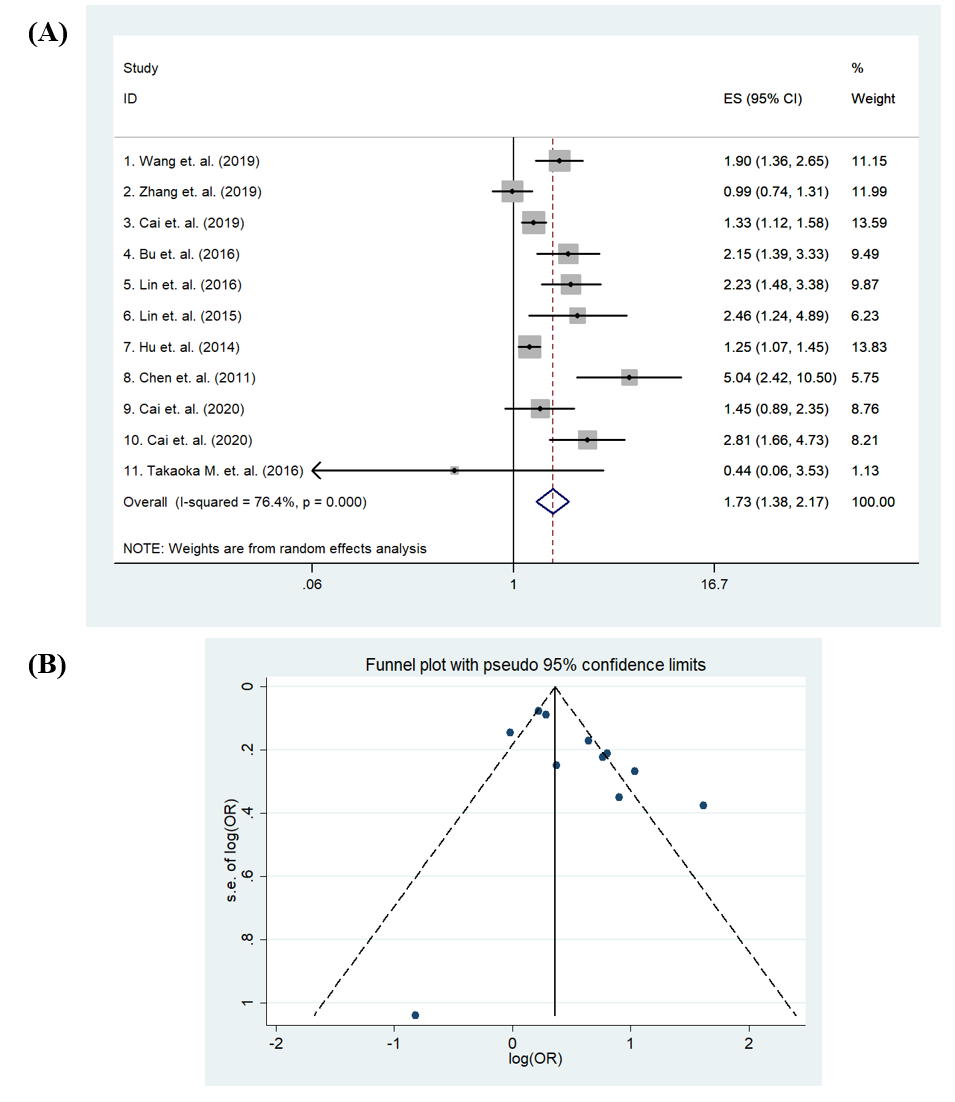


Figure S15. **(A)** Forest Plot and **(B)** Funnel Plot for Random-Effect Meta-Analysis for the Presence of Mold Odor in Housing Environment as Asthma Risk Factor Reported in 10 Independent Studies. Effect size (ES) is represented by the odds ratio (OR) and 95% confidence interval (CI) reported in each study. Results from the heterogeneity test, including the I^2^ value and the heterogeneity p-value (Het P) were also included in the figure. The funnel plot was plotted using the log(OR) and standard error (s.e.) of the log(OR) values from each study.


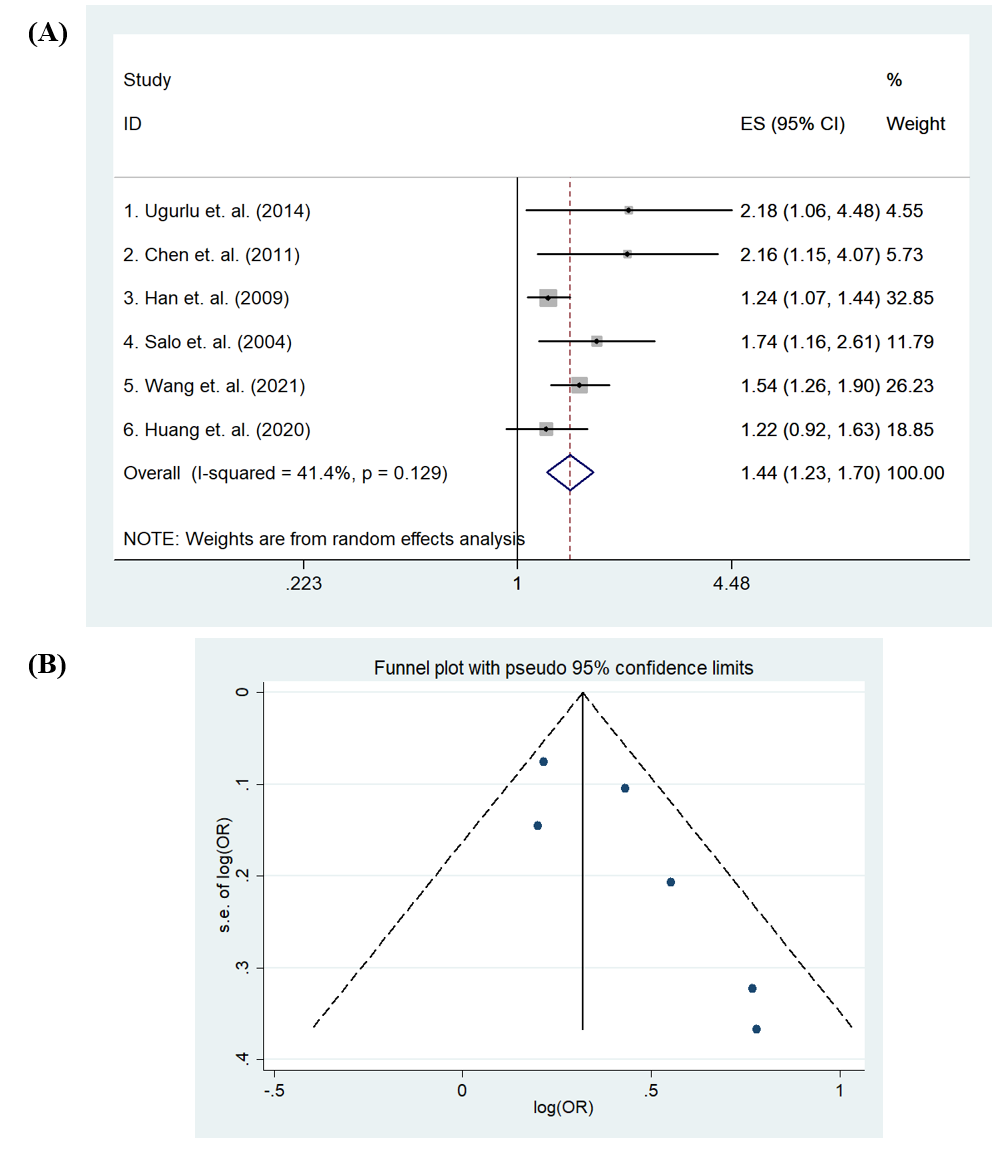


Figure S16. **(A)** Forest Plot and **(B)** Funnel Plot for Random-Effect Meta-Analysis for the Presence of Cockroach in Housing Environment as Asthma Risk Factor Reported in 6 Independent Studies. Effect size (ES) is represented by the odds ratio (OR) and 95% confidence interval (CI) reported in each study. Results from the heterogeneity test, including the I^2^ value and the heterogeneity p-value (Het P) were also included in the figure. The funnel plot was plotted using the log(OR) and standard error (s.e.) of the log(OR) values from each study.


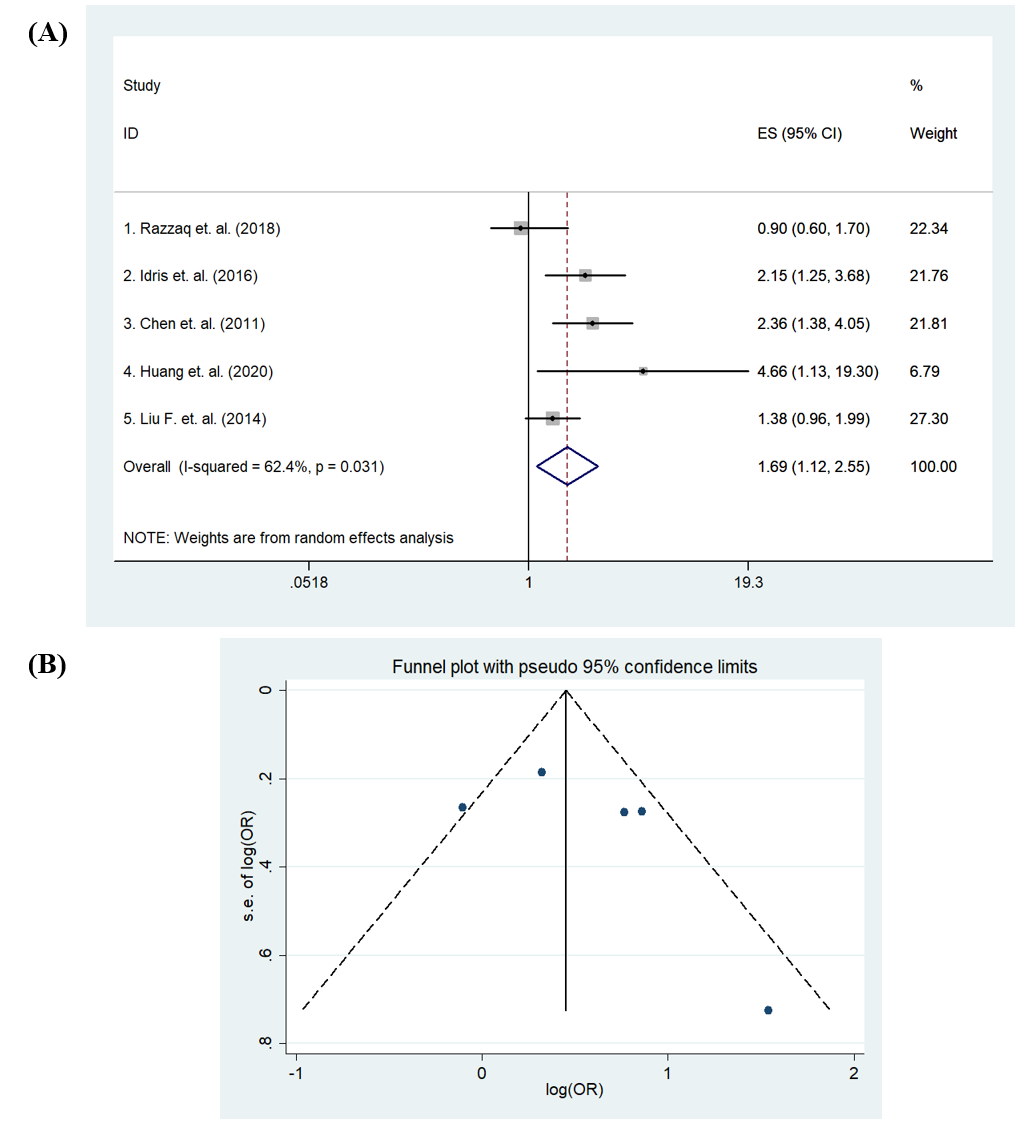


Figure S17. **(A)** Forest Plot and **(B)** Funnel Plot for Random-Effect Meta-Analysis for Carpet Usage in Housing Environment as Asthma Risk Factor Reported in 5 Independent Studies. Effect size (ES) is represented by the odds ratio (OR) and 95% confidence interval (CI) reported in each study. Results from the heterogeneity test, including the I^2^ value and the heterogeneity p-value (Het P) were also included in the figure. The funnel plot was plotted using the log(OR) and standard error (s.e.) of the log(OR) values from each study.


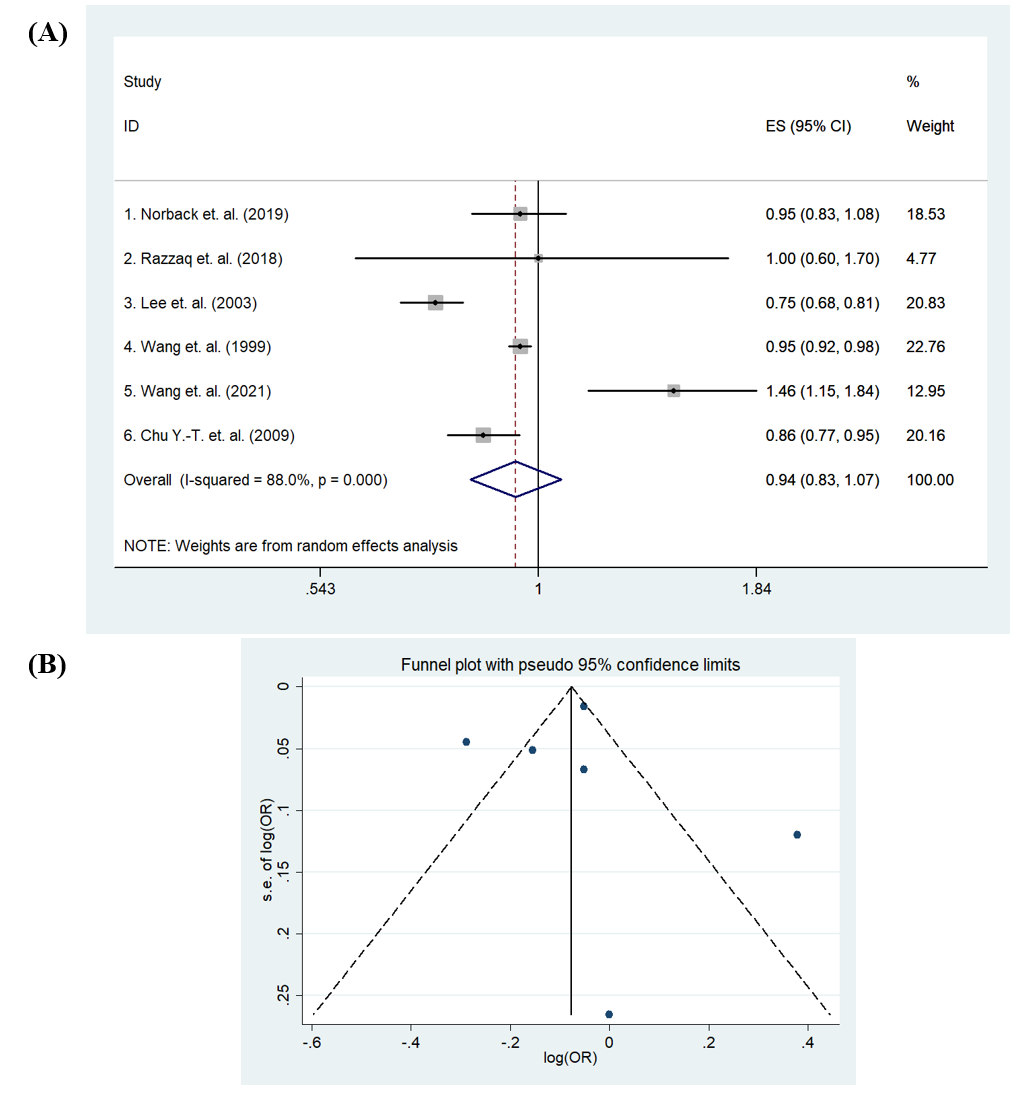


Figure S18. **(A)** Forest Plot and **(B)** Funnel Plot for Random-Effect Meta-Analysis for Incense Burning in Housing Environment as Asthma Risk Factor Reported in 6 Independent Studies. Effect size (ES) is represented by the odds ratio (OR) and 95% confidence interval (CI) reported in each study. Results from the heterogeneity test, including the I^2^ value and the heterogeneity p-value (Het P) were also included in the figure. The funnel plot was plotted using the log(OR) and standard error (s.e.) of the log(OR) values from each study.


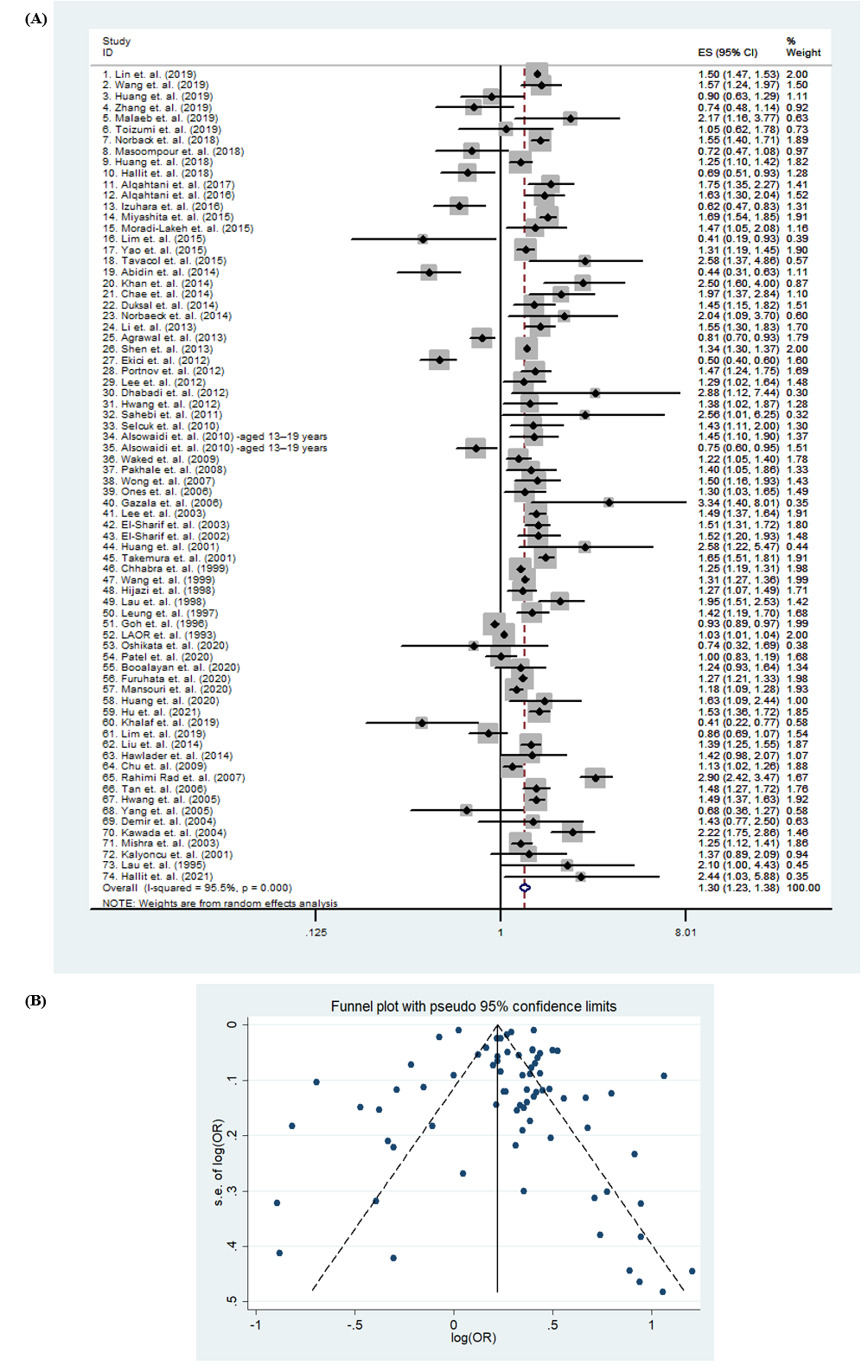


Figure S19. **(A)** Forest Plot and **(B)** Funnel Plot for Random-Effect Meta-Analysis for Gender as Asthma Risk Factor Reported in 73 Independent Studies. Effect size (ES) is represented by the odds ratio (OR) and 95% confidence interval (CI) reported in each study. Results from the heterogeneity test, including the I^2^ value and the heterogeneity p-value (Het P) were also included in the figure. The funnel plot was plotted using the log(OR) and standard error (s.e.) of the log(OR) values from each study.


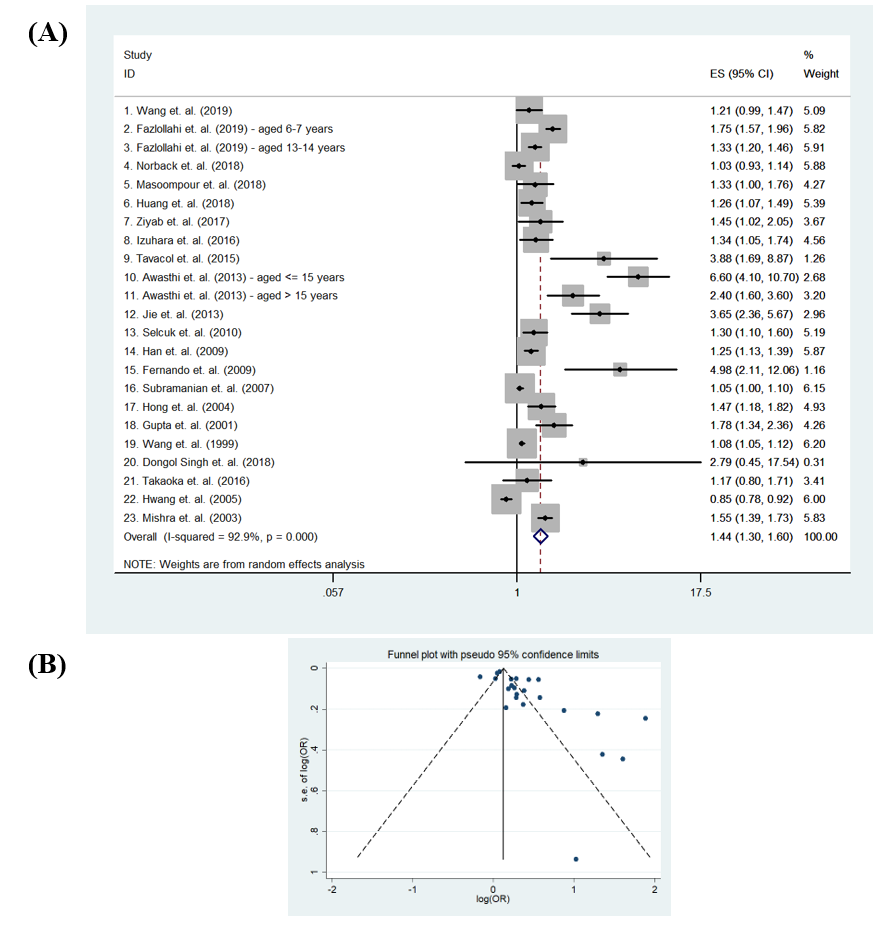


Figure S20. **(A)** Forest Plot and **(B)** Funnel Plot for Random-Effect Meta-Analysis for Cigarette Smoke Exposure as Asthma Risk Factor Reported in 21 Independent Studies. Effect size (ES) is represented by the odds ratio (OR) and 95% confidence interval (CI) reported in each study. Results from the heterogeneity test, including the I^2^ value and the heterogeneity p-value (Het P) were also included in the figure. The funnel plot was plotted using the log(OR) and standard error (s.e.) of the log(OR) values from each study.


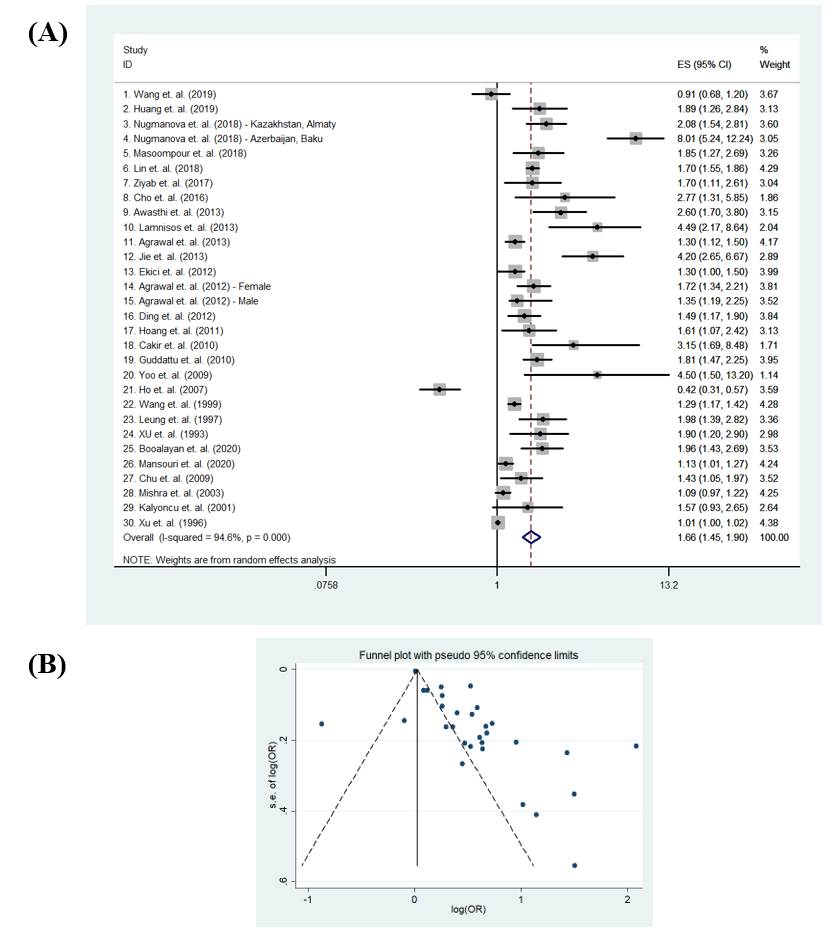


Figure S21. **(A)** Forest Plot and **(B)** Funnel Plot for Random-Effect Meta-Analysis for Cigarette Smoking as Asthma Risk Factor Reported in 21 Independent Studies. Effect size (ES) is represented by the odds ratio (OR) and 95% confidence interval (CI) reported in each study. Results from the heterogeneity test, including the I^2^ value and the heterogeneity p-value (Het P) were also included in the figure. The funnel plot was plotted using the log(OR) and standard error (s.e.) of the log(OR) values from each study.


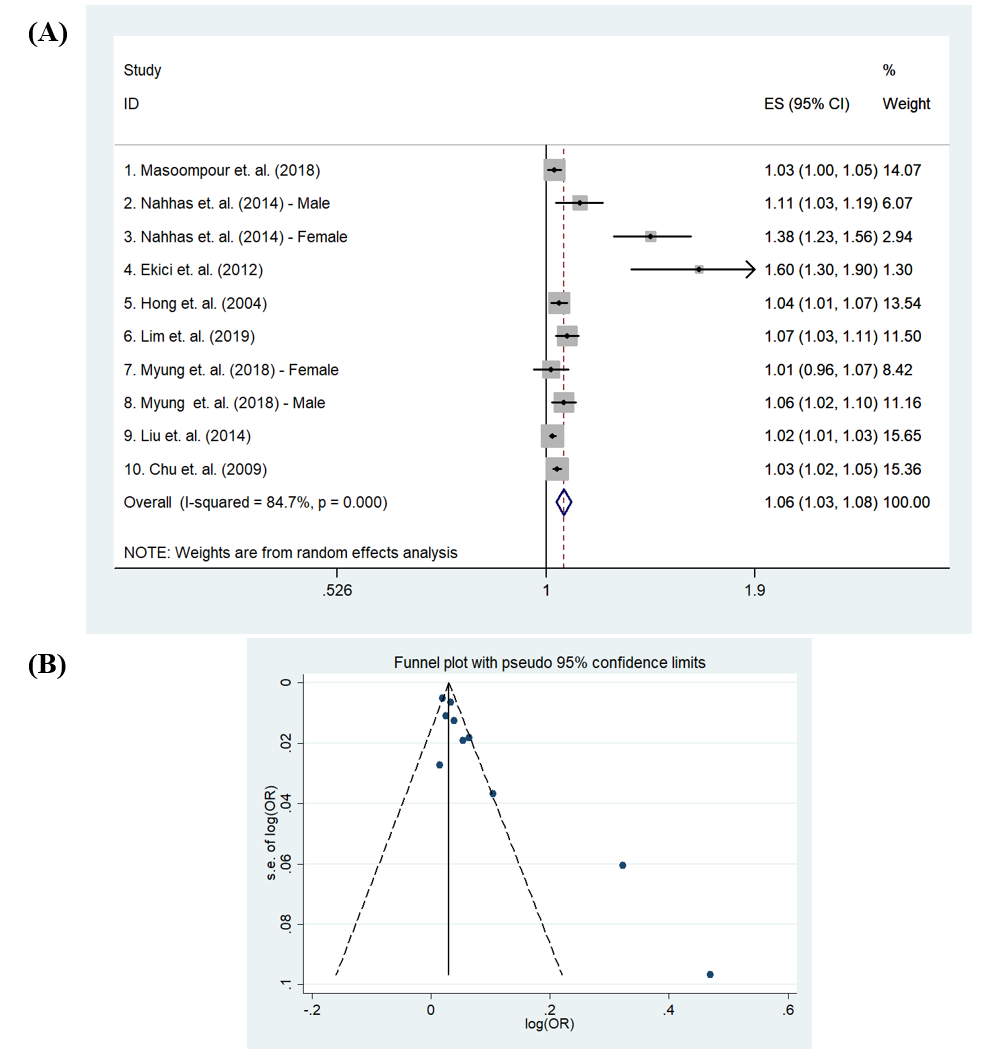


Figure S22. **(A)** Forest Plot and **(B)** Funnel Plot for Random-Effect Meta-Analysis for Body Mass Index (BMI, kg/m^2^, continuous variable) as Asthma Risk Factor Reported in 8 Independent Studies. Effect size (ES) is represented by the odds ratio (OR) and 95% confidence interval (CI) reported in each study. Results from the heterogeneity test, including the I^2^ value and the heterogeneity p-value (Het P) were also included in the figure. The funnel plot was plotted using the log(OR) and standard error (s.e.) of the log(OR) values from each study.


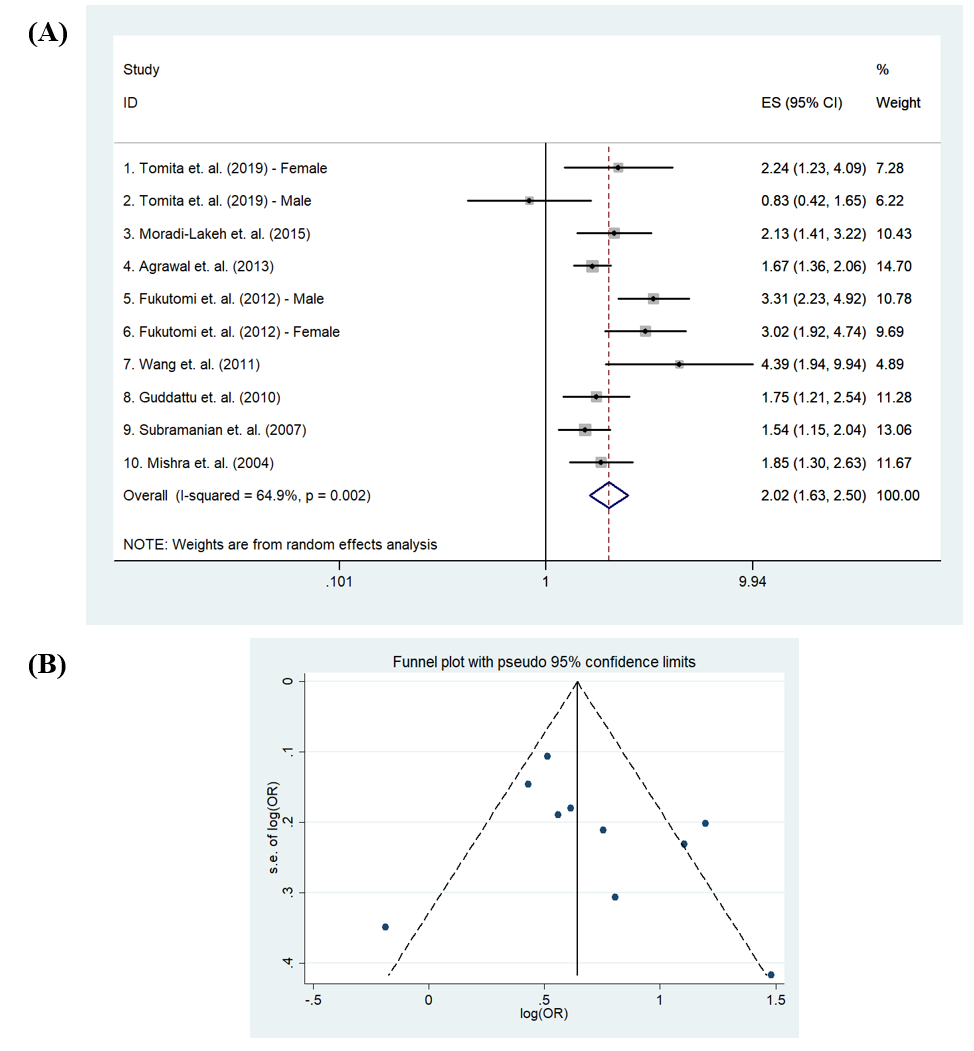


Figure S23. **(A)** Forest Plot and **(B)** Funnel Plot for Random-Effect Meta-Analysis for Obese (Body Mass Index, BMI > 30kg/m^2^) as Asthma Risk Factor Reported in 8 Independent Studies. Effect size (ES) is represented by the odds ratio (OR) and 95% confidence interval (CI) reported in each study. Results from the heterogeneity test, including the I^2^ value and the heterogeneity p-value (Het P) were also included in the figure. The funnel plot was plotted using the log(OR) and standard error (s.e.) of the log(OR) values from each study.


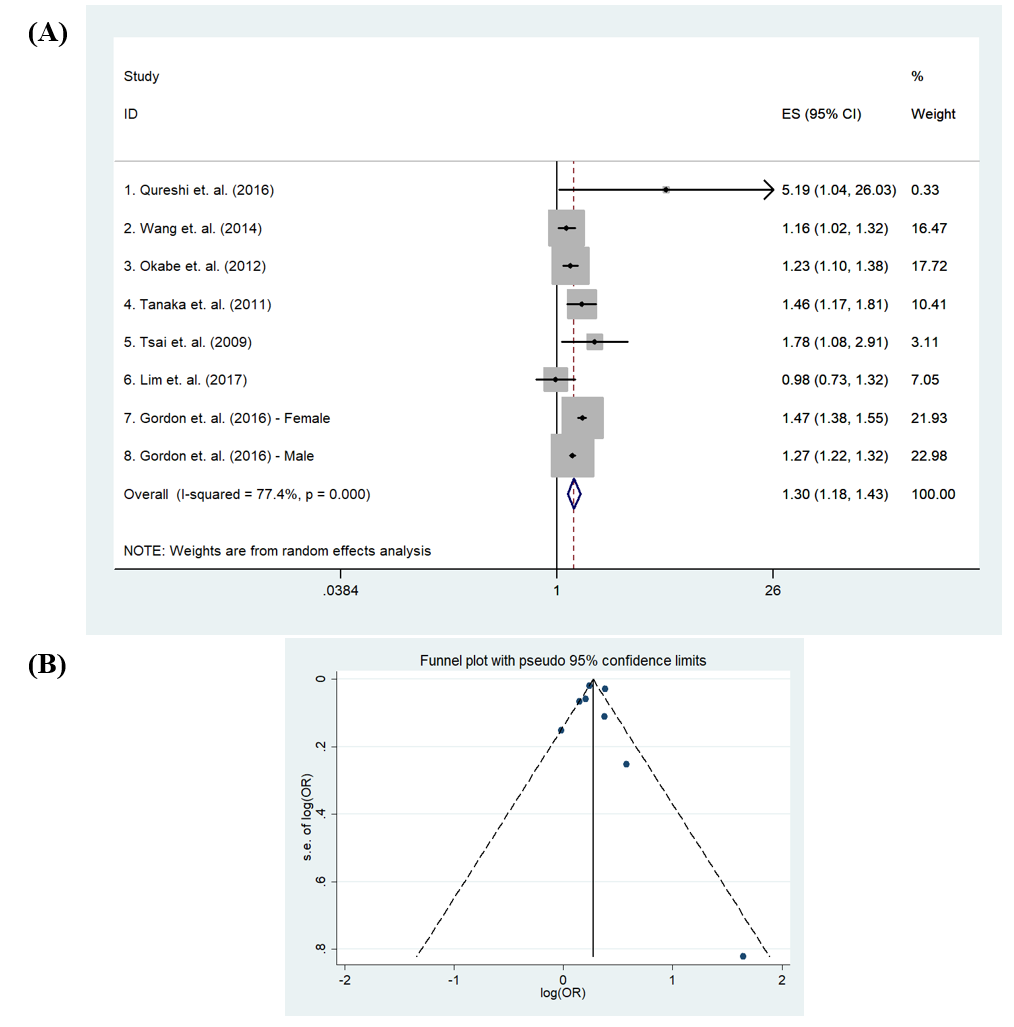


Figure S24. **(A)** Forest Plot and **(B)** Funnel Plot for Random-Effect Meta-Analysis for Obese (Body Mass Index, BMI > 95 percentile) as Asthma Risk Factor Reported in 7 Independent Studies. Effect size (ES) is represented by the odds ratio (OR) and 95% confidence interval (CI) reported in each study. Results from the heterogeneity test, including the I^2^ value and the heterogeneity p-value (Het P) were also included in the figure. The funnel plot was plotted using the log(OR) and standard error (s.e.) of the log(OR) values from each study.


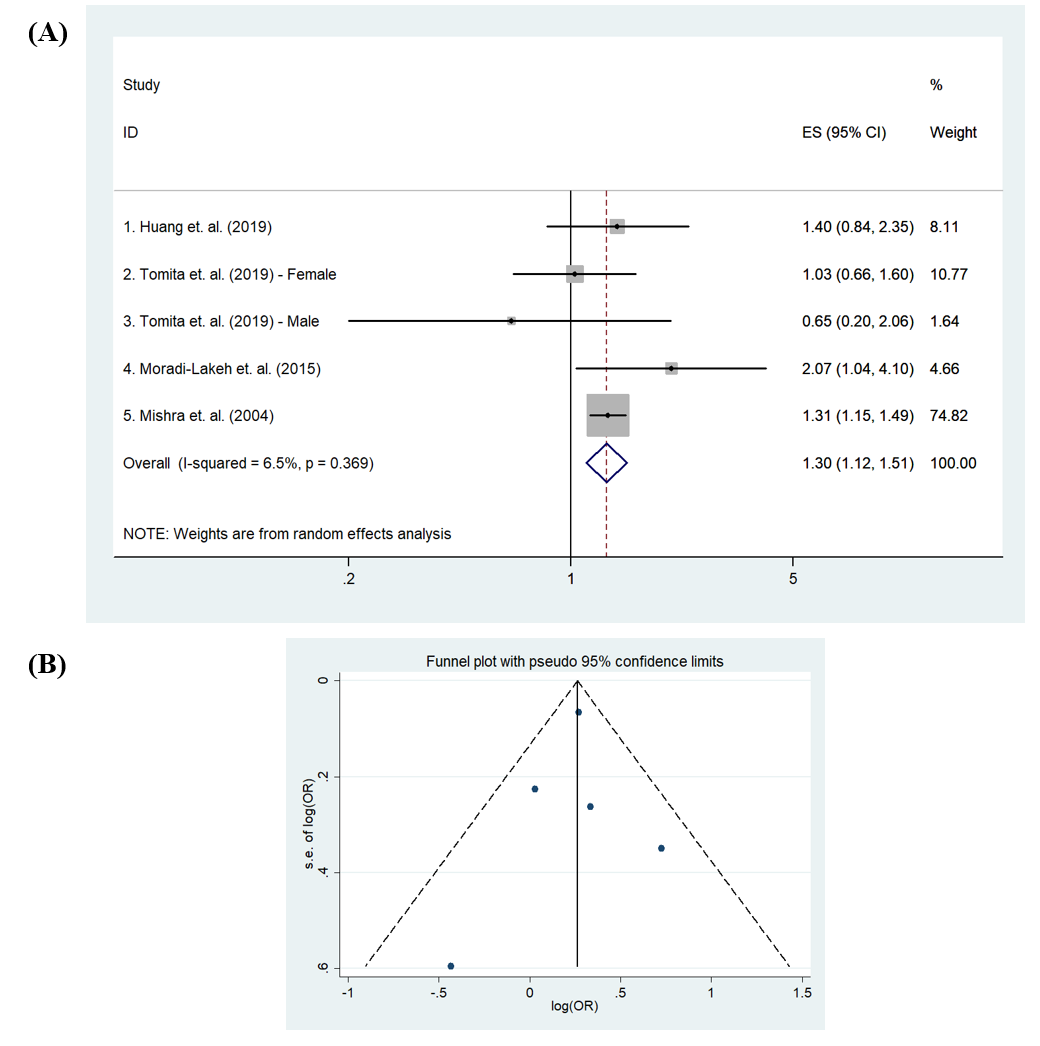


Figure S25. **(A)** Forest Plot and **(B)** Funnel Plot for Random-Effect Meta-Analysis for Underweight (Body Mass Index, BMI < 18.5kg/m^2^) as Asthma Risk Factor Reported in 4 Independent Studies. Effect size (ES) is represented by the odds ratio (OR) and 95% confidence interval (CI) reported in each study. Results from the heterogeneity test, including the I^2^ value and the heterogeneity p-value (Het P) were also included in the figure. The funnel plot was plotted using the log(OR) and standard error (s.e.) of the log(OR) values from each study.


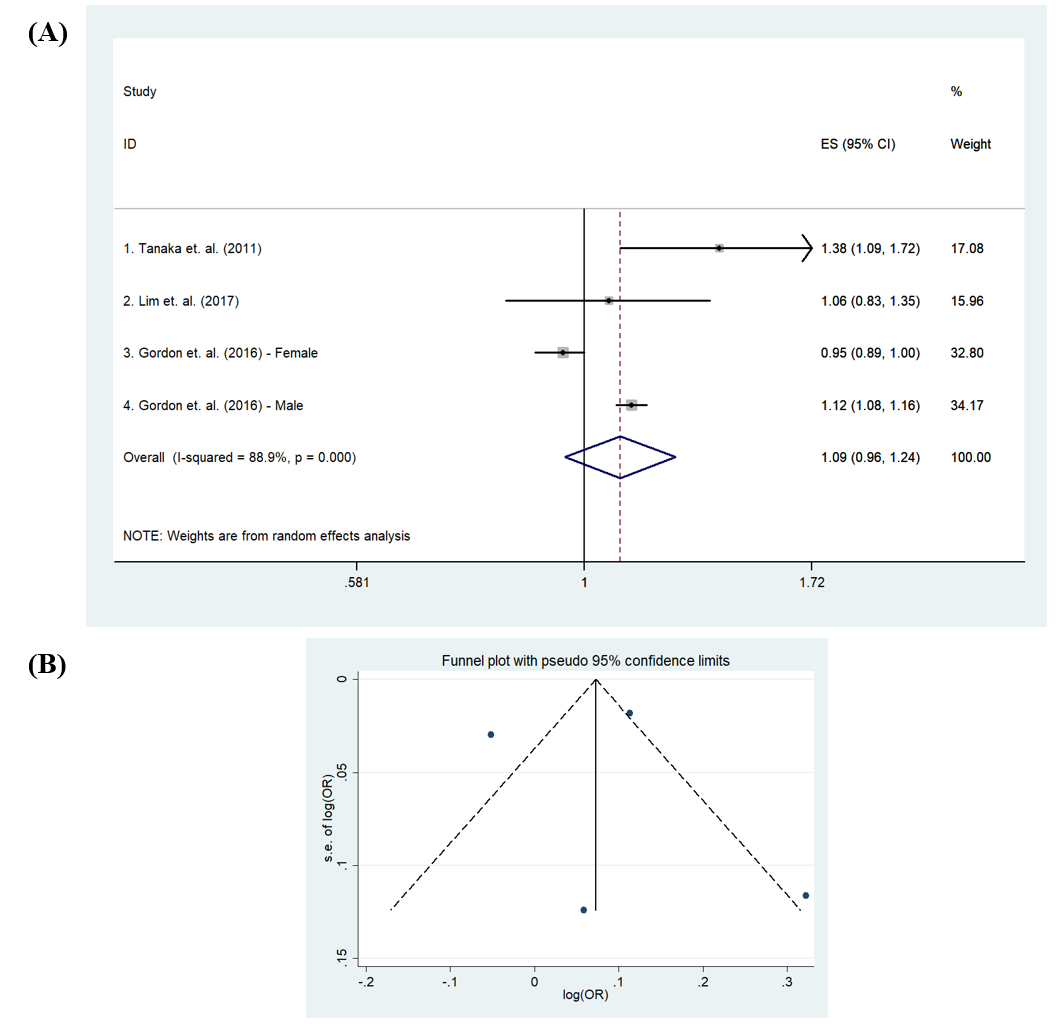


Figure S26. **(A)** Forest Plot and **(B)** Funnel Plot for Random-Effect Meta-Analysis for Underweight (Body Mass Index, BMI < 5 percentile) as Asthma Risk Factor Reported in 4 Independent Studies. Effect size (ES) is represented by the odds ratio (OR) and 95% confidence interval (CI) reported in each study. Results from the heterogeneity test, including the I^2^ value and the heterogeneity p-value (Het P) were also included in the figure. The funnel plot was plotted using the log(OR) and standard error (s.e.) of the log(OR) values from each study.


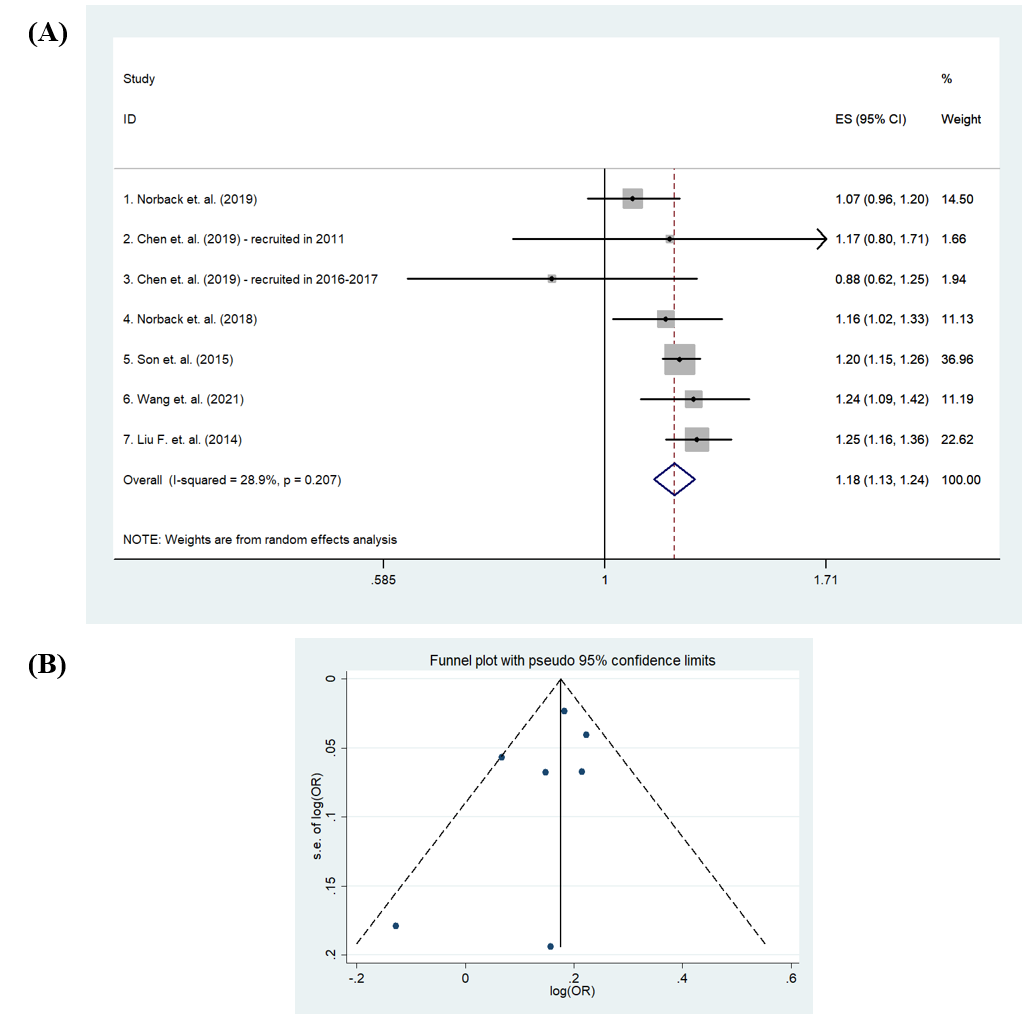


Figure S27. **(A)** Forest Plot and **(B)** Funnel Plot for Random-Effect Meta-Analysis for Air Pollution (NO_2_) as Asthma Risk Factor Reported in 6 Independent Studies. Effect size (ES) is represented by the odds ratio (OR) and 95% confidence interval (CI) reported in each study. Results from the heterogeneity test, including the I^2^ value and the heterogeneity p-value (Het P) were also included in the figure. The funnel plot was plotted using the log(OR) and standard error (s.e.) of the log(OR) values from each study.


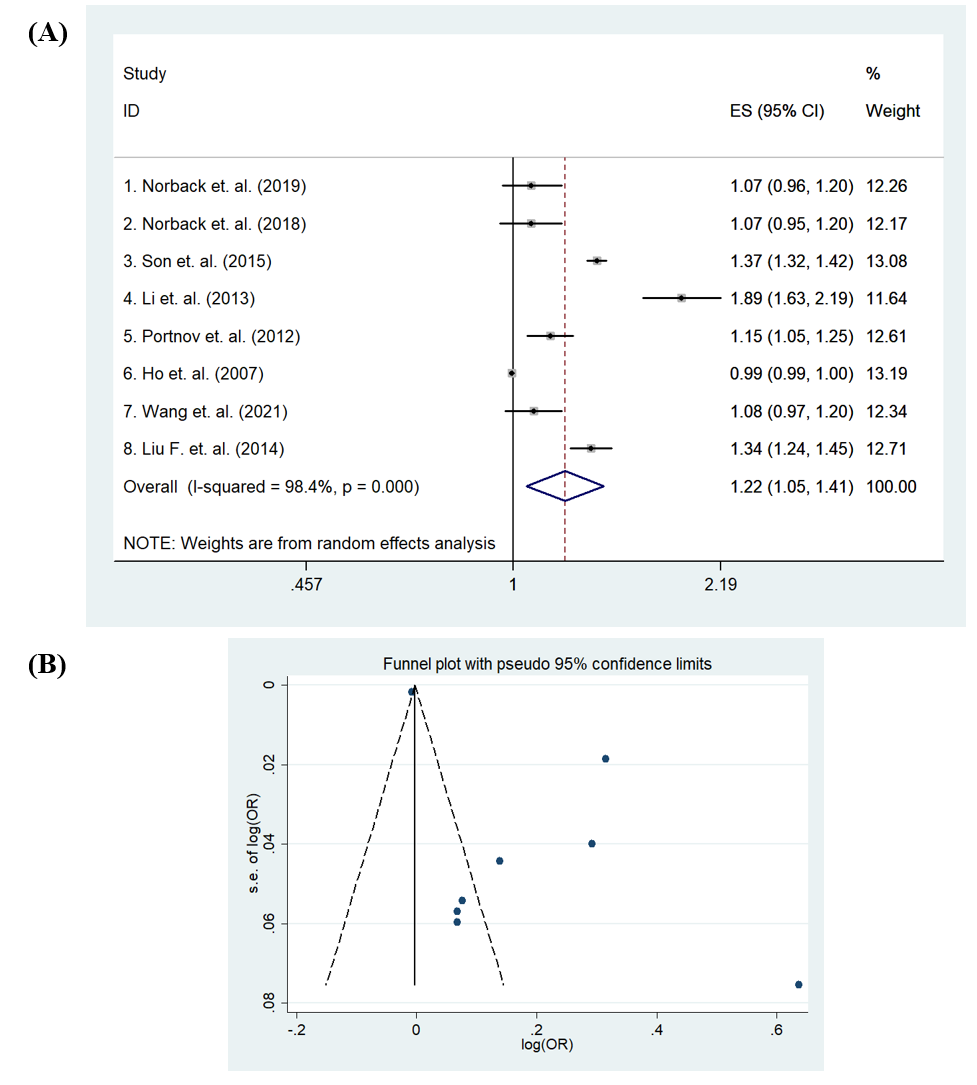


Figure S28. **(A)** Forest Plot and **(B)** Funnel Plot for Random-Effect Meta-Analysis for Air Pollution (PM10) as Asthma Risk Factor Reported in 8 Independent Studies. Effect size (ES) is represented by the odds ratio (OR) and 95% confidence interval (CI) reported in each study. Results from the heterogeneity test, including the I^2^ value and the heterogeneity p-value (Het P) were also included in the figure. The funnel plot was plotted using the log(OR) and standard error (s.e.) of the log(OR) values from each study.


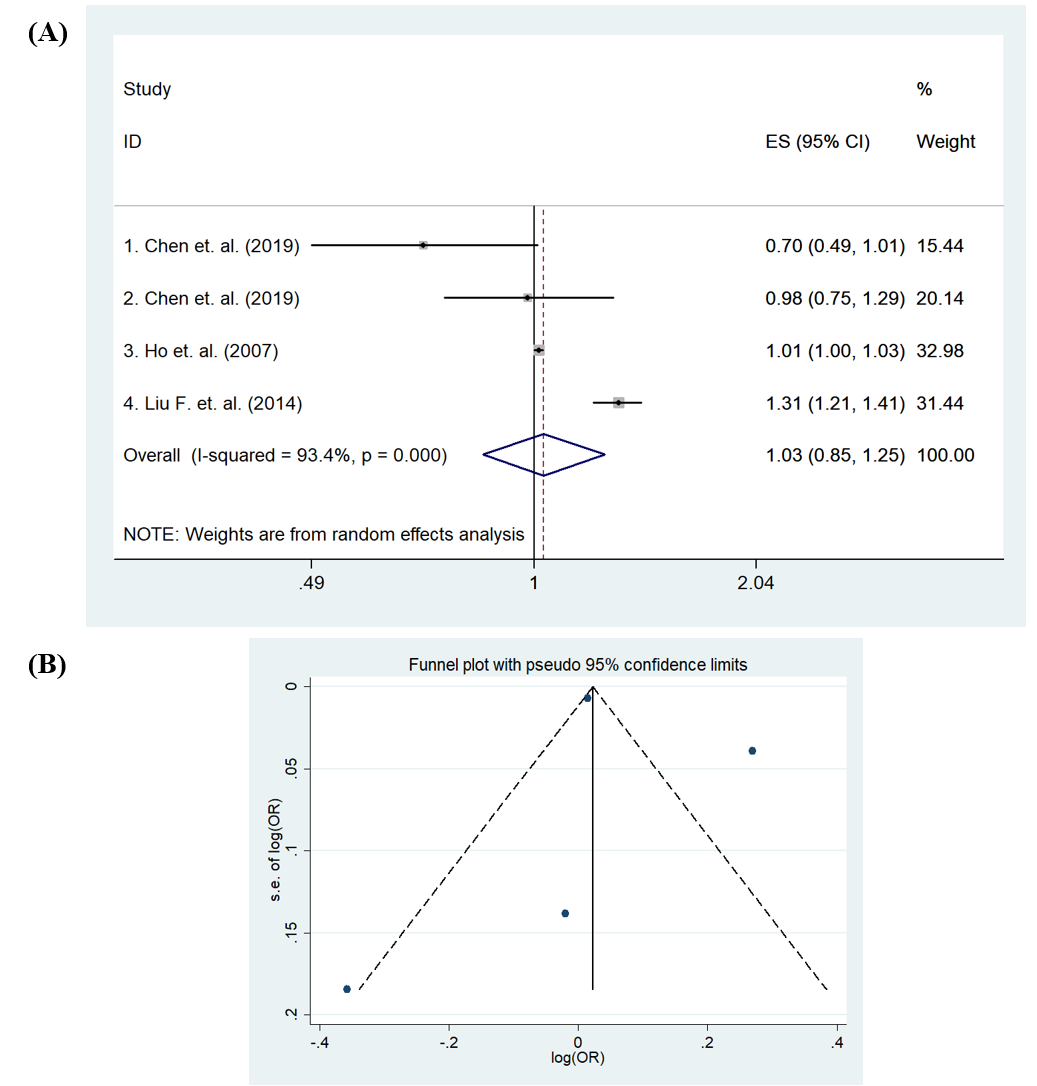


Figure S29. **(A)** Forest Plot and **(B)** Funnel Plot for Random-Effect Meta-Analysis for Air Pollution (O_3_) as Asthma Risk Factor Reported in 6 Independent Studies. Effect size (ES) is represented by the odds ratio (OR) and 95% confidence interval (CI) reported in each study. Results from the heterogeneity test, including the I^2^ value and the heterogeneity p-value (Het P) were also included in the figure. The funnel plot was plotted using the log(OR) and standard error (s.e.) of the log(OR) values from each study.


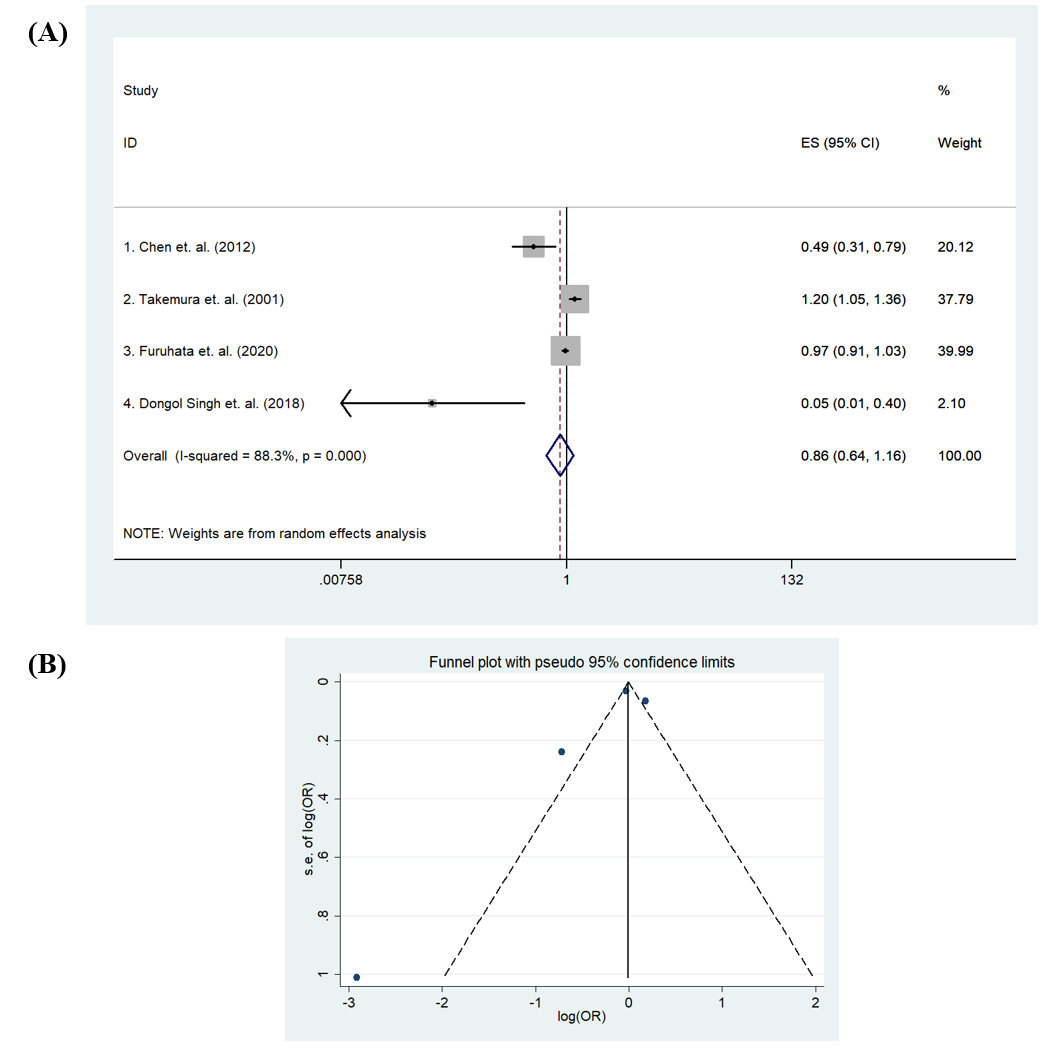


Figure S30. **(A)** Forest Plot and **(B)** Funnel Plot for Random-Effect Meta-Analysis for Exclusive Breastfeeding as Asthma Risk Factor Reported in 4 Independent Studies. Effect size (ES) is represented by the odds ratio (OR) and 95% confidence interval (CI) reported in each study. Results from the heterogeneity test, including the I^2^ value and the heterogeneity p-value (Het P) were also included in the figure. The funnel plot was plotted using the log(OR) and standard error (s.e.) of the log(OR) values from each study.


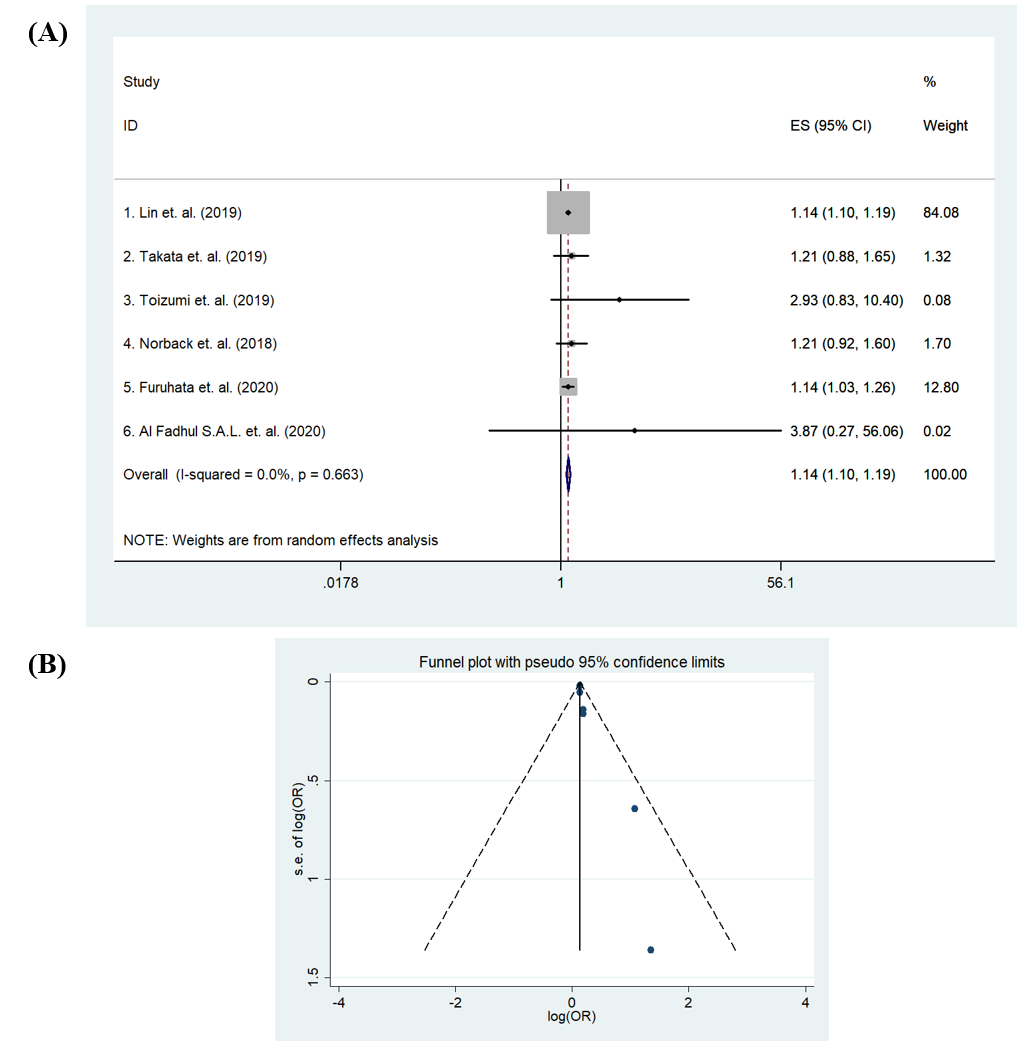


Figure S31. **(A)** Forest Plot and **(B)** Funnel Plot for Random-Effect Meta-Analysis for Low Birth Weight (< 2500g) as Asthma Risk Factor Reported in 6 Independent Studies. Effect size (ES) is represented by the odds ratio (OR) and 95% confidence interval (CI) reported in each study. Results from the heterogeneity test, including the I^2^ value and the heterogeneity p-value (Het P) were also included in the figure. The funnel plot was plotted using the log(OR) and standard error (s.e.) of the log(OR) values from each study.


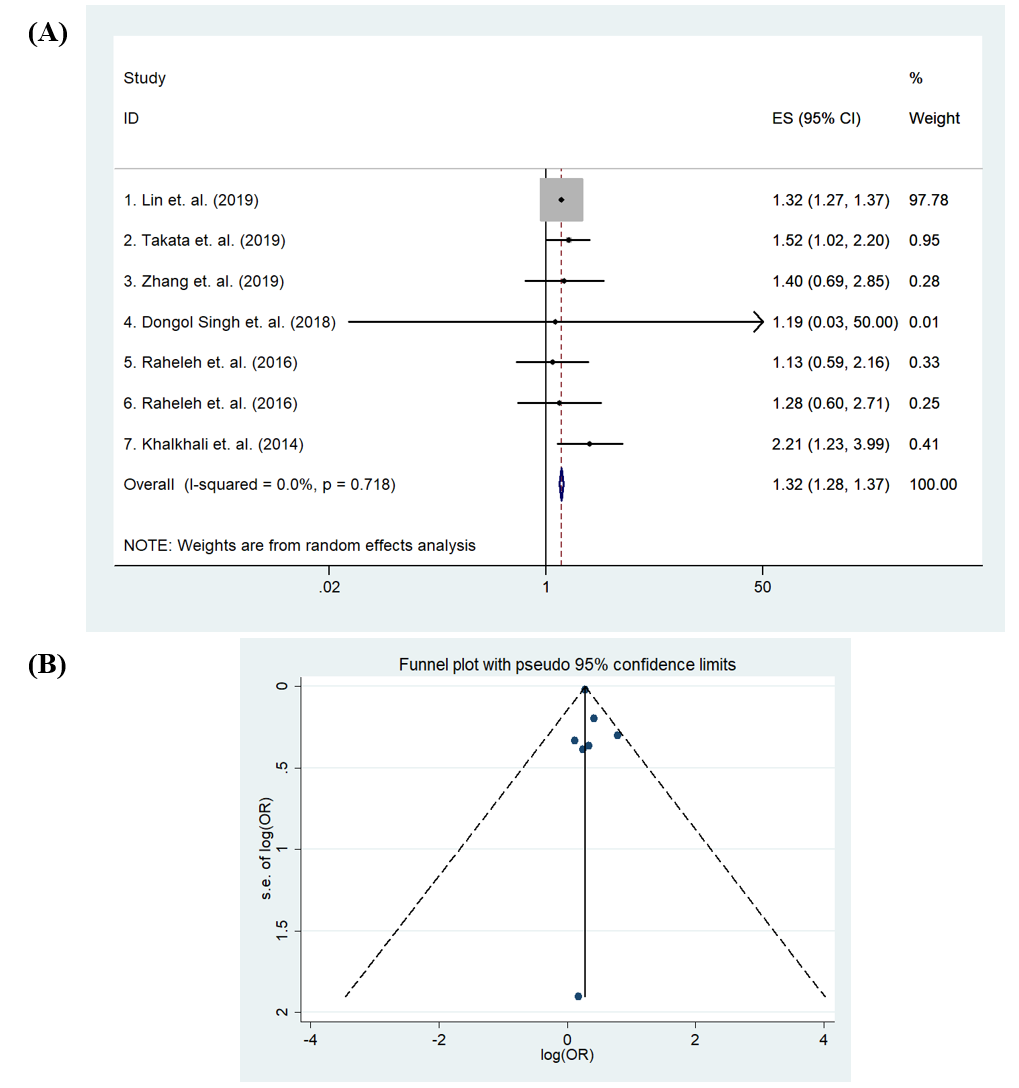


Figure S32. **(A)** Forest Plot and **(B)** Funnel Plot for Random-Effect Meta-Analysis for Preterm Birth (<= 37 weeks of gestational age) as Asthma Risk Factor Reported in 7 Independent Studies. Effect size (ES) is represented by the odds ratio (OR) and 95% confidence interval (CI) reported in each study. Results from the heterogeneity test, including the I^2^ value and the heterogeneity p-value (Het P) were also included in the figure. The funnel plot was plotted using the log(OR) and standard error (s.e.) of the log(OR) values from each study.


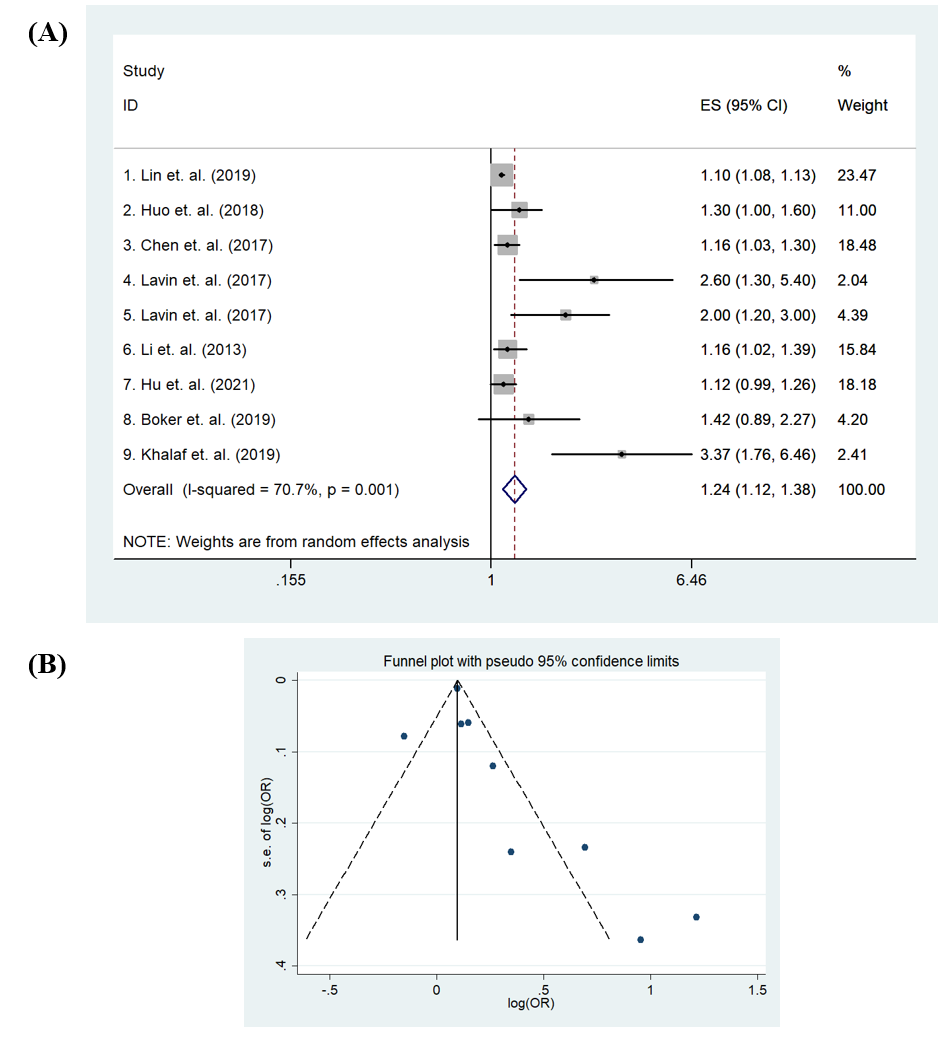


Figure S33. **(A)** Forest Plot and **(B)** Funnel Plot for Random-Effect Meta-Analysis for Method of Childbirth (Caesarean Section, Reference Category: Natural Birth) as Asthma Risk Factor Reported in 8 Independent Studies. Effect size (ES) is represented by the odds ratio (OR) and 95% confidence interval (CI) reported in each study. Results from the heterogeneity test, including the I^2^ value and the heterogeneity p-value (Het P) were also included in the figure. The funnel plot was plotted using the log(OR) and standard error (s.e.) of the log(OR) values from each study.
