# Supplementary material for: Risk factors of asthma in the Asian population: a systematic review and meta-analysis
Source: J Physiol Anthropol. 2021 Dec 9;40:22. doi: 10.1186/s40101-021-00273-x (PMC8662898; doi:10.1186/s40101-021-00273-x)
Supplement: Supplementary file 3 — Additional file 3: Table S2. Keywords used to perform literature search in three publication databases (Web of Science, Scopus, and Pubmed) to retrieve articles reporting asthma-associated risk factors in Asia. [file 40101_2021_273_MOESM3_ESM.docx]

**Supplementary Table S2.** Keywords Used to Perform Literature Search in Three Publication Databases (Web of Science, Scopus, and Pubmed) to Retrieve Articles Reporting Asthma-Associated Risk Factors in Asia.

| **No.** | **Database** | **No. of articles retrieved** | **Keywords** |
| --- | --- | --- | --- |
| **1** | **Web of Science** | **1209** | **TI=(Asthma*) AND TS=(epidemiology OR risk) AND TS=(Asia* OR China OR India OR Indonesia OR Pakistan OR Bangladesh OR Japan OR Philippines OR Vietnam OR Turkey OR Iran OR Thailand OR Myanmar OR "South Korea" OR Iraq OR Afghanistan OR "Saudi Arabia" OR Uzbekistan OR Malaysia OR Yemen OR Nepal OR "North Korea" OR "Sri Lanka" OR Kazakhstan OR Syria OR Cambodia OR Jordan OR Azerbaijan OR "United Arab Emirates" OR Tajikistan OR Israel OR Laos OR Lebanon OR Kyrgyzstan OR Turkmenistan OR Singapore OR "State of Palestine" OR Oman OR Kuwait OR Georgia OR Mongolia OR Armenia OR Qatar OR Bahrain OR Timor-Leste OR Cyprus OR Bhutan OR Maldives OR Brunei OR Taiwan OR "Hong Kong")** |
| **2** | **Scopus** | **1828** | **TITLE ( asthma* ) AND TITLE-ABS-KEY ( epidemiology OR risk ) AND TITLE-ABS-KEY ( asia* OR china OR india OR indonesia OR pakistan OR bangladesh OR japan OR philippines OR vietnam OR turkey OR iran OR thailand OR myanmar OR "South Korea" OR iraq OR afghanistan OR "Saudi Arabia" OR uzbekistan OR malaysia OR yemen OR nepal OR "North Korea" OR "Sri Lanka" OR kazakhstan OR syria OR cambodia OR jordan OR azerbaijan OR "United Arab Emirates" OR tajikistan OR israel OR laos OR lebanon OR kyrgyzstan OR turkmenistan OR singapore OR "State of Palestine" OR oman OR kuwait OR georgia OR mongolia OR armenia OR qatar OR bahrain OR timor-leste OR cyprus OR bhutan OR maldives OR brunei OR taiwan OR "Hong Kong" )** |
| **3** | **Pubmed** | **1027** | **((Asthma*[Title]) AND (epidemiology[Title/Abstract] OR risk[Title/Abstract])) AND (Asia*[Title/Abstract] OR China[Title/Abstract] OR India[Title/Abstract] OR Indonesia[Title/Abstract] OR Pakistan[Title/Abstract] OR Bangladesh[Title/Abstract] OR Japan[Title/Abstract] OR Philippines[Title/Abstract] OR Vietnam[Title/Abstract] OR Turkey[Title/Abstract] OR Iran[Title/Abstract] OR Thailand[Title/Abstract] OR Myanmar[Title/Abstract] OR "South Korea"[Title/Abstract] OR Iraq[Title/Abstract] OR Afghanistan[Title/Abstract] OR "Saudi Arabia"[Title/Abstract] OR Uzbekistan[Title/Abstract] OR Malaysia[Title/Abstract] OR Yemen[Title/Abstract] OR Nepal[Title/Abstract] OR "North Korea"[Title/Abstract] OR "Sri Lanka"[Title/Abstract] OR Kazakhstan[Title/Abstract] OR Syria[Title/Abstract] OR Cambodia[Title/Abstract] OR Jordan[Title/Abstract] OR Azerbaijan[Title/Abstract] OR "United Arab Emirates"[Title/Abstract] OR Tajikistan[Title/Abstract] OR Israel[Title/Abstract] OR Laos[Title/Abstract] OR Lebanon[Title/Abstract] OR Kyrgyzstan[Title/Abstract] OR Turkmenistan[Title/Abstract] OR Singapore[Title/Abstract] OR "State of Palestine"[Title/Abstract] OR Oman[Title/Abstract] OR Kuwait[Title/Abstract] OR Georgia[Title/Abstract] OR Mongolia[Title/Abstract] OR Armenia[Title/Abstract] OR Qatar[Title/Abstract] OR Bahrain[Title/Abstract] OR Timor-Leste[Title/Abstract] OR Cyprus[Title/Abstract] OR Bhutan[Title/Abstract] OR Maldives[Title/Abstract] OR Brunei[Title/Abstract] OR Taiwan[Title/Abstract] OR "Hong Kong"[Title/Abstract])** |
