# Supplementary material for: Risk factors of asthma in the Asian population: a systematic review and meta-analysis
Source: J Physiol Anthropol. 2021 Dec 9;40:22. doi: 10.1186/s40101-021-00273-x (PMC8662898; doi:10.1186/s40101-021-00273-x)
Supplement: Supplementary file 5 — Additional file 5: Table S4. Reported publications on asthma-associated risk factors from countries, dependencies, or other territories within Asia (1993-2021). [file 40101_2021_273_MOESM5_ESM.docx]

**Supplementary Table S4.** Reported Publications on Asthma-associated Risk Factors from Countries, Dependencies, or Other Territories within Asia (1993-2021).

| **No** | **Countries, Dependencies or other territories** | **Population Size (2019)*** | **Sub Region** | **No. of Publications** |
| --- | --- | --- | --- | --- |
| **-** | **Total** | **-** | **-** | **289** |
| 1 | Mainland China | 1,439,323,776 | Eastern Asia | 73 |
| 2 | Taiwan | 23,816,775 | Eastern Asia | 38 |
| 3 | Japan | 126,476,461 | Eastern Asia | 28 |
| 4 | India | 1,380,004,385 | Southern Asia | 21 |
| 5 | South Korea | 51,269,185 | Eastern Asia | 21 |
| 6 | Turkey | 84,339,067 | Western Asia | 14 |
| 7 | Iran | 83,992,949 | Western Asia | 14 |
| 8 | Saudi Arabia | 34,813,871 | Western Asia | 11 |
| 9 | Hong Kong | 7,496,981 | Eastern Asia | 8 |
| 10 | Lebanon | 6,825,445 | Western Asia | 7 |
| 11 | Israel | 8,655,535 | Western Asia | 7 |
| 12 | Malaysia | 32,365,999 | South-Eastern Asia | 5 |
| 13 | Pakistan | 220,892,340 | Southern Asia | 5 |
| 14 | Singapore | 5,850,342 | South-Eastern Asia | 5 |
| 15 | United Arab Emirates | 9,890,402 | Western Asia | 4 |
| 16 | Vietnam | 97,338,579 | South-Eastern Asia | 3 |
| 17 | Cyprus | 1,207,359 | Western Asia | 3 |
| 18 | Sri Lanka | 21,413,249 | Southern Asia | 3 |
| 19 | Kuwait | 4,270,571 | Western Asia | 3 |
| 20 | Nepal | 29,136,808 | Southern Asia | 3 |
| 21 | Thailand | 69,799,978 | South-Eastern Asia | 2 |
| 22 | State of Palestine | 5,101,414 | Western Asia | 2 |
| 23 | Bangladesh | 164,689,383 | Southern Asia | 2 |
| 24 | Iraq | 40,222,493 | Western Asia | 2 |
| 25 | Qatar | 2,881,053 | Western Asia | 2 |
| 26 | Indonesia | 273,523,615 | South-Eastern Asia | 1 |
| 27 | Kazakhstan | 18,776,707 | Central Asia | 1 |
| 28 | Georgia | 3,989,167 | Western Asia | 1 |

*Reference: United Nations, 2019 [1].

**Reference**

1. United Nations, Department of Economic and Social Affairs, Population Division: **World Population Prospects 2019: Volume I: Comprehensive Tables**. In*.* Edited by Department of Economic and Social Affairs PD, vol. 1. <https://population.un.org/wpp/Publications/Files/WPP2019_Volume-I_Comprehensive-Tables.pdf>: United Nations; 2019.
