# Supplementary material for: Risk factors of asthma in the Asian population: a systematic review and meta-analysis
Source: J Physiol Anthropol. 2021 Dec 9;40:22. doi: 10.1186/s40101-021-00273-x (PMC8662898; doi:10.1186/s40101-021-00273-x)
Supplement: Supplementary file 6 — Additional file 6: Table S5. Summary of frequently reported asthma-associated risk factors in the Asian population (1993-2021). [file 40101_2021_273_MOESM6_ESM.docx]

**Supplementary Table S5.** Summary of Frequently Reported Asthma-associated Risk Factors in the Asian Population (1993-2021).

| **No.** | **Risk Factors** |  | **No. of studies with significant associations** | **Ref^1^** | **No. of studies with mixed/ insignificant associations** | **Ref^2^** | **No. of studies included in Meta-analysis**  **(No. of sig. associations)** | **Ref^3^** |
| --- | --- | --- | --- | --- | --- | --- | --- | --- |
| **1** | **Family Medical History** | Overall | 85 | [1-85] | 6 | [86-91] | - | - |
|  |  | Asthma (any family members) | 36 | [3, 8-10, 15, 22-24, 26-28, 30, 31, 34-36, 38, 44, 48, 49, 51, 55, 58-61, 64-66, 71, 77, 79, 84, 85, 87, 90, 92] | 1 | [88] | 36 (36) | [3, 8-10, 15, 22-24, 26-28, 30, 31, 34-36, 38, 44, 48, 49, 51, 55, 58-61, 64-66, 71, 77, 79, 84, 85, 87, 90, 92] |
|  |  | Asthma (maternal) | 15 | [5, 12, 26, 39, 45, 46, 50-52, 55, 72, 81, 83, 89, 91] | 0 | - | 15 (15) | [5, 12, 26, 39, 45, 46, 50-52, 55, 72, 81, 83, 89, 91] |
|  |  | Asthma (paternal) | 10 | [12, 26, 39, 41, 45, 46, 50-52, 81] | 1 | [89] | 11 (10) | [12, 26, 39, 41, 45, 46, 50-52, 81, 89] |
|  |  | Asthma (parental) | 9 | [4, 13, 26, 40, 47, 53, 54, 73, 80] | 0 |  | 9 (9) | [4, 13, 26, 40, 47, 53, 54, 73, 80] |
|  |  | Asthma (siblings) | 4 | [26, 29, 55, 81] | 0 | - | 3 (3) | [26, 29, 81] |
|  |  | Atopy (any family members) | 10 | [14, 17-19, 25, 32, 57, 70, 78, 82] | 0 | - | 10 (10) | [14, 17-19, 25, 32, 57, 70, 78, 82] |
|  |  | Atopy (parental) | 7 | [2, 7, 37, 50, 63, 74, 76] | 0 | - | 7 (7) | [2, 7, 37, 50, 63, 74, 76] |
|  |  | Allergic Diseases (any family members) | 14 | [3, 11, 21, 33, 39, 43, 45, 56, 62, 67-69, 75, 84] | 1 | [87] | 14 (13) | [3, 11, 21, 33, 39, 43, 45, 56, 62, 67-69, 75, 84, 87] |
|  |  | Allergic Diseases (paternal) | 3 | [6, 39, 45] | 1 | [86] | 4 (3) | [6, 39, 45, 86] |
|  |  | Allergic Rhinitis / Pollinosis (any family members) | 4 | [9, 35, 60, 90] | 0 | - | 4 (4) | [9, 35, 60, 90] |
|  |  | Allergic Rhinitis / Pollinosis (maternal) | 2 | [81, 91] | 1 | [89] | 3 (2) | [81, 89, 91] |
|  |  | Atopic Dermatitis / Eczema (any family members) | 3 | [9, 59, 60] | 1 | [90] | 4 (3) | [9, 59, 60, 90] |
| **2** | **Housing (condition, environment, size, type, etc.)** | Overall | 53 | [4, 11, 14, 15, 18, 19, 22, 28, 33, 37, 42, 47, 48, 50, 52, 66, 80, 81, 85, 93-126] | 23 | [2, 26, 63, 67, 74, 76, 79, 83, 88, 127-140] | - | - |
|  |  | Household Dampness | 16 | [2, 4, 42, 48, 67, 80, 97, 99, 100, 103, 109, 119, 124, 127, 128, 139] | 2 | [88, 133] | - | - |
|  |  | Presence of Mold, Mold spots | 10 | [37, 50, 52, 66, 76, 103, 104, 112, 127, 128] | 1 | [133] | 10 (9) | [37, 50, 52, 66, 76, 103, 104, 112, 128, 133] |
|  |  | Presence of Mold Odor | 7 | [99, 100, 103, 104, 112, 127, 128] | 3 | [2, 133, 136] | 10 (7) | [2, 99, 100, 103, 104, 112, 127, 128, 133, 136] |
|  |  | Cockroach Presence/Exposure | 8 | [19, 37, 52, 76, 112, 116, 118, 131] | 1 | [67] | 6 (5) | [19, 37, 67, 112, 118, 131] |
|  |  | Carpet usage | 3 | [67, 74, 102] | 2 | [88, 112] | 5 (3) | [67, 74, 88, 102, 112] |
|  |  | Housing Size | 2 | [28, 130] | 3 | [26, 63, 127] | - | - |
|  |  | Housing Type | 4 | [26, 115, 120, 130] | 3 | [74, 137, 138] | - | - |
|  |  | Incense Burning | 4 | [52, 123, 125, 131] | 2 | [88, 129] | 6 (4) | [52, 88, 123, 125, 129, 131] |
|  |  | Traffic Pollution | 10 | [63, 66, 67, 96, 98, 101, 102, 114, 126, 131] | 4 | [74, 132, 135, 140] | - | - |
|  |  | Type of Air-Conditioning System or Heater | 4 | [4, 106, 121, 140] | 1 | [137] | - | - |
|  |  | Presence of Water Damage / Water Leakage | 5 | [37, 107, 110, 127, 133] | 3 | [76, 128, 136] | 5 (4) | [37, 76, 107, 127, 133] |
|  |  | Windowpane condensation in winter | 2 | [104, 127] | 3 | [128, 133, 136] | - | - |
| **3** | **Gender** | Overall | 60 | [3, 12, 13, 15, 16, 18, 20, 27, 28, 32, 36, 42, 44, 47-49, 52, 54, 57, 62, 63, 67, 68, 71, 74-76, 79, 83, 89, 91, 96, 98, 111, 123, 125-127, 138, 141-161] | 15 | [1, 2, 65, 77, 78, 82, 86, 88, 162-168] | 73 (59) | [1-3, 12, 13, 15, 16, 18, 20, 27, 28, 32, 36, 42, 44, 47-49, 52, 54, 57, 63, 65, 67, 68, 71, 74-79, 82, 83, 86, 89, 91, 96, 98, 111, 123, 125-127, 138, 141-168] |
|  |  | Male | 51 | [3, 12, 15, 16, 18, 20, 27, 28, 32, 42, 44, 47-49, 52, 54, 57, 62, 63, 67, 68, 74-76, 79, 83, 88, 89, 91, 96, 98, 111, 123, 125-127, 138, 141-153, 160, 161, 164] | 7 | [78, 82, 86, 163, 165, 167, 168] | - | - |
|  |  | Female | 9 | [13, 36, 71, 154-159] | 6 | [1, 2, 65, 77, 162, 166] | - | - |
| **4** | **Age** | - | 33 | [15, 19, 22, 23, 27, 32, 34, 35, 37, 42, 52, 57, 62, 63, 65, 79, 120, 123, 125, 134, 138, 147, 154, 155, 157-159, 164, 166, 169-172] | 17 | [1, 2, 67, 68, 73-78, 82, 88, 137, 161, 162, 168, 173] | - | - |
| **5** | **Cigarette Smoke Exposure** | - | 44 | [6, 12, 13, 21, 23, 27, 28, 32, 33, 37, 40-42, 44, 50-52, 57, 89, 93, 95, 100, 101, 114, 115, 121, 123, 125, 126, 138, 155, 160-162, 165, 171, 174-181] | 19 | [63, 70, 73-76, 79, 82, 83, 86-88, 112, 127, 129, 131, 136, 168, 182] | 21 (17) | [6, 12, 23, 32, 37, 40, 63, 76, 87, 89, 115, 123, 127, 136, 138, 155, 162, 174-177] |
| **6** | **Cigarette Smoking** | - | 31 | [1, 6, 21, 23, 26, 31, 38, 64, 65, 90, 115, 120, 123, 125, 142, 152, 157, 158, 161, 165, 169-171, 175, 181-187] | 5 | [82, 88, 127, 138, 162] | 28 (25) | [1, 6, 21, 23, 26, 31, 38, 64, 82, 90, 123, 125, 127, 138, 152, 157, 158, 161, 162, 165, 169-171, 175, 183-186] |
| **7** | **Body Mass Index (BMI)-related** | Overall | 29 | [8, 74, 90, 106, 115, 120, 125, 142-144, 155, 157, 158, 161, 162, 166, 170, 176, 183, 188-197] | 8 | [1, 88, 198-203] | - | - |
|  |  | BMI (kg/m2) | 8 | [74, 106, 125, 158, 162, 166, 176, 204] | 2 | [199, 201] | 8 (7) | [74, 106, 125, 158, 162, 166, 176, 201] |
|  |  | Obese (BMI > 30kg/m2) | 7 | [115, 120, 142, 157, 170, 192, 195] | 1 | [198] | 8 (7) | [115, 120, 142, 157, 170, 192, 195, 198] |
|  |  | Obese (BMI >= 95 percentile) | 6 | [8, 189, 191, 193, 196, 203] | 1 | [202] | 7 (6) | [8, 189, 191, 193, 196, 202, 203] |
|  |  | Underweight (BMI < 18 or 18.5kg/m2) | 2 | [120, 142] | 2 | [1, 198] | 4 (2) | [1, 120, 142, 198] |
|  |  | Underweight (BMI < 5th percentile) | 1 | [193] | 2 | [202, 203] | 3 (1) | [193, 202, 203] |
| **8** | **Pet Exposure** | Overall | 22 | [6, 18, 23, 32, 39, 40, 42, 45, 50, 51, 67, 69, 74, 78, 96, 98, 112, 118, 122, 126, 176, 205] | 11 | [70, 79, 82, 87, 88, 90, 131, 137, 160, 206, 207] | - |  |
|  |  | Dogs | 7 | [6, 39, 45, 78, 90, 96, 112] | 3 | [131, 206, 207] | - |  |
|  |  | Cats | 7 | [6, 39, 45, 51, 126, 131, 206] | 2 | [90, 207] | - |  |
| **9** | **Educational Level** | Overall | 20 | [1, 3, 13, 21, 27, 52, 56, 62, 81, 115, 120, 123, 125, 126, 144, 157, 158, 170, 171, 187] | 11 | [26, 73, 74, 76, 86, 88, 137, 138, 160, 164, 173] | - |  |
|  |  | Participant's | 7 | [1, 115, 120, 157, 158, 170, 187] | 6 | [26, 88, 137, 138, 164, 173] | - |  |
|  |  | Parental | 13 | [3, 13, 21, 27, 52, 56, 62, 81, 123, 125, 126, 144, 171] | 5 | [73, 74, 76, 86, 160] | - |  |
| **10** | **Urbanization (Living in urban, suburban, rural, etc.)** | - | 20 | [21, 28, 32, 53, 56, 63, 66, 91, 109, 147-150, 158, 162, 164, 170, 171, 208, 209] | 8 | [1, 73, 86, 89, 127, 131, 138, 160] | - | - |
| **11** | **Air Pollution** | Overall | 14 | [37, 52, 74, 102, 144, 145, 171, 175, 186, 208, 210-213] | 10 | [1, 63, 76, 129, 131, 134, 214-217] | - | - |
|  |  | NO_2_ | 8 | [63, 74, 129, 131, 208, 210, 213, 215] | 1 | [214] | 6 (5) | [63, 74, 129, 131, 208, 214] |
|  |  | PM10 | 6 | [74, 76, 144, 145, 171, 208] | 5 | [63, 129, 131, 215, 216] | 8 (5) | [63, 74, 129, 131, 144, 145, 171, 208] |
|  |  | O_3_ | 3 | [74, 76, 171] | 1 | [214] | 3 (2) | [74, 171, 214] |
|  |  | CO | 2 | [76, 171] | 1 | [214] | - | - |
|  |  | Nitrogen Oxides | 1 | [211] | 2 | [76, 217] | - | - |
|  |  | PM2.5 | 2 | [212, 214] | 1 | [1] | - | - |
|  |  | SO_2_ | 3 | [74, 213, 215] | 2 | [76, 214] | - | - |
| **12** | **Breastfeeding** | Overall | 18 | [3, 5, 29, 37, 51, 54, 63, 68, 70, 71, 74, 81, 87, 90, 106, 218-220] | 8 | [2, 62, 75, 160, 168, 221-223] | - | - |
|  |  | Exclusive breastfeeding | 4 | [54, 87, 219, 220] | 4 | [62, 160, 221, 223] | 4 (3) | [54, 87, 160, 219] |
| **13** | **Dietary Habits** | Overall | 19 | [7, 11, 17, 29, 56, 78, 96, 98, 106, 119, 144, 149, 154, 157, 209, 224-227] | 4 | [3, 87, 161, 228] | - | - |
|  |  | Dairy Products | 7 | [3, 7, 56, 78, 96, 154, 157] | 0 | - | - | - |
|  |  | Fruits | 6 | [17, 96, 119, 157, 161, 224] | 0 | - | - | - |
|  |  | Meat (chicken, red meat, etc.) | 6 | [3, 78, 106, 154, 157, 224] | 0 | - | - | - |
|  |  | Seafood (includes fish) | 4 | [96, 209, 226, 227] | 0 | - | - | - |
|  |  | Vegetables | 3 | [56, 96, 157] | 0 | - | - | - |
|  |  | Junk Food | 2 | [98, 224] | 2 | [3, 161] | - | - |
| **14** | **Cooking Fumes Exposure / Type of Cooking Fuel** | - | 14 | [23, 44, 47, 67, 96, 119-121, 130, 138, 169, 170, 186, 229] | 7 | [1, 26, 129, 131, 134, 137, 140] | - | - |
| **15** | **Socioeconomic Status** | - | 13 | [4, 28, 43, 46, 71, 115, 138, 144, 159, 160, 168, 169, 203] | 8 | [73, 75, 88, 89, 137, 164, 165, 173] | - | - |
| **16** | **Occupational Exposure** |  | 10 | [10, 31, 115, 120, 183, 185, 187, 229-231] | 8 | [1, 88, 137, 162, 164, 173, 186, 232] | - | - |
| **17** | **Medication Usage** | Overall | 16 | [5, 11, 17, 24, 29, 47, 62, 67, 71, 75, 84, 106, 126, 129, 233, 234] | 2 | [70, 220] | - | - |
|  |  | Antibiotics | 13 | [5, 11, 24, 29, 62, 67, 71, 75, 84, 106, 129, 233, 234] | 1 | [70] | - | - |
|  |  | Paracetamol | 5 | [17, 24, 47, 106, 126] | 0 | - | - | - |
| **18** | **Birth Weight** | - | 8 | [40, 67, 69, 71, 91, 136, 160, 235] | 9 | [2, 62, 63, 86, 87, 199, 220, 236, 237] | 6 (4)* | [63, 86, 91, 160, 220, 236] |
| **19** | **Region of Residence** | - | 14 | [59, 95, 114, 120, 123, 145, 147, 153, 157, 169, 176, 238-240] | 2 | [82, 138] | - | - |
| **20** | **Ethnicity/Nationality** | - | 10 |  | 2 |  | - | - |
| **21** | **Gestational Age** | Overall | 6 | [70, 71, 84, 91, 235, 236] | 5 | [2, 62, 87, 220, 237] | - | - |
|  |  | Preterm Birth (<= 37 weeks) | 4 | [71, 84, 91, 236] | 4 | [2, 87, 220, 237] | 6 (3) | [2, 84, 87, 91, 236, 237] |
| **22** | **Method of Childbirth** | - | 9 | [62, 68, 71, 91, 144, 219, 241-243] | 1 | [70] | 8 (7) | [62, 68, 70, 71, 91, 144, 241, 242] |
| **23** | **No. of Siblings** | - | 8 | [9, 17, 21, 24, 52, 53, 91, 160] | 1 | [86] | - | - |
| **24** | **Weather** | - | 6 | [63, 129, 131, 144, 171, 244] | 2 | [127, 134] | - | - |
| **25** | **Urinary Metabolites** | - | 4 | [245-248] | 3 | [249-251] | - | - |
| **26** | **Exercise / Physical Activity** | - | 7 | [96, 123, 125, 142, 161, 171, 202] | 0 | - | - | - |
| **27** | **Day-care Attendance** | - | 3 | [42, 67, 219] | 2 | [51, 75] | - | - |
| **28** | **Indoor Dust Content** | - | 3 | [7, 20, 252] | 1 | [253] | - | - |
| **29** | **Maternal Age at Delivery** | - | 5 | [5, 62, 70, 91, 146] | 0 | - | - | - |
| **30** | **Alcohol Consumption** | - | 4 | [123, 157, 170, 171] | 0 | - | - | - |
| **31** | **Parental Consanguinity** | - | 3 | [30, 41, 254] | 0 | - | - | - |

Only risk factors with >= three independent studies that showed overall significant asthma-association will be summarized. All reference numbers mentioned in this table referred to bibliographies listed in the references section below. *Meta-analysis for birth weight focused on birth weight less than 2500g. **Ref^1^**: studies reported a significant association between risk factor and asthma; **Ref^2^**: studies reported a mixed or insignificant association between risk factor and asthma. **Ref^3^**: all studies included in the meta-analysis.

**Reference**

1. Huang, K., et al., *Prevalence, risk factors, and management of asthma in China: a national cross-sectional study.* Lancet, 2019. **394**(10196): p. 407-418.

2. Zhang, S., et al., *Association between parental perceptions of odors and childhood asthma in subtropical South China with a hot humid climate.* BUILDING AND ENVIRONMENT, 2019. **159**.

3. Malaeb, D., et al., *Diet and asthma in Lebanese schoolchildren: A cross-sectional study.* Pediatr Pulmonol, 2019. **54**(6): p. 688-697.

4. Beridze, V., et al., *Childhood asthma in Batumi, Georgia: Prevalence and environmental correlates.* J Asthma, 2018. **55**(1): p. 43-49.

5. Kashanian, M., et al., *Evaluation of the associations between childhood asthma and prenatal and perinatal factors.* Int J Gynaecol Obstet, 2017. **137**(3): p. 290-294.

6. Ziyab, A.H., *Prevalence and Risk Factors of Asthma, Rhinitis, and Eczema and Their Multimorbidity among Young Adults in Kuwait: A Cross-Sectional Study.* Biomed Res Int, 2017. **2017**: p. 2184193.

7. Feng, M., et al., *Associations of Early Life Exposures and Environmental Factors With Asthma Among Children in Rural and Urban Areas of Guangdong, China.* Chest, 2016. **149**(4): p. 1030-41.

8. Qureshi, U.A., et al., *Epidemiology of bronchial asthma in school children (10-16 years) in Srinagar.* Lung India, 2016. **33**(2): p. 167-73.

9. Danansuriya, M.N., L.C. Rajapaksa, and A. Weerasinghe, *Genetic, familial and environmental correlates of asthma among early adolescents in Sri Lanka: a case control study.* World Allergy Organ J, 2015. **8**(1): p. 19.

10. Wortong, D., N. Chaiear, and W. Boonsawat, *Risk of asthma in relation to occupation: A hospital-based case-control study.* Asian Pac J Allergy Immunol, 2015. **33**(2): p. 152-60.

11. Tang, S.P., et al., *Trends in prevalence and risk factors of childhood asthma in Fuzhou, a city in Southeastern China.* J Asthma, 2015. **52**(1): p. 10-5.

12. Tavacol, H., et al., *A cross-sectional study of prevalence and risk factors for childhood asthma in Ahvaz city, Iran.* Postepy Dermatol Alergol, 2015. **32**(4): p. 268-73.

13. Abidin, E., et al., *The relationship between air pollution and asthma in Malaysian schoolchildren.* AIR QUALITY ATMOSPHERE AND HEALTH, 2014. **7**(4): p. 12.

14. Becerir, T., et al., *Prevalence of asthma, local risk factors and agreement between written and video questionnaires among Turkish adolescents.* Allergol Immunopathol (Madr), 2014. **42**(6): p. 594-602.

15. Khan, A.A., et al., *Burden of asthma among children in a developing megacity: childhood asthma study, Pakistan.* J Asthma, 2014. **51**(9): p. 891-9.

16. Chae, Y., et al., *Indoor environmental factors associated with wheezing illness and asthma in South Korean children: phase III of the International Study of Asthma and Allergies in Childhood.* J Asthma, 2014. **51**(9): p. 943-9.

17. Akcay, A., et al., *Risk factors affecting asthma prevalence in adolescents living in Istanbul, Turkey.* Allergol Immunopathol (Madr), 2014. **42**(5): p. 449-58.

18. Duksal, F., et al., *The prevalence of asthma diagnosis and symptoms is still increasing in early adolescents in Turkey.* Allergol Int, 2014. **63**(2): p. 189-97.

19. Ugurlu, E., S.B. Oncel, and F. Evyapan, *Symptom prevalence and risk factors for asthma at the rural regions of Denizli, Turkey.* J Thorac Dis, 2014. **6**(5): p. 452-8.

20. Norback, D., et al., *Endotoxin, ergosterol, fungal DNA and allergens in dust from schools in Johor Bahru, Malaysia- associations with asthma and respiratory infections in pupils.* PLoS One, 2014. **9**(2): p. e88303.

21. Lamnisos, D., et al., *Prevalence of asthma and allergies in children from the Greek-Cypriot and Turkish-Cypriot communities in Cyprus: a bi-communal cross-sectional study.* BMC Public Health, 2013. **13**: p. 585.

22. Wang, D., et al., *Cross-sectional epidemiological survey of asthma in Jinan, China.* Respirology, 2013. **18**(2): p. 313-22.

23. Jie, Y., et al., *Asthma and Asthma-Related Symptoms among Adults of an Acid Rain-Plagued City in Southwest China: Prevalence and Risk Factors.* POLISH JOURNAL OF ENVIRONMENTAL STUDIES, 2013. **22**(3): p. 10.

24. Oshnouei, S., et al., *Effects of Acetaminophen consumption in asthmatic children.* Iran Red Crescent Med J, 2012. **14**(10): p. 641-6.

25. Nathan, A.M., et al., *Caesarean section and asthma in Malaysian children: a case-control study.* Asian Pac J Allergy Immunol, 2012. **30**(3): p. 204-8.

26. Ding, Y.P., et al., *An epidemiology study of bronchial asthma in the Li ethnic group in China.* Asian Pac J Trop Med, 2012. **5**(2): p. 157-61.

27. Lee, S.L., et al., *Foetal exposure to maternal passive smoking is associated with childhood asthma, allergic rhinitis, and eczema.* ScientificWorldJournal, 2012. **2012**: p. 542983.

28. Hwang, G.S., et al., *Residential environmental risk factors for childhood asthma prevalence in metropolitan and semirural cities in Korea.* Asia Pac J Public Health, 2012. **24**(1): p. 58-67.

29. Yeh, K.W., et al., *Prevalence and risk factors for early presentation of asthma among preschool children in Taiwan.* Asian Pac J Allergy Immunol, 2011. **29**(2): p. 120-6.

30. Mahdi, B., et al., *Inheritance patterns, consanguinity & risk for asthma.* Indian J Med Res, 2010. **132**: p. 48-55.

31. Cakir, E., et al., *The prevalence and risk factors of asthma and allergic diseases among working adolescents.* Asian Pac J Allergy Immunol, 2010. **28**(2-3): p. 122-9.

32. Selcuk, Z.T., et al., *Prevalence of asthma and allergic diseases in primary school children in Edirne, Turkey, two surveys 10 years apart.* Pediatr Allergy Immunol, 2010. **21**(4 Pt 2): p. e711-7.

33. Uthaisangsook, S., *Risk factors for development of asthma in Thai adults in Phitsanulok: a university-based study.* Asian Pac J Allergy Immunol, 2010. **28**(1): p. 23-8.

34. Jain, A., H. Vinod Bhat, and D. Acharya, *Prevalence of bronchial asthma in rural Indian children: a cross sectional study from South India.* Indian J Pediatr, 2010. **77**(1): p. 31-5.

35. Alsowaidi, S., et al., *Allergic rhinitis and asthma: a large cross-sectional study in the United Arab Emirates.* Int Arch Allergy Immunol, 2010. **153**(3): p. 274-9.

36. Alsowaidi, S., A. Abdulle, and R. Bernsen, *Prevalence and risk factors of asthma among adolescents and their parents in Al-Ain (United Arab Emirates).* Respiration, 2010. **79**(2): p. 105-11.

37. Han, Y.Y., Y.L. Lee, and Y.L. Guo, *Indoor environmental risk factors and seasonal variation of childhood asthma.* Pediatr Allergy Immunol, 2009. **20**(8): p. 748-56.

38. Yoo, S., et al., *Effect of active smoking on asthma symptoms, pulmonary function, and BHR in adolescents.* Pediatr Pulmonol, 2009. **44**(10): p. 954-61.

39. Dong, G.H., et al., *Pets keeping in home, parental atopy, asthma, and asthma-related symptoms in 12,910 elementary school children from northeast China.* Indoor Air, 2009. **19**(2): p. 166-73.

40. Fernando, D., et al., *Toxocara seropositivity in Sri Lankan children with asthma.* Pediatr Int, 2009. **51**(2): p. 241-5.

41. Joseph, M., et al., *Paternal asthma is a predictor for childhood asthma in the consanguineous families from the United Arab Emirates.* J Asthma, 2009. **46**(2): p. 175-8.

42. Waked, M. and P. Salameh, *Risk factors for asthma and allergic diseases in school children across Lebanon.* J Asthma Allergy, 2008. **2**: p. 1-7.

43. Talay, F., et al., *Prevalence and risk factors of asthma and allergic diseases among schoolchildren in Bolu, Turkey.* Acta Paediatr, 2008. **97**(4): p. 459-62.

44. Pakhale, S., et al., *Prevalence of asthma symptoms in 7th- and 8th-grade school children in a rural region in India.* J Asthma, 2008. **45**(2): p. 117-22.

45. Dong, G.H., et al., *Asthma and asthma-related symptoms in 16 789 Chinese children in relation to pet keeping and parental atopy.* J Investig Allergol Clin Immunol, 2008. **18**(3): p. 207-13.

46. Zaman, K., et al., *Asthma in rural Bangladeshi children.* Indian J Pediatr, 2007. **74**(6): p. 539-43.

47. Wong, G.W., et al., *Symptoms of asthma and atopic disorders in preschool children: prevalence and risk factors.* Clin Exp Allergy, 2007. **37**(2): p. 174-9.

48. Ones, U., et al., *Rising trend of asthma prevalence among Turkish schoolchildren (ISAAC phases I and III).* Allergy, 2006. **61**(12): p. 1448-53.

49. Gazala, E., et al., *The association between birth season and future development of childhood asthma.* Pediatr Pulmonol, 2006. **41**(12): p. 1125-8.

50. Lee, Y.L., et al., *Home exposures, parental atopy, and occurrence of asthma symptoms in adulthood in southern Taiwan.* Chest, 2006. **129**(2): p. 300-8.

51. Al-Mousawi, M.S., et al., *Asthma and sensitization in a community with low indoor allergen levels and low pet-keeping frequency.* J Allergy Clin Immunol, 2004. **114**(6): p. 1389-94.

52. Lee, Y.L., et al., *Indoor and outdoor environmental exposures, parental atopy, and physician-diagnosed asthma in Taiwanese schoolchildren.* Pediatrics, 2003. **112**(5): p. e389.

53. Shohat, T., et al., *Differences in the prevalence of asthma and current wheeze between Jews and Arabs: results from a national survey of schoolchildren in Israel.* Ann Allergy Asthma Immunol, 2002. **89**(4): p. 386-92.

54. Takemura, Y., et al., *Relation between breastfeeding and the prevalence of asthma : the Tokorozawa Childhood Asthma and Pollinosis Study.* Am J Epidemiol, 2001. **154**(2): p. 115-9.

55. Wang, T.N., et al., *Familial risk of asthma among adolescents and their relatives in Taiwan.* J Asthma, 2001. **38**(6): p. 485-94.

56. Hijazi, N., B. Abalkhail, and A. Seaton, *Diet and childhood asthma in a society in transition: a study in urban and rural Saudi Arabia.* Thorax, 2000. **55**(9): p. 775-9.

57. Chhabra, S.K., et al., *Risk factors for development of bronchial asthma in children in Delhi.* Ann Allergy Asthma Immunol, 1999. **83**(5): p. 385-90.

58. Ones, U., et al., *Prevalence of childhood asthma in Istanbul, Turkey.* Allergy, 1997. **52**(5): p. 570-5.

59. Leung, R., et al., *Sensitization to inhaled allergens as a risk factor for asthma and allergic diseases in Chinese population.* J Allergy Clin Immunol, 1997. **99**(5): p. 594-9.

60. Moussa, M.A., et al., *Factors associated with asthma in school children.* Eur J Epidemiol, 1996. **12**(6): p. 583-8.

61. Leung, R. and P. Ho, *Asthma, allergy, and atopy in three south-east Asian populations.* Thorax, 1994. **49**(12): p. 1205-10.

62. Huo, X., et al., *The effect of breastfeeding on the risk of asthma in high-risk children: a case-control study in Shanghai, China.* BMC Pregnancy Childbirth, 2018. **18**(1): p. 341.

63. Norback, D., et al., *Asthma and rhinitis among Chinese children - Indoor and outdoor air pollution and indicators of socioeconomic status (SES).* Environ Int, 2018. **115**: p. 1-8.

64. Lam, H.T., et al., *Increase in asthma and a high prevalence of bronchitis: results from a population study among adults in urban and rural Vietnam.* Respir Med, 2011. **105**(2): p. 177-85.

65. Oshikata, C., et al., *Increase in asthma prevalence in adults in temporary housing after the Great East Japan earthquake.* International Journal of Disaster Risk Reduction, 2020. **50**.

66. Idani, E., et al., *Risk factors associated with asthma among adults in Khuzestan, southwest Iran.* Clinical Epidemiology and Global Health, 2020. **8**(2): p. 6.

67. Huang, S., et al., *Home environmental and lifestyle factors associated with asthma, rhinitis and wheeze in children in Beijing, China.* Environ Pollut, 2020. **256**: p. 113426.

68. Hu, Y., et al., *Breastfeeding duration modified the effects of neonatal and familial risk factors on childhood asthma and allergy: a population-based study.* Respir Res, 2021. **22**(1): p. 41.

69. Arif, A.A. and S.D. Veri, *The association of prenatal risk factors with childhood asthma.* J Asthma, 2019. **56**(10): p. 1056-1061.

70. Boker, F., et al., *Cesarean Section and Development of Childhood Bronchial Asthma: Is There A Risk?* Open Access Maced J Med Sci, 2019. **7**(3): p. 347-351.

71. Al Yassen, A.Q., J.N. Al-Asadi, and S.K. Khalaf, *The role of Caesarean section in childhood asthma.* Malays Fam Physician, 2019. **14**(3): p. 10-17.

72. Spiegel, E., et al., *Maternal Asthma Is an Independent Risk Factor for Long-Term Respiratory Morbidity of the Offspring.* Am J Perinatol, 2018. **35**(11): p. 1065-1070.

73. Jang, Y. and A. Shin, *Sex-Based Differences in Asthma among Preschool and School-Aged Children in Korea.* PLOS ONE, 2015. **10**(e0140057).

74. Liu, F., et al., *Asthma and asthma related symptoms in 23,326 Chinese children in relation to indoor and outdoor environmental factors: the Seven Northeastern Cities (SNEC) Study.* Sci Total Environ, 2014. **497-498**: p. 10-17.

75. Tan, T.N., et al., *Prevalence of asthma and comorbid allergy symptoms in Singaporean preschoolers.* Asian Pac J Allergy Immunol, 2006. **24**(4): p. 175-82.

76. Hwang, B.F., et al., *Traffic related air pollution as a determinant of asthma among Taiwanese school children.* Thorax, 2005. **60**(6): p. 467-73.

77. Yang, M., et al., *Plasma antibodies against heat shock protein 70 correlate with the incidence and severity of asthma in a Chinese population.* Respir Res, 2005. **6**: p. 18.

78. Demir, A.U., et al., *Asthma and allergic diseases in schoolchildren: third cross-sectional survey in the same primary school in Ankara, Turkey.* Pediatr Allergy Immunol, 2004. **15**(6): p. 531-8.

79. Kawada, T., *Risk factors and prevalence of asthma or atopic dermatitis in young children by a questionnaire survey.* J Nippon Med Sch, 2004. **71**(3): p. 167-71.

80. Huang, S.L., P.F. Tsai, and Y.F. Yeh, *Negative association of Enterobius infestation with asthma and rhinitis in primary school children in Taipei.* Clin Exp Allergy, 2002. **32**(7): p. 1029-32.

81. Karunasekera, K.A., J.A. Jayasinghe, and L.W. Alwis, *Risk factors of childhood asthma: a Sri Lankan study.* J Trop Pediatr, 2001. **47**(3): p. 142-5.

82. Kalyoncu, A.F., et al., *Asthma and allergy in Turkish university students: Two cross-sectional surveys 5 years apart.* Allergol Immunopathol (Madr), 2001. **29**(6): p. 264-71.

83. Hallit, S., et al., *Hygiene hypothesis: association between hygiene and asthma among preschool children in Lebanon.* Allergol Immunopathol (Madr), 2021. **49**(1): p. 135-145.

84. Khalkhali, H.R., et al., *Effects of antibiotic consumption on children 2-8 years of age developing asthma.* Epidemiol Health, 2014. **36**: p. e2014006.

85. Al-Mazam, A. and A.G. Mohamed, *Risk factors of bronchial asthma in bahrah, saudi arabia.* J Family Community Med, 2001. **8**(1): p. 33-9.

86. Toizumi, M., et al., *Asthma, Rhinoconjunctivitis, Eczema, and the Association with Perinatal Anthropometric Factors in Vietnamese Children.* Sci Rep, 2019. **9**(1): p. 2655.

87. Dongol Singh, S. and A. Shrestha, *Risk Factors Associated with Childhood Asthma - A Case Control Study.* Kathmandu Univ Med J (KUMJ), 2018. **16**(64): p. 290-295.

88. Razzaq, S., et al., *Epidemiology of asthma and associated factors in an urban Pakistani population: adult asthma study-Karachi.* BMC Pulm Med, 2018. **18**(1): p. 184.

89. Huang, C.C., et al., *Risk factors for asthma occurrence in children with early-onset atopic dermatitis: An 8-year follow-up study.* Pediatr Allergy Immunol, 2018. **29**(2): p. 159-165.

90. Lin, J., et al., *Prevalence and risk factors of asthma in mainland China: The CARE study.* Respir Med, 2018. **137**: p. 48-54.

91. Lin, C.H., et al., *Shared prenatal impacts among childhood asthma, allergic rhinitis and atopic dermatitis: a population-based study.* ALLERGY ASTHMA AND CLINICAL IMMUNOLOGY, 2019. **15**(1).

92. Mathew, A., et al., *Prevalence and Risk Factors of Asthma in School Going Children in South India.* NEPAL JOURNAL OF EPIDEMIOLOGY, 2012. **2**(1): p. 8.

93. Sun, Y., et al., *Modern life makes children allergic. A cross-sectional study: associations of home environment and lifestyles with asthma and allergy among children in Tianjin region, China.* Int Arch Occup Environ Health, 2019. **92**(4): p. 587-598.

94. Zhang, J., et al., *Associations of household renovation materials and periods with childhood asthma, in China: A retrospective cohort study.* Environ Int, 2018. **113**: p. 240-248.

95. Hallit, S., et al., *Association between Caregiver Exposure to Toxics during Pregnancy and Childhood-onset Asthma: A Case-control Study.* Iran J Allergy Asthma Immunol, 2017. **16**(6): p. 488-500.

96. Alqahtani, J.M., et al., *Environmental Determinants of Bronchial Asthma among Saudi School Children in Southwestern Saudi Arabia.* Int J Environ Res Public Health, 2016. **14**(1).

97. Takaoka, M., K. Suzuki, and D. Norback, *Current asthma, respiratory symptoms and airway infections among students in relation to the school and home environment in Japan.* J Asthma, 2017. **54**(6): p. 652-661.

98. Alqahtani, J.M., *Asthma and other allergic diseases among Saudi schoolchildren in Najran: the need for a comprehensive intervention program.* Ann Saudi Med, 2016. **36**(6): p. 379-385.

99. Bu, Z., et al., *Associations between perceptions of odors and dryness and children's asthma and allergies: A cross-sectional study of home environment in Baotou.* BUILDING AND ENVIRONMENT, 2016. **106**: p. 8.

100. Lin, Z., et al., *The first 2-year home environment in relation to the new onset and remission of asthmatic and allergic symptoms in 4246 preschool children.* Sci Total Environ, 2016. **553**: p. 204-210.

101. Singh, S., et al., *Prevalence and severity of asthma among Indian school children aged between 6 and 14 years: associations with parental smoking and traffic pollution.* J Asthma, 2016. **53**(3): p. 238-44.

102. Idris, I.B., et al., *Environmental Air Pollutants as Risk Factors for Asthma Among Children Seen in Pediatric Clinics in UKMMC, Kuala Lumpur.* Ann Glob Health, 2016. **82**(1): p. 202-8.

103. Lin, Z., et al., *Home Dampness Signs in Association with Asthma and Allergic Diseases in 4618 Preschool Children in Urumqi, China-The Influence of Ventilation/Cleaning Habits.* PLoS One, 2015. **10**(7): p. e0134359.

104. Hu, Y., et al., *Home dampness, childhood asthma, hay fever, and airway symptoms in Shanghai, China: associations, dose-response relationships, and lifestyle's influences.* Indoor Air, 2014. **24**(5): p. 450-63.

105. Dong, G.H., et al., *Home renovation, family history of atopy, and respiratory symptoms and asthma among children living in China.* Am J Public Health, 2014. **104**(10): p. 1920-7.

106. Nahhas, M., et al., *Investigating the association between obesity and asthma in 6- to 8-year-old Saudi children: a matched case-control study.* NPJ Prim Care Respir Med, 2014. **24**: p. 14004.

107. Wang, J., et al., *Rhinitis symptoms and asthma among parents of preschool children in relation to the home environment in Chongqing, China.* PLoS One, 2014. **9**(4): p. e94731.

108. Middleton, N., et al., *Prevalence of asthma and respiratory symptoms in 15-17 year-old Greek-Cypriots by proximity of their community of residence to power plants: Cyprus 2006-07.* Public Health, 2014. **128**(3): p. 288-96.

109. Zhang, M., et al., *Indoor environmental quality and the prevalence of childhood asthma and rhinitis in Wuhan area of China.* CHINESE SCIENCE BULLETIN, 2013. **58**(34): p. 7.

110. Wang, H., et al., *Dampness in dwellings and its associations with asthma and allergies among children in Chongqing: A cross-sectional study.* CHINESE SCIENCE BULLETIN, 2013. **58**(34): p. 8.

111. Dhabadi, B.B., et al., *Prevalence of asthma and associated factors among schoolchildren in rural South India.* Int J Tuberc Lung Dis, 2012. **16**(1): p. 120-5.

112. Chen, Y.C., C.H. Tsai, and Y.L. Lee, *Early-life indoor environmental exposures increase the risk of childhood asthma.* Int J Hyg Environ Health, 2011. **215**(1): p. 19-25.

113. Zuraimi, M.S., et al., *Home air-conditioning, traffic exposure, and asthma and allergic symptoms among preschool children.* Pediatr Allergy Immunol, 2011. **22**(1 Pt 2): p. e112-8.

114. Musharrafieh, U., et al., *Prevalence of asthma, allergic rhinitis and eczema among Lebanese adolescents.* J Asthma, 2009. **46**(4): p. 382-7.

115. Subramanian, S.V., et al., *Domestic violence is associated with adult and childhood asthma prevalence in India.* Int J Epidemiol, 2007. **36**(3): p. 569-79.

116. Tsai, H.J., et al., *Risk factors for respiratory symptoms and asthma in the residential environment of 5th grade schoolchildren in Taipei, Taiwan.* J Asthma, 2006. **43**(5): p. 355-61.

117. Vedanthan, P.K., et al., *Effect of animal contact and microbial exposures on the prevalence of atopy and asthma in urban vs rural children in India.* Ann Allergy Asthma Immunol, 2006. **96**(4): p. 571-8.

118. Salo, P.M., et al., *Indoor allergens, asthma, and asthma-related symptoms among adolescents in Wuhan, China.* Ann Epidemiol, 2004. **14**(8): p. 543-50.

119. Wong, G.W., et al., *Factors associated with difference in prevalence of asthma in children from three cities in China: multicentre epidemiological survey.* BMJ, 2004. **329**(7464): p. 486.

120. Mishra, V., *Effect of obesity on asthma among adult Indian women.* Int J Obes Relat Metab Disord, 2004. **28**(8): p. 1048-58.

121. Zheng, T., et al., *Childhood asthma in Beijing, China: a population-based case-control study.* Am J Epidemiol, 2002. **156**(10): p. 977-83.

122. Melsom, T., et al., *Asthma and indoor environment in Nepal.* Thorax, 2001. **56**(6): p. 477-81.

123. Wang, T.N., et al., *Association between indoor and outdoor air pollution and adolescent asthma from 1995 to 1996 in Taiwan.* Environ Res, 1999. **81**(3): p. 239-47.

124. Yang, C.Y., et al., *Indoor environmental risk factors and childhood asthma: a case-control study in a subtropical area.* Pediatr Pulmonol, 1998. **26**(2): p. 120-4.

125. Chu, Y.T., et al., *Extreme BMI predicts higher asthma prevalence and is associated with lung function impairment in school-aged children.* Pediatr Pulmonol, 2009. **44**(5): p. 472-9.

126. Rahimi Rad, M.H., M.E. Hejazi, and R. Behrouzian, *Asthma and other allergic diseases in 13-14-year-old schoolchildren in Urmia: an ISAAC study.* EMHJ - Eastern Mediterranean Health Journal, 2007. **13**: p. 12.

127. Wang, J., et al., *Asthma, allergic rhinitis and eczema among parents of preschool children in relation to climate, and dampness and mold in dwellings in China.* Environ Int, 2019. **130**: p. 104910.

128. Cai, J., et al., *Household dampness-related exposures in relation to childhood asthma and rhinitis in China: A multicentre observational study.* Environ Int, 2019. **126**: p. 735-746.

129. Norback, D., et al., *Sources of indoor particulate matter (PM) and outdoor air pollution in China in relation to asthma, wheeze, rhinitis and eczema among pre-school children: Synergistic effects between antibiotics use and PM10 and second hand smoke.* Environ Int, 2019. **125**: p. 252-260.

130. Liu, W., et al., *Association of building characteristics, residential heating and ventilation with asthmatic symptoms of preschool children in Shanghai: A cross-sectional study.* INDOOR AND BUILT ENVIRONMENT, 2014. **23**(2): p. 14.

131. Wang, J., et al., *Asthma and allergic rhinitis among young parents in China in relation to outdoor air pollution, climate and home environment.* Sci Total Environ, 2021. **751**: p. 141734.

132. Liu, W., et al., *Residence proximity to traffic-related facilities is associated with childhood asthma and rhinitis in Shandong, China.* Environ Int, 2020. **143**: p. 105930.

133. Cai, J., et al., *Associations of household dampness with asthma, allergies, and airway diseases among preschoolers in two cross-sectional studies in Chongqing, China: Repeated surveys in 2010 and 2019.* Environ Int, 2020. **140**: p. 105752.

134. Paudel, U. and K.P. Pant, *Beyond Smoking: Environmental Determinants of Asthma Prevalence in Western Nepal.* J Health Pollut, 2020. **10**(25): p. 200310.

135. Lee, J.Y., et al., *Effects of traffic-related air pollution on susceptibility to infantile bronchiolitis and childhood asthma: A cohort study in Korea.* J Asthma, 2018. **55**(3): p. 223-230.

136. Takaoka, M., K. Suzuki, and D. Norbäck, *The home environment of junior high school students in Hyogo, Japan-Associations with asthma, respiratory health and reported allergies.* Indoor and Built Environment, 2016. **25**(1): p. 12.

137. Al Ghamdi, B.R., et al., *Altitude and bronchial asthma in south-western Saudi Arabia.* EMHJ - Eastern Mediterranean Health Journal, 2008. **14**(1): p. 7.

138. Mishra, V., *Effect of indoor air pollution from biomass combustion on prevalence of asthma in the elderly.* Environ Health Perspect, 2003. **111**(1): p. 71-8.

139. Yazicioglu, M., et al., *Home environment and asthma in school children from the Edirne region in Turkey.* Allergol Immunopathol (Madr), 1998. **26**(1): p. 5-8.

140. Zhang, S., et al., *Higher environmental composite quality index score and risk of asthma and allergy in Northeast China.* Allergy, 2021. **76**(6): p. 1875-1879.

141. Miyashita, M., et al., *Eczema and Asthma Symptoms among Schoolchildren in Coastal and Inland Areas after the 2011 Great East Japan Earthquake: The ToMMo Child Health Study.* Tohoku J Exp Med, 2015. **237**(4): p. 297-305.

142. Moradi-Lakeh, M., et al., *Prevalence of asthma in Saudi adults: findings from a national household survey, 2013.* BMC Pulm Med, 2015. **15**: p. 77.

143. Yao, J., et al., *Relationship between obesity and sex, and prevalence of asthma-like disease and current wheeze in Han children in Nanjing, China.* J Int Med Res, 2015. **43**(1): p. 139-46.

144. Li, F., et al., *Environmental risk factor assessment: a multilevel analysis of childhood asthma in China.* World J Pediatr, 2013. **9**(2): p. 120-6.

145. Portnov, B.A., et al., *High prevalence of childhood asthma in Northern Israel is linked to air pollution by particulate matter: evidence from GIS analysis and Bayesian Model Averaging.* Int J Environ Health Res, 2012. **22**(3): p. 249-69.

146. Sahebi, L. and M. Shabestary, *The prevalence of asthma, allergic rhinitis, and eczema among middle school students in Tabriz (northwestern Iran).* TURKISH JOURNAL OF MEDICAL SCIENCES, 2011. **41**(5): p. 12.

147. El-Sharif, N.A., et al., *Geographical variations of asthma and asthma symptoms among schoolchildren aged 5 to 8 years and 12 to 15 years in Palestine: the International Study of Asthma and Allergies in Childhood (ISAAC).* Ann Allergy Asthma Immunol, 2003. **90**(1): p. 63-71.

148. El-Sharif, N., et al., *Asthma prevalence in children living in villages, cities and refugee camps in Palestine.* Eur Respir J, 2002. **19**(6): p. 1026-34.

149. Huang, S.L. and W.H. Pan, *Dietary fats and asthma in teenagers: analyses of the first Nutrition and Health Survey in Taiwan (NAHSIT).* Clin Exp Allergy, 2001. **31**(12): p. 1875-80.

150. Hijazi, N., B. Abalkhail, and A. Seaton, *Asthma and respiratory symptoms in urban and rural Saudi Arabia.* Eur Respir J, 1998. **12**(1): p. 41-4.

151. Lau, Y.L. and J. Karlberg, *Prevalence and risk factors of childhood asthma, rhinitis and eczema in Hong Kong.* J Paediatr Child Health, 1998. **34**(1): p. 47-52.

152. Leung, R., et al., *Prevalence of asthma and allergy in Hong Kong schoolchildren: an ISAAC study.* Eur Respir J, 1997. **10**(2): p. 354-60.

153. Laor, A., L. Cohen, and Y.L. Danon, *Effects of time, sex, ethnic origin, and area of residence on prevalence of asthma in Israeli adolescents.* BMJ, 1993. **307**(6908): p. 841-4.

154. Hallit, S., et al., *Correlation of types of food and asthma diagnosis in childhood: A case-control study.* J Asthma, 2018. **55**(9): p. 966-974.

155. Izuhara, Y., et al., *Mouth breathing, another risk factor for asthma: the Nagahama Study.* Allergy, 2016. **71**(7): p. 1031-6.

156. Lim, F.L., et al., *Asthma, Airway Symptoms and Rhinitis in Office Workers in Malaysia: Associations with House Dust Mite (HDM) Allergy, Cat Allergy and Levels of House Dust Mite Allergens in Office Dust.* PLoS One, 2015. **10**(4): p. e0124905.

157. Agrawal, S., N. Pearce, and S. Ebrahim, *Prevalence and risk factors for self-reported asthma in an adult Indian population: a cross-sectional survey.* Int J Tuberc Lung Dis, 2013. **17**(2): p. 275-82.

158. Ekici, A., et al., *Prevalence of self-reported asthma in urban and rural areas of Turkey.* J Asthma, 2012. **49**(5): p. 522-6.

159. Goh, D.Y., et al., *Prevalence and severity of asthma, rhinitis, and eczema in Singapore schoolchildren.* Arch Dis Child, 1996. **74**(2): p. 131-5.

160. Furuhata, M., et al., *Factors Associated with the Development of Childhood Asthma in Japan: A Nationwide Longitudinal Study.* Matern Child Health J, 2020. **24**(7): p. 911-922.

161. Mansouri, M., et al., *Prevalence of Ever Self-Reported Asthma and Associated Factors among University Students in Iran: A Population-Based Study.* Int J Prev Med, 2020. **11**: p. 54.

162. Masoompour, S.M., H. Mahdaviazad, and S.M.A. Ghayumi, *Asthma and its related socioeconomic factors: The Shiraz Adult Respiratory Disease Study 2015.* Clin Respir J, 2018. **12**(6): p. 2110-2116.

163. Shen, C.Y., et al., *The natural course of eczema from birth to age 7 years and the association with asthma and allergic rhinitis: a population-based birth cohort study.* Allergy Asthma Proc, 2013. **34**(1): p. 78-83.

164. Patel, S., et al., *Socioeconomic and demographic predictors of high blood pressure, diabetes, asthma and heart disease among adults engaged in various occupations: evidence from India.* J Biosoc Sci, 2020. **52**(5): p. 629-649.

165. Booalayan, H., et al., *Exposure to environmental tobacco smoke and prevalence of asthma among adolescents in a middle eastern country.* BMC Public Health, 2020. **20**(1): p. 1210.

166. Lim, J.H., et al., *Asthma under control is inversely related with erosive esophagitis among healthy adults.* PLoS One, 2019. **14**(1): p. e0210490.

167. Hawlader, M.D., et al., *Ascaris lumbricoids Infection as a Risk Factor for Asthma and Atopy in Rural Bangladeshi Children.* Trop Med Health, 2014. **42**(2): p. 77-85.

168. Lau, Y.L., J. Karlberg, and C.Y. Yeung, *Prevalence of and factors associated with childhood asthma in Hong Kong.* Acta Paediatr, 1995. **84**(7): p. 820-2.

169. Agrawal, S., *Effect of indoor air pollution from biomass and solid fuel combustion on prevalence of self-reported asthma among adult men and women in India: findings from a nationwide large-scale cross-sectional survey.* J Asthma, 2012. **49**(4): p. 355-65.

170. Guddattu, V., A. Swathi, and N.S. Nair, *Household and environment factors associated with asthma among Indian women: a multilevel approach.* J Asthma, 2010. **47**(4): p. 407-11.

171. Ho, W.C., et al., *Air pollution, weather, and associated risk factors related to asthma prevalence and attack rate.* Environ Res, 2007. **104**(3): p. 402-9.

172. Celedon, J.C., et al., *Asthma, rhinitis, and skin test reactivity to aeroallergens in families of asthmatic subjects in Anqing, China.* Am J Respir Crit Care Med, 2001. **163**(5): p. 1108-12.

173. Kashyap, G.C., S.K. Sharma, and S.K. Singh, *Prevalence and predictors of asthma, tuberculosis and chronic bronchitis among male tannery workers: A study of Kanpur City, India.* Clinical Epidemiology and Global Health, 2021. **9**: p. 7.

174. Fazlollahi, M.R., et al., *Paediatric asthma prevalence: The first national population-based survey in Iran.* Clin Respir J, 2019. **13**(1): p. 14-22.

175. Awasthi, S., P. Tripathi, and R. Prasad, *Environmental risk factors for asthma in Lucknow: A case-control study.* CLINICAL EPIDEMIOLOGY AND GLOBAL HEALTH, 2013. **1**(3): p. 9.

176. Hong, S.J., et al., *Self-reported prevalence and risk factors of asthma among Korean adolescents: 5-year follow-up study, 1995-2000.* Clin Exp Allergy, 2004. **34**(10): p. 1556-62.

177. Gupta, D., et al., *Prevalence of bronchial asthma and association with environmental tobacco smoke exposure in adolescent school children in Chandigarh, north India.* J Asthma, 2001. **38**(6): p. 501-7.

178. Tabuchi, T., et al., *Maternal and paternal indoor or outdoor smoking and the risk of asthma in their children: a nationwide prospective birth cohort study.* Drug Alcohol Depend, 2015. **147**: p. 103-8.

179. Tsai, C.H., et al., *Household environmental tobacco smoke and risks of asthma, wheeze and bronchitic symptoms among children in Taiwan.* Respir Res, 2010. **11**: p. 11.

180. Tanaka, K., et al., *Prevalence of asthma and wheeze in relation to passive smoking in Japanese children.* Ann Epidemiol, 2007. **17**(12): p. 1004-10.

181. Kim, S.Y., S. Sim, and H.G. Choi, *Active, passive, and electronic cigarette smoking is associated with asthma in adolescents.* Sci Rep, 2017. **7**.

182. Lee, A., S.Y. Lee, and K.S. Lee, *The Use of Heated Tobacco Products is Associated with Asthma, Allergic Rhinitis, and Atopic Dermatitis in Korean Adolescents.* Sci Rep, 2019. **9**(1): p. 17699.

183. Nugmanova, D., et al., *The prevalence, burden and risk factors associated with bronchial asthma in commonwealth of independent states countries (Ukraine, Kazakhstan and Azerbaijan): results of the CORE study.* BMC Pulm Med, 2018. **18**(1): p. 110.

184. Cho, J.H. and S.Y. Paik, *Association between Electronic Cigarette Use and Asthma among High School Students in South Korea.* PLoS One, 2016. **11**(3): p. e0151022.

185. Xu, X. and D.C. Christiani, *Occupational exposures and physician-diagnosed asthma.* Chest, 1993. **104**(5): p. 1364-70.

186. Xu, X., et al., *Occupational and Environmental Risk Factors for Asthma in Rural Communities in China.* Int J Occup Environ Health, 1996. **2**(3): p. 172-176.

187. Shahzad, K., S. Akhtar, and S. Mahmud, *Prevalence and determinants of asthma in adult male leather tannery workers in Karachi, Pakistan: a cross sectional study.* BMC Public Health, 2006. **6**: p. 292.

188. Chen, Y.C., et al., *Rapid adiposity growth increases risks of new-onset asthma and airway inflammation in children.* Int J Obes (Lond), 2017. **41**(7): p. 1035-1041.

189. Wang, D., et al., *Gender-specific differences in associations of overweight and obesity with asthma and asthma-related symptoms in 30 056 children: result from 25 districts of Northeastern China.* J Asthma, 2014. **51**(5): p. 508-14.

190. Chen, Y.C., et al., *Pathway from central obesity to childhood asthma. Physical fitness and sedentary time are leading factors.* Am J Respir Crit Care Med, 2014. **189**(10): p. 1194-203.

191. Okabe, Y., et al., *Association between obesity and asthma in Japanese preschool children.* Pediatr Allergy Immunol, 2012. **23**(6): p. 550-5.

192. Fukutomi, Y., et al., *Association between body mass index and asthma among Japanese adults: risk within the normal weight range.* Int Arch Allergy Immunol, 2012. **157**(3): p. 281-7.

193. Tanaka, K., et al., *U-shaped association between body mass index and the prevalence of wheeze and asthma, but not eczema or rhinoconjunctivitis: the ryukyus child health study.* J Asthma, 2011. **48**(8): p. 804-10.

194. Okabe, Y., et al., *Association of overweight with asthma symptoms in Japanese school children.* Pediatr Int, 2011. **53**(2): p. 192-8.

195. Wang, T.N., et al., *Role of gender disparity of circulating high-sensitivity C-reactive protein concentrations and obesity on asthma in Taiwan.* Clin Exp Allergy, 2011. **41**(1): p. 72-7.

196. Tsai, H.J. and A.C. Tsai, *The association of BMI and sedentary time with respiratory symptoms and asthma in 5th grade schoolchildren in Kaohsiung, Taiwan.* J Asthma, 2009. **46**(1): p. 9-15.

197. Celedon, J.C., et al., *Body mass index and asthma in adults in families of subjects with asthma in Anqing, China.* Am J Respir Crit Care Med, 2001. **164**(10 Pt 1): p. 1835-40.

198. Tomita, Y., et al., *Obesity, but not metabolic syndrome, as a risk factor for late-onset asthma in Japanese women.* Allergol Int, 2019. **68**(2): p. 240-246.

199. Chen, Y.C., et al., *Life course body mass index through childhood and young adulthood and risks of asthma and pulmonary function impairment.* Pediatr Pulmonol, 2021. **56**(5): p. 849-857.

200. Lai, L., et al., *Association between Physician-Diagnosed Asthma and Weight Status among Chinese Children: The Roles of Lifestyle Factors.* Int J Environ Res Public Health, 2020. **17**(5).

201. Myung, J., et al., *Relationships between self-reported asthma and pulmonary function and various measures of obesity.* J Asthma, 2018. **55**(7): p. 741-749.

202. Lim, M.S., et al., *Physical Activity, Sedentary Habits, Sleep, and Obesity are Associated with Asthma, Allergic Rhinitis, and Atopic Dermatitis in Korean Adolescents.* Yonsei Med J, 2017. **58**(5): p. 1040-1046.

203. Gordon, B., et al., *Association between asthma and body mass index and socioeconomic status: A cross-sectional study on 849,659 adolescents.* Respirology, 2016. **21**(1): p. 95-101.

204. Byun, E.J., et al., *Suboptimal vitamin D status in Korean adolescents: a nationwide study on its prevalence, risk factors including cotinine-verified smoking status and association with atopic dermatitis and asthma.* BMJ Open, 2017. **7**(7): p. e016409.

205. Huang, C., et al., *Pet-keeping and its impact on asthma and allergies among preschool children in Shanghai, China.* CHINESE SCIENCE BULLETIN, 2013. **58**(34): p. 8.

206. Luo, S., et al., *Pet keeping in childhood and asthma and allergy among children in Tianjin area, China.* PLoS One, 2018. **13**(5): p. e0197274.

207. Zhang, H.L., et al., *Association of pet-keeping in home with self-reported asthma and asthma-related symptoms in 11611 school children from China.* J Asthma, 2020: p. 1-10.

208. Son, J.Y., H. Kim, and M.L. Bell, *Does urban land-use increase risk of asthma symptoms?* Environ Res, 2015. **142**: p. 309-18.

209. Norback, D., et al., *Asthma, eczema, and reports on pollen and cat allergy among pupils in Shanxi province, China.* Int Arch Occup Environ Health, 2007. **80**(3): p. 207-16.

210. Deng, Q., et al., *Exposure to outdoor air pollution during trimesters of pregnancy and childhood asthma, allergic rhinitis, and eczema.* Environ Res, 2016. **150**: p. 119-127.

211. Zhao, S., et al., *Evidence of provincial variability in air pollutants-asthma relations in China.* JOURNAL OF CLEANER PRODUCTION, 2020. **242**(10.1016/j.jclepro.2019.118553).

212. Chen, F., et al., *The effects of PM2.5 on asthmatic and allergic diseases or symptoms in preschool children of six Chinese cities, based on China, Children, Homes and Health (CCHH) project.* Environ Pollut, 2018. **232**: p. 329-337.

213. Deng, Q., et al., *Early life exposure to ambient air pollution and childhood asthma in China.* Environ Res, 2015. **143**(Pt A): p. 83-92.

214. Chen, B.Y., et al., *Changes in the relationship between childhood asthma and ambient air pollution in Taiwan: Results from a nationwide survey repeated 5 years apart.* Pediatr Allergy Immunol, 2019. **30**(2): p. 188-194.

215. Deng, Q., et al., *Parental stress and air pollution increase childhood asthma in China.* Environ Res, 2018. **165**: p. 23-31.

216. Yang, S.I., et al., *Prenatal particulate matter affects new asthma via airway hyperresponsiveness in schoolchildren.* Allergy, 2019. **74**(4): p. 675-684.

217. Hasunuma, H., et al., *Association between traffic-related air pollution and asthma in preschool children in a national Japanese nested case–control study.* BMJ Open, 2016. **6**(2): p. e010410.

218. Huang, C., et al., *Breastfeeding and timing of first dietary introduction in relation to childhood asthma, allergies, and airway diseases: A cross-sectional study.* J Asthma, 2017. **54**(5): p. 488-497.

219. Chen, Y.C., C.H. Tsai, and Y. Lee, *Gestational medication use, birth conditions, and early postnatal exposures for childhood asthma.* Clin Dev Immunol, 2012. **2012**: p. 913426.

220. S.A.L., A.F. and A.D. A.M., *Risk Factors for Asthma among Preschool Children at Al-Najaf, Iraq: A Case Control Study.* Pakistan Journal of Medical & Health Science, 2020. **14**(3): p. 5.

221. Watanabe, J.I., et al., *Breastfeeding duration is inversely associated with asthma in Japanese children aged 3 years.* J Asthma, 2018. **55**(5): p. 511-516.

222. Arif, A.A. and E.F. Racine, *Does longer duration of breastfeeding prevent childhood asthma in low-income families?* J Asthma, 2017. **54**(6): p. 600-605.

223. Miyake, Y., et al., *Breastfeeding and the risk of wheeze and asthma in Japanese infants: the Osaka Maternal and Child Health Study.* Pediatr Allergy Immunol, 2008. **19**(6): p. 490-6.

224. Takaoka, M. and D. Norback, *Diet among Japanese female university students and asthmatic symptoms, infections, pollen and furry pet allergy.* Respir Med, 2008. **102**(7): p. 1045-54.

225. Lee, S.C., et al., *Risk of asthma associated with energy-dense but nutrient-poor dietary pattern in Taiwanese children.* Asia Pac J Clin Nutr, 2012. **21**(1): p. 73-81.

226. Tsai, H.J. and A.C. Tsai, *The association of diet with respiratory symptoms and asthma in schoolchildren in Taipei, Taiwan.* J Asthma, 2007. **44**(8): p. 599-603.

227. Takemura, Y., et al., *The relationship between fish intake and the prevalence of asthma: the Tokorozawa childhood asthma and pollinosis study.* Prev Med, 2002. **34**(2): p. 221-5.

228. Ibrahim, A.A., et al., *Association between Soft Drink Consumption and Asthma among Qatari Adults.* Nutrients, 2019. **11**(3).

229. Fu, Q.L., et al., *Prevalence and Occupational and Environmental Risk Factors of Self-Reported Asthma: Evidence from a Cross-Sectional Survey in Seven Chinese Cities.* Int J Environ Res Public Health, 2016. **13**(11).

230. Krstev, S., et al., *Occupation and adult-onset asthma among Chinese women in a population-based cohort.* Am J Ind Med, 2007. **50**(4): p. 265-73.

231. Ballal, S.G., et al., *Bronchial asthma in two chemical fertilizer producing factories in eastern Saudi Arabia.* Int J Tuberc Lung Dis, 1998. **2**(4): p. 330-5.

232. Agrawal, S., et al., *Occupations with an increased prevalence of self-reported asthma in Indian adults.* J Asthma, 2014. **51**(8): p. 814-24.

233. Yamamoto-Hanada, K., et al., *Influence of antibiotic use in early childhood on asthma and allergic diseases at age 5.* Ann Allergy Asthma Immunol, 2017. **119**(1): p. 54-58.

234. Zou, Z., et al., *First-Year Antibiotics Exposure in Relation to Childhood Asthma, Allergies, and Airway Illnesses.* Int J Environ Res Public Health, 2020. **17**(16).

235. Lu, F.L., et al., *Body mass index may modify asthma prevalence among low-birth-weight children.* Am J Epidemiol, 2012. **176**(1): p. 32-42.

236. Takata, N., et al., *Preterm birth is associated with higher prevalence of wheeze and asthma in a selected population of Japanese children aged three years.* ALLERGOLOGIA ET IMMUNOPATHOLOGIA, 2019. **47**(5): p. 6.

237. Raheleh, Z., et al., *The Association between Birth Weight and Gestational Age and Asthma in 6-7- and 13-14-Year-Old Children.* Scientifica (Cairo), 2016. **2016**: p. 3987460.

238. Waked, M. and P. Salameh, *Asthma, allergic rhinitis and eczema in 5-12-year-old school children across Lebanon.* Public Health, 2008. **122**(9): p. 965-73.

239. Zhao, T., et al., *Prevalence of childhood asthma, allergic rhinitis and eczema in Urumqi and Beijing.* J Paediatr Child Health, 2000. **36**(2): p. 128-33.

240. Chew, F.T., D.Y. Goh, and B.W. Lee, *Geographical comparison of the prevalence of childhood asthma and allergies in Singapore.* Ann Trop Paediatr, 1999. **19**(4): p. 383-90.

241. Chen, G., et al., *Associations of caesarean delivery and the occurrence of neurodevelopmental disorders, asthma or obesity in childhood based on Taiwan birth cohort study.* BMJ Open, 2017. **7**(9): p. e017086.

242. Lavin, T., P. Franklin, and D.B. Preen, *Association between Caesarean Delivery and Childhood Asthma in India and Vietnam.* Paediatr Perinat Epidemiol, 2017. **31**(1): p. 47-54.

243. Chu, S., et al., *Cesarean section without medical indication and risk of childhood asthma, and attenuation by breastfeeding.* PLoS One, 2017. **12**(9): p. e0184920.

244. Metintas, S., E. Kurt, and P.S. Group, *Geo-climate effects on asthma and allergic diseases in adults in Turkey: results of PARFAIT study.* Int J Environ Health Res, 2010. **20**(3): p. 189-99.

245. Huang, X., et al., *Association between Concentrations of Metals in Urine and Adult Asthma: A Case-Control Study in Wuhan, China.* PLoS One, 2016. **11**(5): p. e0155818.

246. Hsu, N.Y., et al., *Predicted risk of childhood allergy, asthma, and reported symptoms using measured phthalate exposure in dust and urine.* Indoor Air, 2012. **22**(3): p. 186-99.

247. Wang, I.J., W.J. Karmaus, and C.C. Yang, *Polycyclic aromatic hydrocarbons exposure, oxidative stress, and asthma in children.* Int Arch Occup Environ Health, 2017. **90**(3): p. 297-303.

248. Ma, Y.N., et al., *Association of urine CC16 and lung function and asthma in Chinese children.* Allergy Asthma Proc, 2015. **36**(4): p. 59-64.

249. Shi, W., et al., *Urinary phthalate metabolites in relation to childhood asthmatic and allergic symptoms in Shanghai.* Environ Int, 2018. **121**(Pt 1): p. 276-286.

250. Huang, X., et al., *Urinary polycyclic aromatic hydrocarbon metabolites and adult asthma: a case-control study.* Sci Rep, 2018. **8**(1): p. 7658.

251. Ku, H.Y., et al., *Prenatal and postnatal exposure to phthalate esters and asthma: a 9-year follow-up study of a taiwanese birth cohort.* PLoS One, 2015. **10**(4): p. e0123309.

252. Meng, G., et al., *Typical halogenated persistent organic pollutants in indoor dust and the associations with childhood asthma in Shanghai, China.* Environ Pollut, 2016. **211**: p. 389-98.

253. Ait Bamai, Y., et al., *Exposure to house dust phthalates in relation to asthma and allergies in both children and adults.* Sci Total Environ, 2014. **485-486**: p. 153-163.

254. Bener, A., et al., *The impact of Vitamin D deficiency on asthma, allergic rhinitis and wheezing in children: An emerging public health problem.* J Family Community Med, 2014. **21**(3): p. 154-61.
