# Supplementary material for: Risk factors of asthma in the Asian population: a systematic review and meta-analysis
Source: J Physiol Anthropol. 2021 Dec 9;40:22. doi: 10.1186/s40101-021-00273-x (PMC8662898; doi:10.1186/s40101-021-00273-x)
Supplement: Supplementary file 7 — Additional file 7: Table S6. Summary of frequently reported asthma comorbidities in the Asian population (1993-2021). [file 40101_2021_273_MOESM7_ESM.docx]

**Supplementary Table S6.** Summary of Frequently Reported Asthma Comorbidities in the Asian Population (1993-2021).

| **Comorbidities** | **No. of studies with significant associations** | **Ref^1^** | **No. of studies with mixed/insignificant associations** | **Ref^2^** |
| --- | --- | --- | --- | --- |
| **Total Publications** | **54** | **-** | **6** | **-** |
| Atopy/Allergen Sensitization | 23 | [1-23] | 3 | [24-26] |
| Allergic Rhinitis / Rhinitis / Hay Fever / Rhinoconjunctivitis | 21 | [4, 11, 24, 27-44] | 0 | - |
| Respiratory Infections | 20 | [12, 22, 29, 34, 42, 44-58] | 0 | - |
| Eczema / Atopic Dermatitis / Itchy Rash | 18 | [22, 29, 30, 32, 38-44, 55, 57, 59-63] | 2 | [47, 64] |
| Gastroesophageal reflux disease (GERD) | 5 | [4, 29, 32, 52, 65] | 0 | - |
| Chronic rhinosinusitis, Sinusitis | 5 | [4, 42, 61, 66, 67] | 0 | - |
| Food Allergy | 4 | [11, 47, 61, 67] | 0 | - |
| Bronchitis | 3 | [29, 42, 68] | 0 | - |
| Otitis | 3 | [52, 61, 67] | 0 | - |

Only comorbidities with significant asthma-association reported in >= three independent studies were listed. All reference numbers mentioned in this table referred to bibliographies listed in the references section below. **Ref^1^**: studies reported a significant comorbidity with asthma; **Ref^2^**: studies reported a mixed or insignificant comorbidity with asthma.

**References**

1. Alqahtani JM, Asaad AM, Awadalla NJ, Mahfouz AA: **Environmental Determinants of Bronchial Asthma among Saudi School Children in Southwestern Saudi Arabia**. *Int J Environ Res Public Health* 2016, **14**(1).

2. Takaoka M, Suzuki K, Norback D: **Current asthma, respiratory symptoms and airway infections among students in relation to the school and home environment in Japan**. *J Asthma* 2017, **54**(6):652-661.

3. Alqahtani JM: **Asthma and other allergic diseases among Saudi schoolchildren in Najran: the need for a comprehensive intervention program**. *Ann Saudi Med* 2016, **36**(6):379-385.

4. Izuhara Y, Matsumoto H, Nagasaki T, Kanemitsu Y, Murase K, Ito I, Oguma T, Muro S, Asai K, Tabara Y *et al*: **Mouth breathing, another risk factor for asthma: the Nagahama Study**. *Allergy* 2016, **71**(7):1031-1036.

5. Wortong D, Chaiear N, Boonsawat W: **Risk of asthma in relation to occupation: A hospital-based case-control study**. *Asian Pac J Allergy Immunol* 2015, **33**(2):152-160.

6. Lim FL, Hashim Z, Than LT, Md Said S, Hisham Hashim J, Norback D: **Asthma, Airway Symptoms and Rhinitis in Office Workers in Malaysia: Associations with House Dust Mite (HDM) Allergy, Cat Allergy and Levels of House Dust Mite Allergens in Office Dust**. *PLoS One* 2015, **10**(4):e0124905.

7. Chae Y, Hahm MI, Ahn K, Kim J, Kim WK, Lee SY, Park YM, Han MY, Lee KJ, Kwon HJ: **Indoor environmental factors associated with wheezing illness and asthma in South Korean children: phase III of the International Study of Asthma and Allergies in Childhood**. *J Asthma* 2014, **51**(9):943-949.

8. Feng M, Yang Z, Pan L, Lai X, Xian M, Huang X, Chen Y, Schroder PC, Roponen M, Schaub B *et al*: **Associations of Early Life Exposures and Environmental Factors With Asthma Among Children in Rural and Urban Areas of Guangdong, China**. *Chest* 2016, **149**(4):1030-1041.

9. Bener A, Ehlayel MS, Bener HZ, Hamid Q: **The impact of Vitamin D deficiency on asthma, allergic rhinitis and wheezing in children: An emerging public health problem**. *J Family Community Med* 2014, **21**(3):154-161.

10. Wang D, Xiao W, Ma D, Zhang Y, Wang Q, Wang C, Ji X, He B, Wu X, Chen H *et al*: **Cross-sectional epidemiological survey of asthma in Jinan, China**. *Respirology* 2013, **18**(2):313-322.

11. Ding YP, Yao HX, Tang XL, He HW, Shi HF, Lin L, Li M, Chen S, Chen J, Wang HJ: **An epidemiology study of bronchial asthma in the Li ethnic group in China**. *Asian Pac J Trop Med* 2012, **5**(2):157-161.

12. Al-Mousawi MS, Lovel H, Behbehani N, Arifhodzic N, Woodcock A, Custovic A: **Asthma and sensitization in a community with low indoor allergen levels and low pet-keeping frequency**. *J Allergy Clin Immunol* 2004, **114**(6):1389-1394.

13. Palmer LJ, Celedon JC, Weiss ST, Wang B, Fang Z, Xu X: **Ascaris lumbricoides infection is associated with increased risk of childhood asthma and atopy in rural China**. *Am J Respir Crit Care Med* 2002, **165**(11):1489-1493.

14. Leung TF, Lam CW, Chan IH, Li AM, Ha G, Tang NL, Fok TF: **Inhalant allergens as risk factors for the development and severity of mild-to-moderate asthma in Hong Kong Chinese children**. *J Asthma* 2002, **39**(4):323-330.

15. Celedon JC, Palmer LJ, Xu X, Wang B, Fang Z, Weiss ST: **Sensitization to silk and childhood asthma in rural China**. *Pediatrics* 2001, **107**(5):E80.

16. Hijazi N, Abalkhail B, Seaton A: **Diet and childhood asthma in a society in transition: a study in urban and rural Saudi Arabia**. *Thorax* 2000, **55**(9):775-779.

17. Leung R, Ho P, Lam CW, Lai CK: **Sensitization to inhaled allergens as a risk factor for asthma and allergic diseases in Chinese population**. *J Allergy Clin Immunol* 1997, **99**(5):594-599.

18. Goh DY, Chew FT, Quek SC, Lee BW: **Prevalence and severity of asthma, rhinitis, and eczema in Singapore schoolchildren**. *Arch Dis Child* 1996, **74**(2):131-135.

19. Leung R, Ho P: **Asthma, allergy, and atopy in three south-east Asian populations**. *Thorax* 1994, **49**(12):1205-1210.

20. Boker F, Alzahrani A, Alsaeed A, Alzhrani M, Albar R: **Cesarean Section and Development of Childhood Bronchial Asthma: Is There A Risk?** *Open Access Maced J Med Sci* 2019, **7**(3):347-351.

21. Chu YT, Chen WY, Wang TN, Tseng HI, Wu JR, Ko YC: **Extreme BMI predicts higher asthma prevalence and is associated with lung function impairment in school-aged children**. *Pediatr Pulmonol* 2009, **44**(5):472-479.

22. Lau YL, Karlberg J, Yeung CY: **Prevalence of and factors associated with childhood asthma in Hong Kong**. *Acta Paediatr* 1995, **84**(7):820-822.

23. Celedon JC, Palmer LJ, Weiss ST, Wang B, Fang Z, Xu X: **Asthma, rhinitis, and skin test reactivity to aeroallergens in families of asthmatic subjects in Anqing, China**. *Am J Respir Crit Care Med* 2001, **163**(5):1108-1112.

24. Oshikata C, Watanabe M, Ishida M, Kobayashi S, Kubosaki A, Yamazaki A, Konuma R, Hashimoto K, Kobayashi N, Kaneko T *et al*: **Increase in asthma prevalence in adults in temporary housing after the Great East Japan earthquake**. *International Journal of Disaster Risk Reduction* 2020, **50**.

25. Hawlader MD, Ma E, Noguchi E, Itoh M, Arifeen SE, Persson LA, Moore SE, Raqib R, Wagatsuma Y: **Ascaris lumbricoids Infection as a Risk Factor for Asthma and Atopy in Rural Bangladeshi Children**. *Trop Med Health* 2014, **42**(2):77-85.

26. Sundaru H: **House dust mite allergen level and allergen sensitization as risk factors for asthma among student in Central Jakarta**. *Medical Journal of Indonesia* 2006, **15**(1):5.

27. Huang K, Yang T, Xu J, Yang L, Zhao J, Zhang X, Bai C, Kang J, Ran P, Shen H *et al*: **Prevalence, risk factors, and management of asthma in China: a national cross-sectional study**. *Lancet* 2019, **394**(10196):407-418.

28. Masoompour SM, Mahdaviazad H, Ghayumi SMA: **Asthma and its related socioeconomic factors: The Shiraz Adult Respiratory Disease Study 2015**. *Clin Respir J* 2018, **12**(6):2110-2116.

29. Lin J, Wang W, Chen P, Zhou X, Wan H, Yin K, Ma L, Wu C, Li J, Liu C *et al*: **Prevalence and risk factors of asthma in mainland China: The CARE study**. *Respir Med* 2018, **137**:48-54.

30. Qureshi UA, Bilques S, Ul Haq I, Khan MS, Qurieshi MA, Qureshi UA: **Epidemiology of bronchial asthma in school children (10-16 years) in Srinagar**. *Lung India* 2016, **33**(2):167-173.

31. Danansuriya MN, Rajapaksa LC, Weerasinghe A: **Genetic, familial and environmental correlates of asthma among early adolescents in Sri Lanka: a case control study**. *World Allergy Organ J* 2015, **8**(1):19.

32. Li F, Zhou YC, Tong SL, Li SH, Jiang F, Jin XM, Yan CH, Tian Y, Deng SN, Shen XM: **Environmental risk factor assessment: a multilevel analysis of childhood asthma in China**. *World J Pediatr* 2013, **9**(2):120-126.

33. Higuchi O, Adachi Y, Itazawa T, Ito Y, Yoshida K, Ohya Y, Odajima H, Akasawa A, Miyawaki T: **Rhinitis has an association with asthma in school children**. *Am J Rhinol Allergy* 2013, **27**(1):e22-25.

34. Ekici A, Ekici M, Kocyigit P, Karlidag A: **Prevalence of self-reported asthma in urban and rural areas of Turkey**. *J Asthma* 2012, **49**(5):522-526.

35. Al Ghobain MO, Al-Hajjaj MS, Al Moamary MS: **Asthma prevalence among 16- to 18-year-old adolescents in Saudi Arabia using the ISAAC questionnaire**. *BMC Public Health* 2012, **12**:239.

36. Cakir E, Ersu R, Uyan ZS, Oktem S, Varol N, Karakoc F, Karadag B, Akyol M, Dagli E: **The prevalence and risk factors of asthma and allergic diseases among working adolescents**. *Asian Pac J Allergy Immunol* 2010, **28**(2-3):122-129.

37. Uthaisangsook S: **Risk factors for development of asthma in Thai adults in Phitsanulok: a university-based study**. *Asian Pac J Allergy Immunol* 2010, **28**(1):23-28.

38. Musharrafieh U, Al-Sahab B, Zaitoun F, El-Hajj MA, Ramadan F, Tamim H: **Prevalence of asthma, allergic rhinitis and eczema among Lebanese adolescents**. *J Asthma* 2009, **46**(4):382-387.

39. Ho WC, Hartley WR, Myers L, Lin MH, Lin YS, Lien CH, Lin RS: **Air pollution, weather, and associated risk factors related to asthma prevalence and attack rate**. *Environ Res* 2007, **104**(3):402-409.

40. Nga NN, Chai SK, Bihn TT, Redding G, Takaro T, Checkoway H, Son PH, Van DK, Keifer M, Trung le V *et al*: **ISAAC-based asthma and atopic symptoms among Ha Noi school children**. *Pediatr Allergy Immunol* 2003, **14**(4):272-279.

41. Leung R, Wong G, Lau J, Ho A, Chan JK, Choy D, Douglass C, Lai CK: **Prevalence of asthma and allergy in Hong Kong schoolchildren: an ISAAC study**. *Eur Respir J* 1997, **10**(2):354-360.

42. Moussa MA, Skaik MB, Yaghy OY, Salwanes SB, Bin-Othman SA: **Factors associated with asthma in school children**. *Eur J Epidemiol* 1996, **12**(6):583-588.

43. Rahimi Rad MH, Hejazi ME, Behrouzian R: **Asthma and other allergic diseases in 13-14-year-old schoolchildren in Urmia: an ISAAC study**. *EMHJ - Eastern Mediterranean Health Journal* 2007, **13**:12.

44. Al-Mazam A, Mohamed AG: **Risk factors of bronchial asthma in bahrah, saudi arabia**. *J Family Community Med* 2001, **8**(1):33-39.

45. Sun Y, Hou J, Sheng Y, Kong X, Weschler LB, Sundell J: **Modern life makes children allergic. A cross-sectional study: associations of home environment and lifestyles with asthma and allergy among children in Tianjin region, China**. *Int Arch Occup Environ Health* 2019, **92**(4):587-598.

46. Nugmanova D, Sokolova L, Feshchenko Y, Iashyna L, Gyrina O, Malynovska K, Mustafayev I, Aliyeva G, Makarova J, Vasylyev A *et al*: **The prevalence, burden and risk factors associated with bronchial asthma in commonwealth of independent states countries (Ukraine, Kazakhstan and Azerbaijan): results of the CORE study**. *BMC Pulm Med* 2018, **18**(1):110.

47. Huang CC, Chiang TL, Chen PC, Lin SJ, Wen HJ, Guo YL: **Risk factors for asthma occurrence in children with early-onset atopic dermatitis: An 8-year follow-up study**. *Pediatr Allergy Immunol* 2018, **29**(2):159-165.

48. Jeng MJ, Lee YS, Tsao PC, Yang CF, Soong WJ: **A longitudinal study on early hospitalized airway infections and subsequent childhood asthma**. *PLoS One* 2014, **10**(4):e0121906.

49. Chen YC, Tsai CH, Lee Y: **Gestational medication use, birth conditions, and early postnatal exposures for childhood asthma**. *Clin Dev Immunol* 2012, **2012**:913426.

50. Yeh KW, Ou LS, Yao TC, Chen LC, Lee WI, Huang JL, Group PS: **Prevalence and risk factors for early presentation of asthma among preschool children in Taiwan**. *Asian Pac J Allergy Immunol* 2011, **29**(2):120-126.

51. Fernando D, Wickramasinghe P, Kapilananda G, Dewasurendra RL, Amarasooriya M, Dayaratne A: **Toxocara seropositivity in Sri Lankan children with asthma**. *Pediatr Int* 2009, **51**(2):241-245.

52. Waked M, Salameh P: **Risk factors for asthma and allergic diseases in school children across Lebanon**. *J Asthma Allergy* 2008, **2**:1-7.

53. Zaman K, Takeuchi H, Md Y, El Arifeen S, Chowdhury HR, Baqui AH, Wakai S, Iwata T: **Asthma in rural Bangladeshi children**. *Indian J Pediatr* 2007, **74**(6):539-543.

54. S.A.L. AF, A.M. AD: **Risk Factors for Asthma among Preschool Children at Al-Najaf, Iraq: A Case Control Study**. *Pakistan Journal of Medical & Health Science* 2020, **14**(3):5.

55. Tan TN, Shek LP, Goh DY, Chew FT, Lee BW: **Prevalence of asthma and comorbid allergy symptoms in Singaporean preschoolers**. *Asian Pac J Allergy Immunol* 2006, **24**(4):175-182.

56. Demir AU, Karakaya G, Bozkurt B, Sekerel BE, Kalyoncu AF: **Asthma and allergic diseases in schoolchildren: third cross-sectional survey in the same primary school in Ankara, Turkey**. *Pediatr Allergy Immunol* 2004, **15**(6):531-538.

57. Huang SL, Tsai PF, Yeh YF: **Negative association of Enterobius infestation with asthma and rhinitis in primary school children in Taipei**. *Clin Exp Allergy* 2002, **32**(7):1029-1032.

58. Hallit S, Sacre H, Kheir N, Hobeika E, Hallit R, Waked M, Salameh P: **Hygiene hypothesis: association between hygiene and asthma among preschool children in Lebanon**. *Allergol Immunopathol (Madr)* 2021, **49**(1):135-145.

59. Shen CY, Lin MC, Lin HK, Lin CH, Fu LS, Fu YC: **The natural course of eczema from birth to age 7 years and the association with asthma and allergic rhinitis: a population-based birth cohort study**. *Allergy Asthma Proc* 2013, **34**(1):78-83.

60. Zhao T, Wang HJ, Chen Y, Xiao M, Duo L, Liu G, Lau Y, Karlberg J: **Prevalence of childhood asthma, allergic rhinitis and eczema in Urumqi and Beijing**. *J Paediatr Child Health* 2000, **36**(2):128-133.

61. Ones U, Sapan N, Somer A, Disci R, Salman N, Guler N, Yalcin I: **Prevalence of childhood asthma in Istanbul, Turkey**. *Allergy* 1997, **52**(5):570-575.

62. Jang Y, Shin A: **Sex-Based Differences in Asthma among Preschool and School-Aged Children in Korea.** *PLOS ONE* 2015, **10**(e0140057).

63. Kawada T: **Risk factors and prevalence of asthma or atopic dermatitis in young children by a questionnaire survey**. *J Nippon Med Sch* 2004, **71**(3):167-171.

64. Dongol Singh S, Shrestha A: **Risk Factors Associated with Childhood Asthma - A Case Control Study**. *Kathmandu Univ Med J (KUMJ)* 2018, **16**(64):290-295.

65. Tsai MC, Lin HL, Lin CC, Lin HC, Chen YH, Pfeiffer S, Lin HC: **Increased risk of concurrent asthma among patients with gastroesophageal reflux disease: a nationwide population-based study**. *Eur J Gastroenterol Hepatol* 2010, **22**(10):1169-1173.

66. Ostovar A, Fokkens WJ, Pordel S, Movahed A, Ghasemi K, Marzban M, Farrokhi S: **The prevalence of asthma in adult population of southwestern Iran and its association with chronic rhinosinusitis: a GA(2)LEN study**. *Clin Transl Allergy* 2019, **9**:43.

67. Ones U, Akcay A, Tamay Z, Guler N, Zencir M: **Rising trend of asthma prevalence among Turkish schoolchildren (ISAAC phases I and III)**. *Allergy* 2006, **61**(12):1448-1453.

68. Gazala E, Ron-Feldman V, Alterman M, Kama S, Novack L: **The association between birth season and future development of childhood asthma**. *Pediatr Pulmonol* 2006, **41**(12):1125-1128.
